# Supplementary material for: The Arabidopsis SGN3/GSO1 receptor kinase integrates soil nitrogen status into shoot development
Source: EMBO J. 2024 May 2;43(12):9. doi: 10.1038/s44318-024-00107-3 (PMC11183077; doi:10.1038/s44318-024-00107-3)
Supplement: Supplementary file 4 — Movie EV1 [file 44318_2024_107_MOESM4_ESM.zip › Movie EV1 legend.docx]

Movie EV1: Timeseries of CASP1-GFP expression in MYB36_Loop_#5.

5-day-old roots were imaged on a vertically oriented ZEISS LSM-980 microscope equipped with an Airyscan 2 detector. The video depicts a maximum projection of endodermal cells from before onset of CASP1-GFP expression. Time format is hh:mm:ss.
