# Supplementary figures and images for: The Arabidopsis SGN3/GSO1 receptor kinase integrates soil nitrogen status into shoot development
Source: EMBO J. 2024 May 2;43(12):9. doi: 10.1038/s44318-024-00107-3 (PMC11183077; doi:10.1038/s44318-024-00107-3)

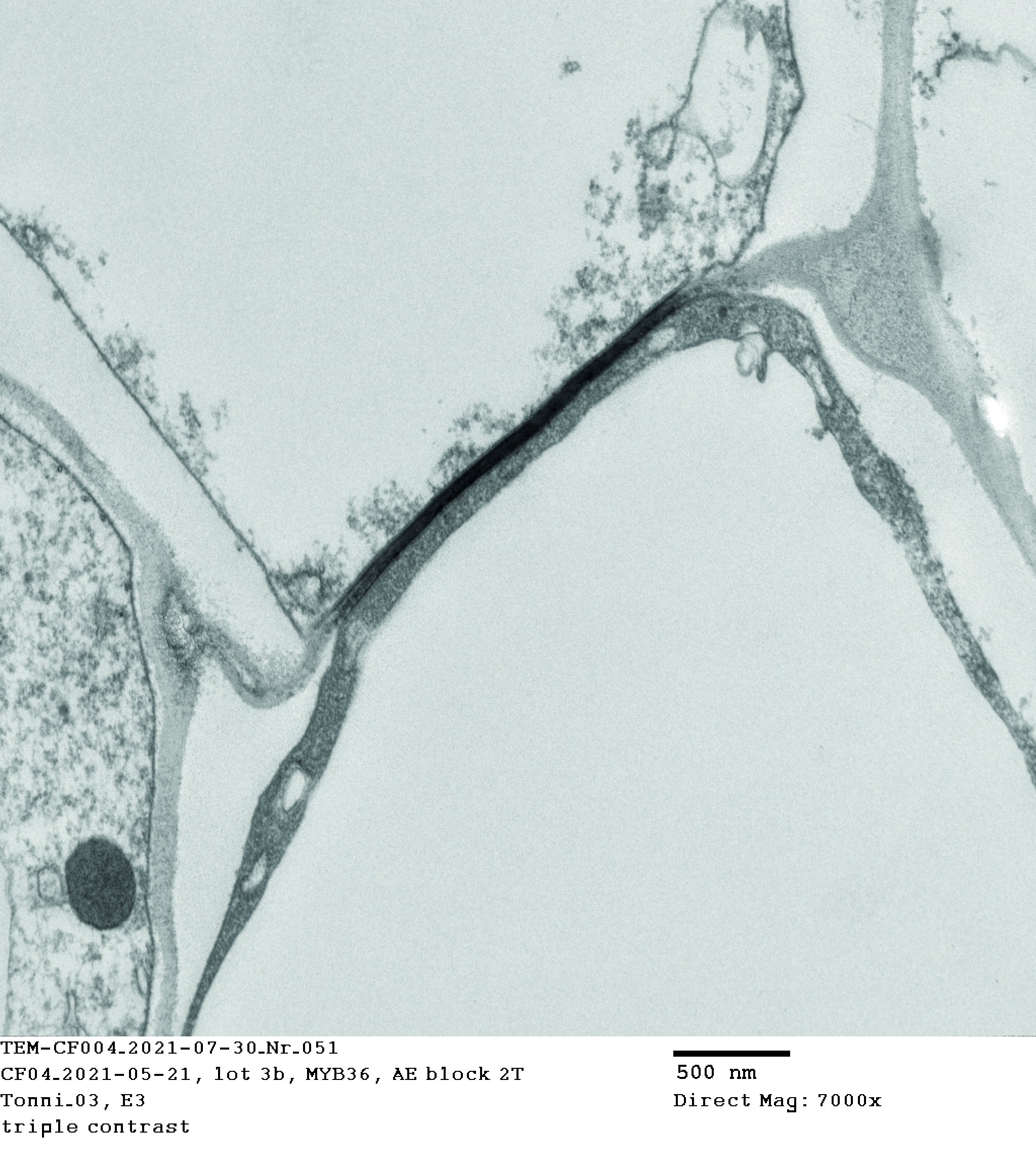

Supplement: Supplementary file 5 — Source data Fig. 1 [file 44318_2024_107_MOESM5_ESM.zip › Figure 1/Figure 1D/Fig1D_MYB.tif]

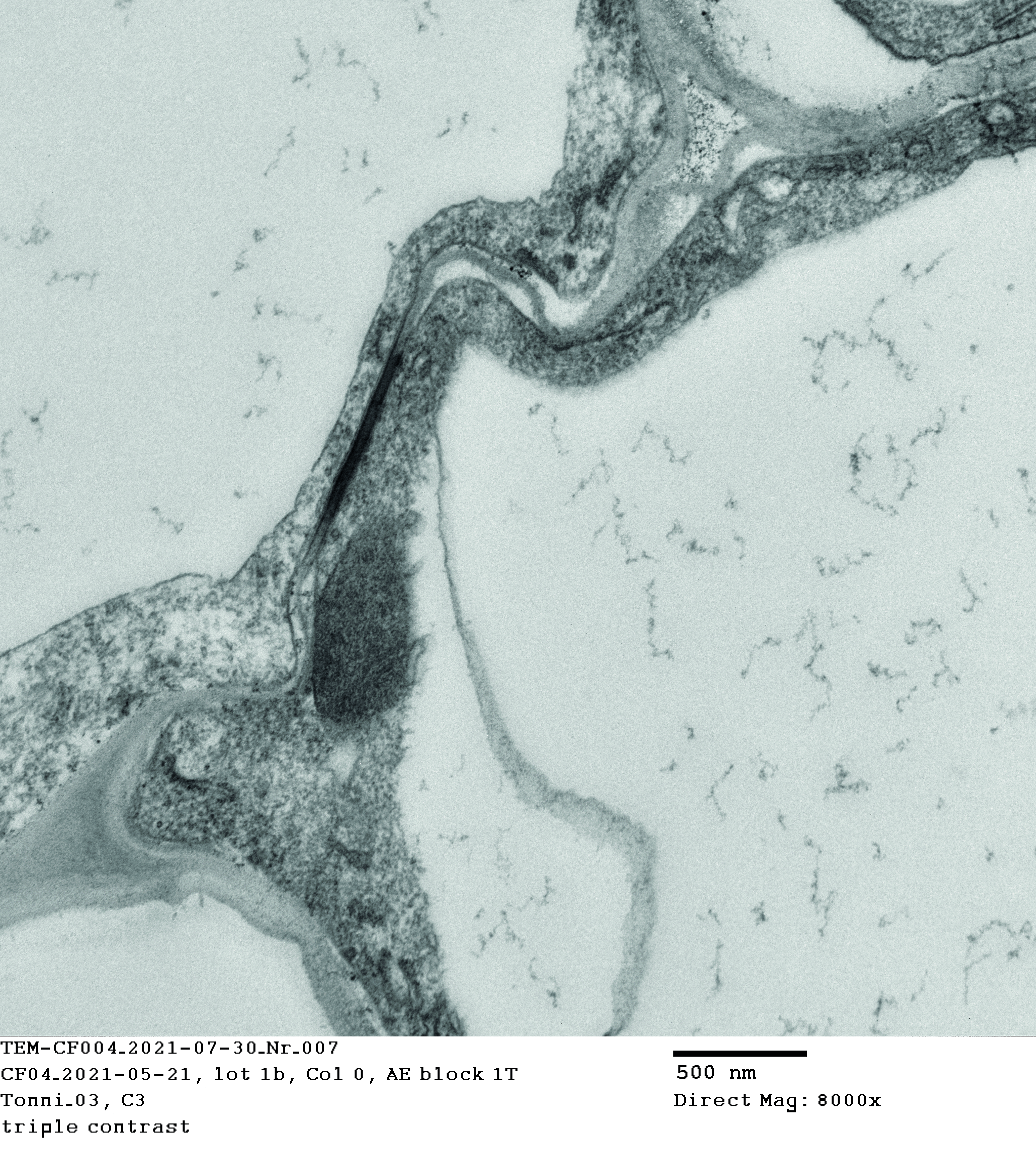

Supplement: Supplementary file 5 — Source data Fig. 1 [file 44318_2024_107_MOESM5_ESM.zip › Figure 1/Figure 1D/FIg1D_WT.tif]

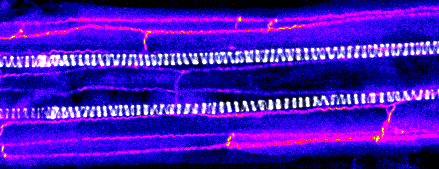

Supplement: Supplementary file 5 — Source data Fig. 1 [file 44318_2024_107_MOESM5_ESM.zip › Figure 1/Figure 1G/Fig1G_BL6_SGN3.tif]

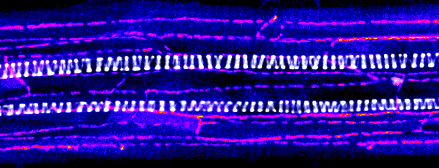

Supplement: Supplementary file 5 — Source data Fig. 1 [file 44318_2024_107_MOESM5_ESM.zip › Figure 1/Figure 1G/Fig1G_SGN3.tif]

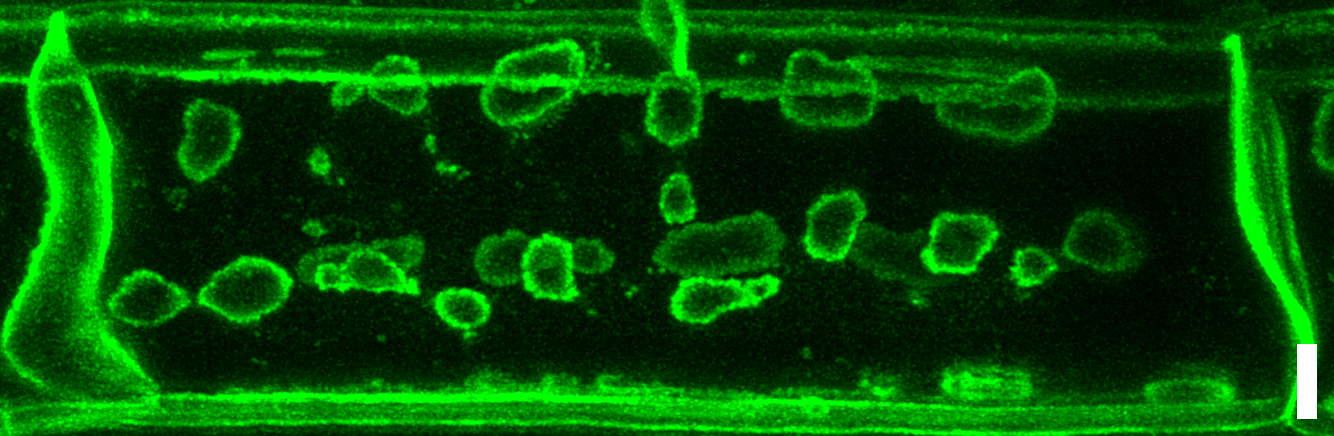

Supplement: Supplementary file 6 — Source data Fig. 2 [file 44318_2024_107_MOESM6_ESM.zip › Figure 2/Figure 2A/11h 52min_2.tif]

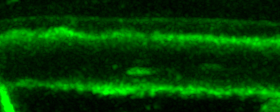

Supplement: Supplementary file 6 — Source data Fig. 2 [file 44318_2024_107_MOESM6_ESM.zip › Figure 2/Figure 2A/11h 52min__1.tif]

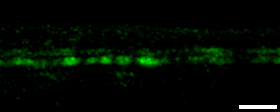

Supplement: Supplementary file 6 — Source data Fig. 2 [file 44318_2024_107_MOESM6_ESM.zip › Figure 2/Figure 2A/1h 2min_1.tif]

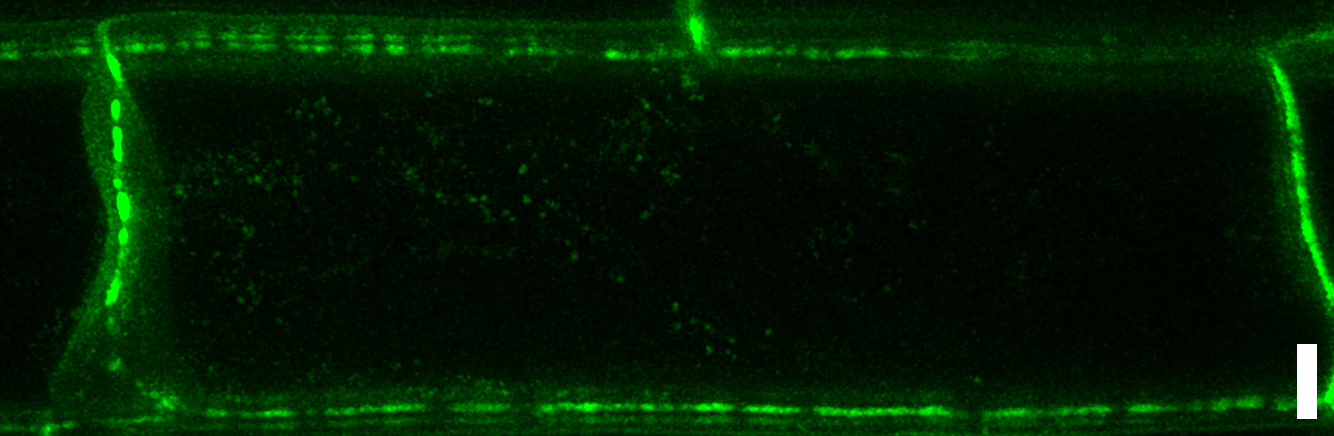

Supplement: Supplementary file 6 — Source data Fig. 2 [file 44318_2024_107_MOESM6_ESM.zip › Figure 2/Figure 2A/1h 2min_2.tif]

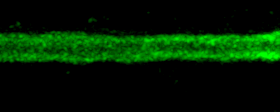

Supplement: Supplementary file 6 — Source data Fig. 2 [file 44318_2024_107_MOESM6_ESM.zip › Figure 2/Figure 2A/2h 8min_1.tif]

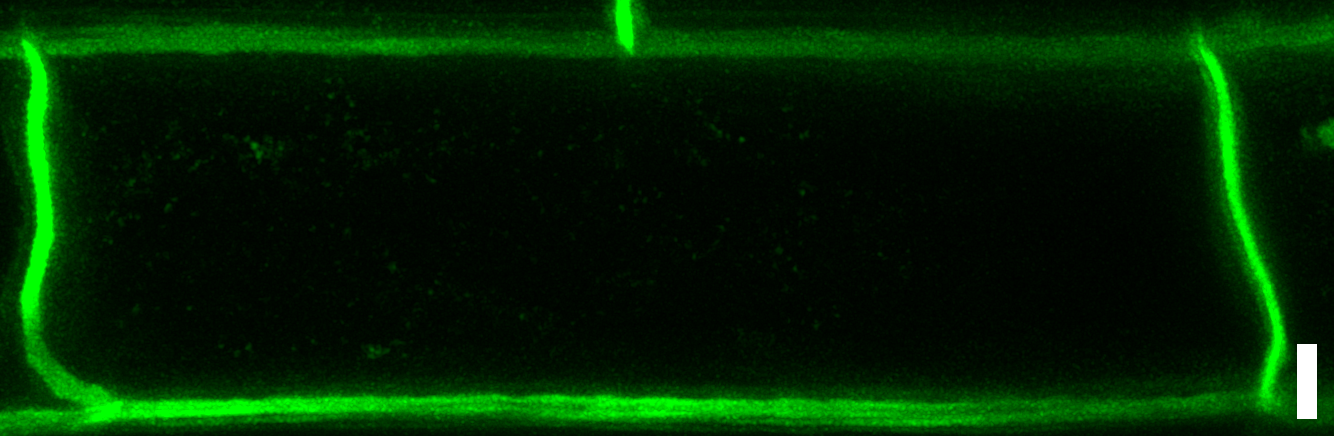

Supplement: Supplementary file 6 — Source data Fig. 2 [file 44318_2024_107_MOESM6_ESM.zip › Figure 2/Figure 2A/2h 8min_2.tif]

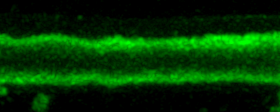

Supplement: Supplementary file 6 — Source data Fig. 2 [file 44318_2024_107_MOESM6_ESM.zip › Figure 2/Figure 2A/7h 13min_1.tif]

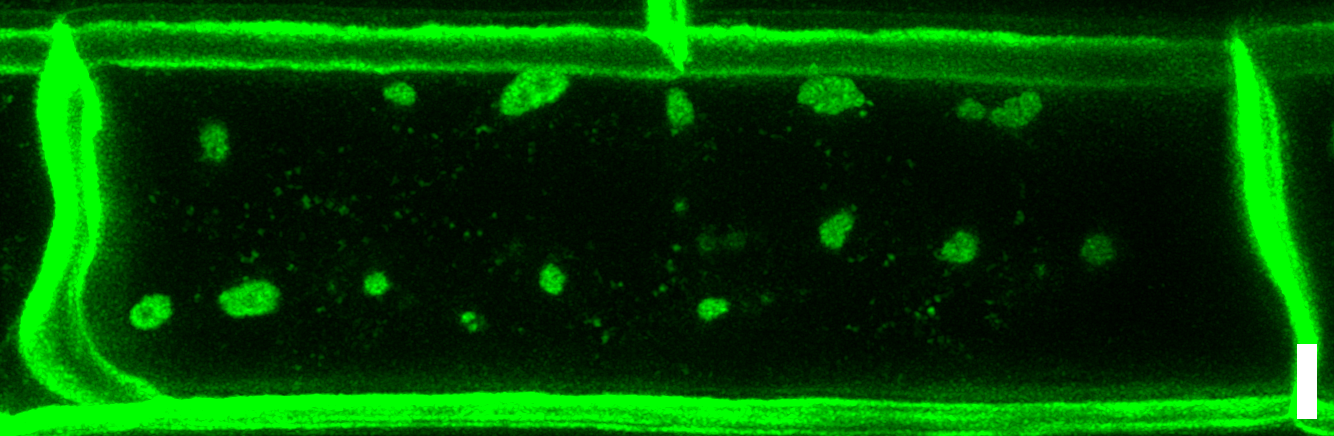

Supplement: Supplementary file 6 — Source data Fig. 2 [file 44318_2024_107_MOESM6_ESM.zip › Figure 2/Figure 2A/7h 13min_2.tif]

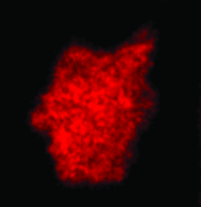

Supplement: Supplementary file 6 — Source data Fig. 2 [file 44318_2024_107_MOESM6_ESM.zip › Figure 2/Figure 2B/Basic_fuchsin.tif]

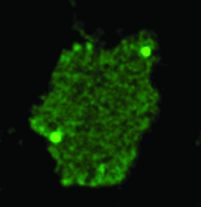

Supplement: Supplementary file 6 — Source data Fig. 2 [file 44318_2024_107_MOESM6_ESM.zip › Figure 2/Figure 2B/CASP1_GFP.tif]

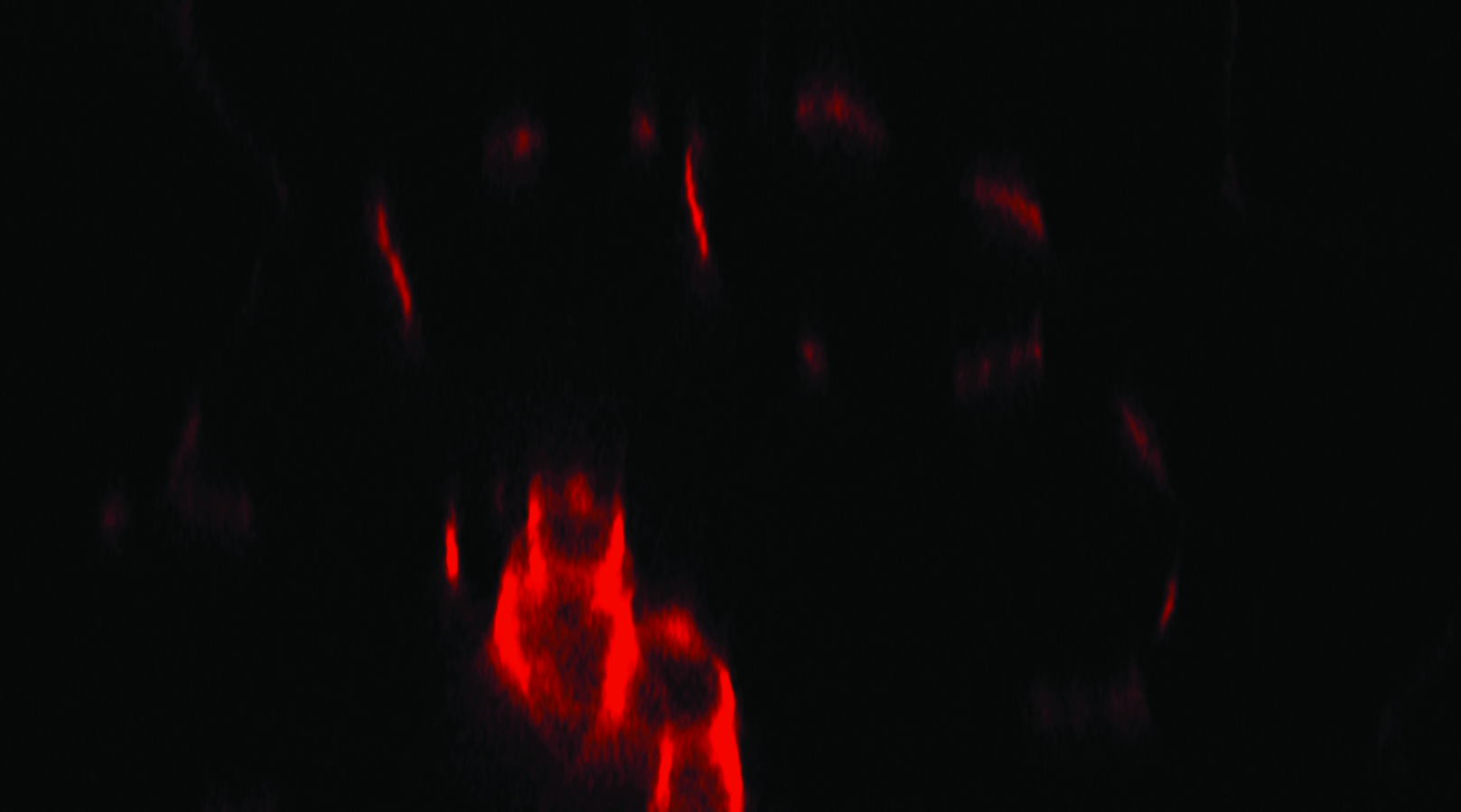

Supplement: Supplementary file 6 — Source data Fig. 2 [file 44318_2024_107_MOESM6_ESM.zip › Figure 2/Figure 2B/insert_MYB_BF.tif]

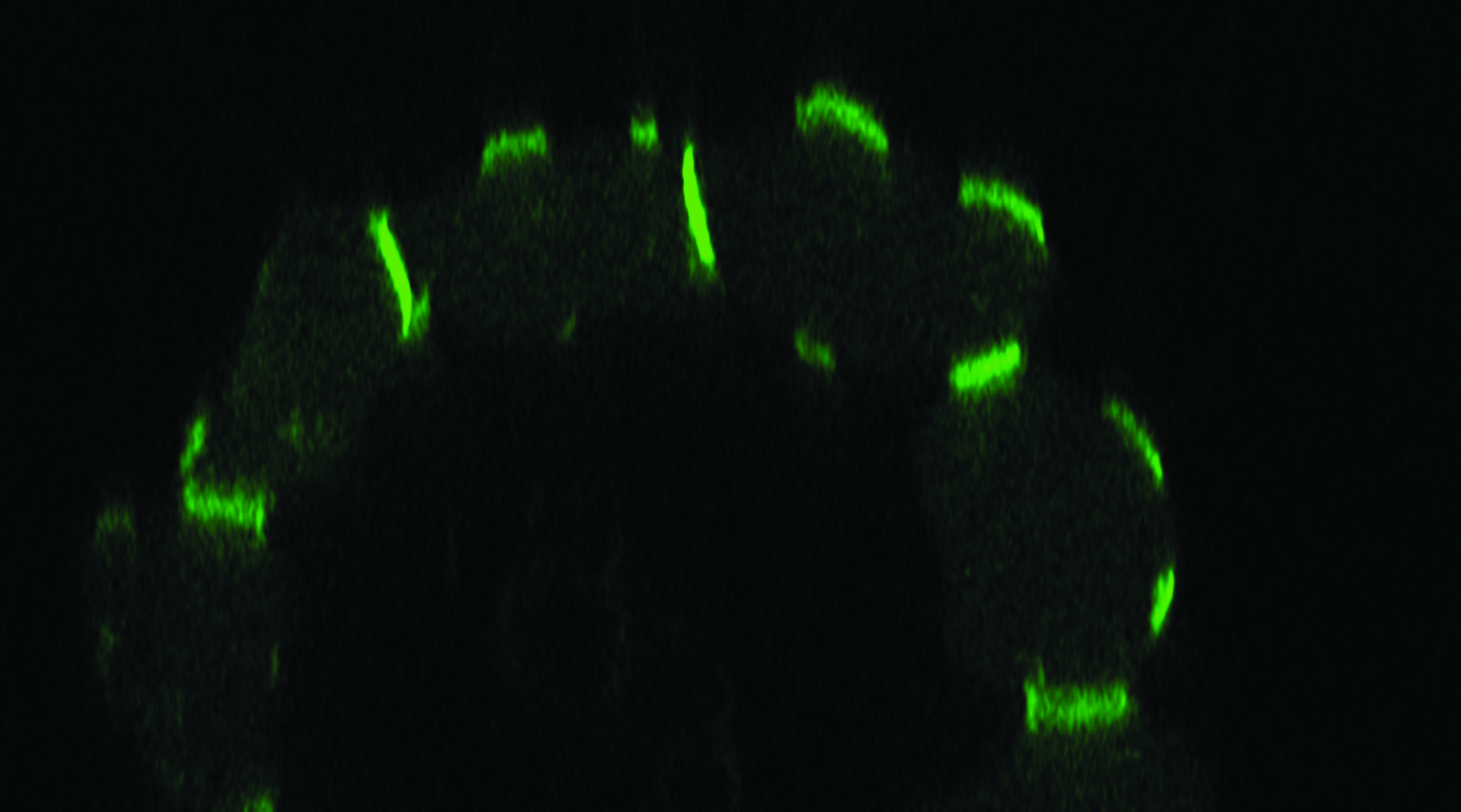

Supplement: Supplementary file 6 — Source data Fig. 2 [file 44318_2024_107_MOESM6_ESM.zip › Figure 2/Figure 2B/insert_MYB_CASP.tif]

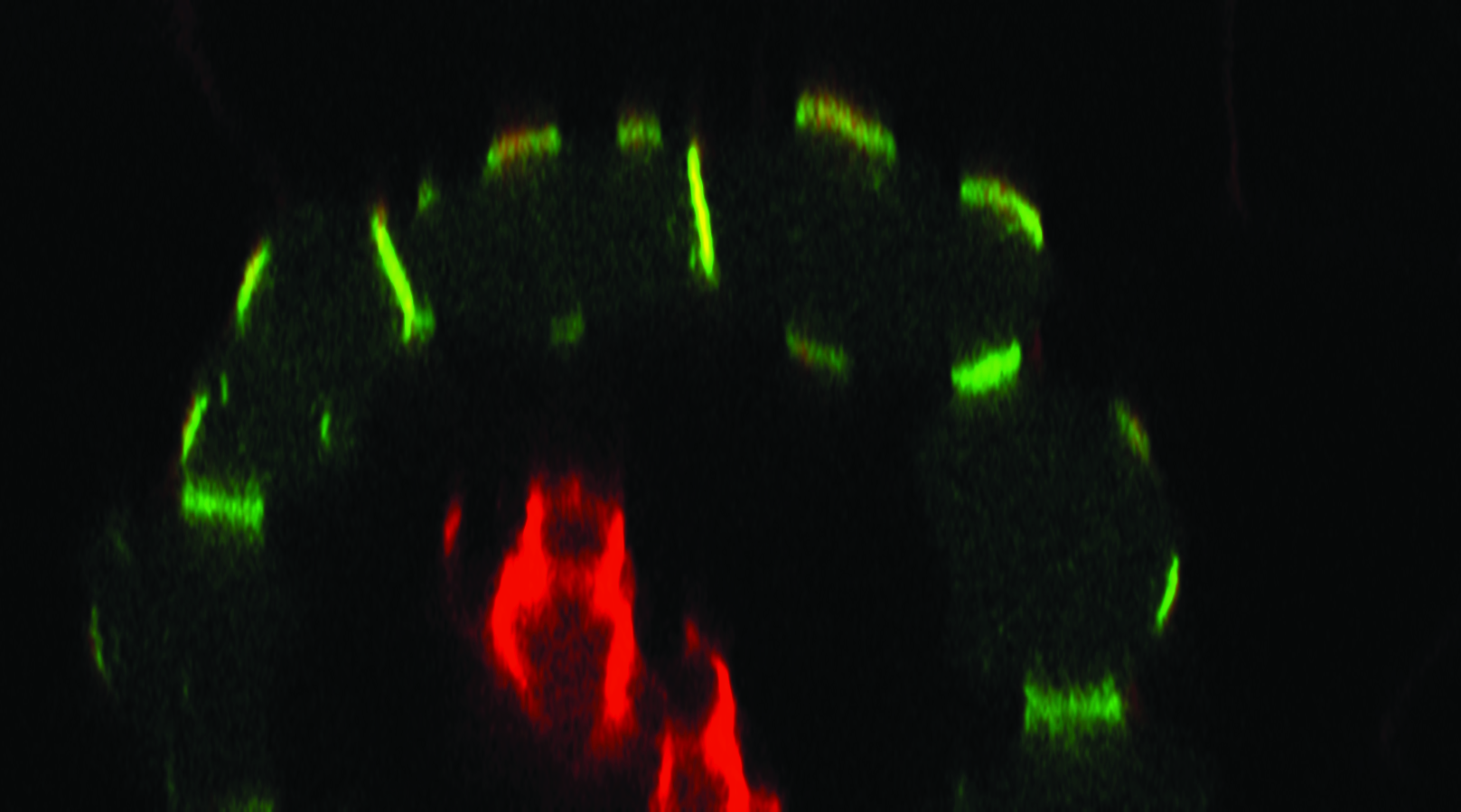

Supplement: Supplementary file 6 — Source data Fig. 2 [file 44318_2024_107_MOESM6_ESM.zip › Figure 2/Figure 2B/insert_MYB_overlay.tif]

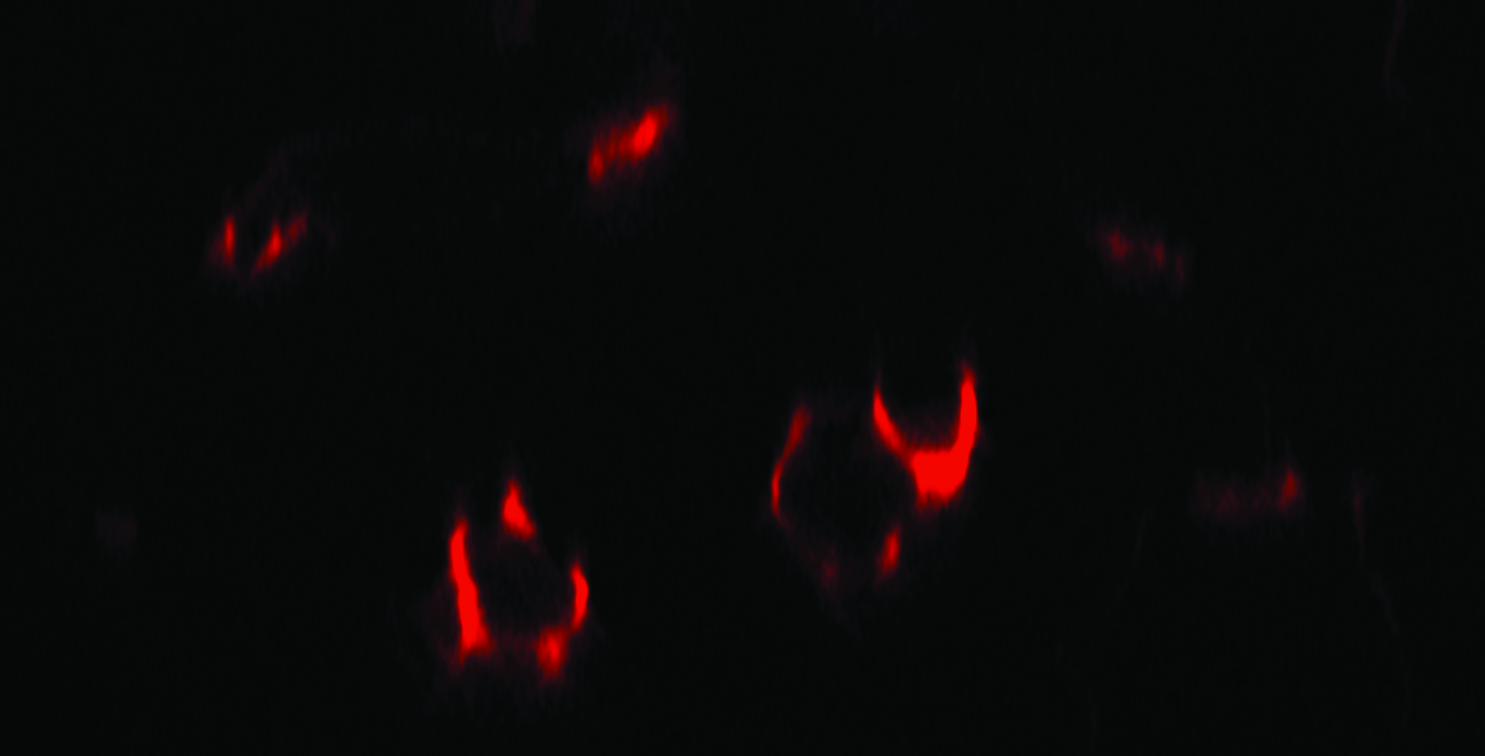

Supplement: Supplementary file 6 — Source data Fig. 2 [file 44318_2024_107_MOESM6_ESM.zip › Figure 2/Figure 2B/insert_WT_BF.tif]

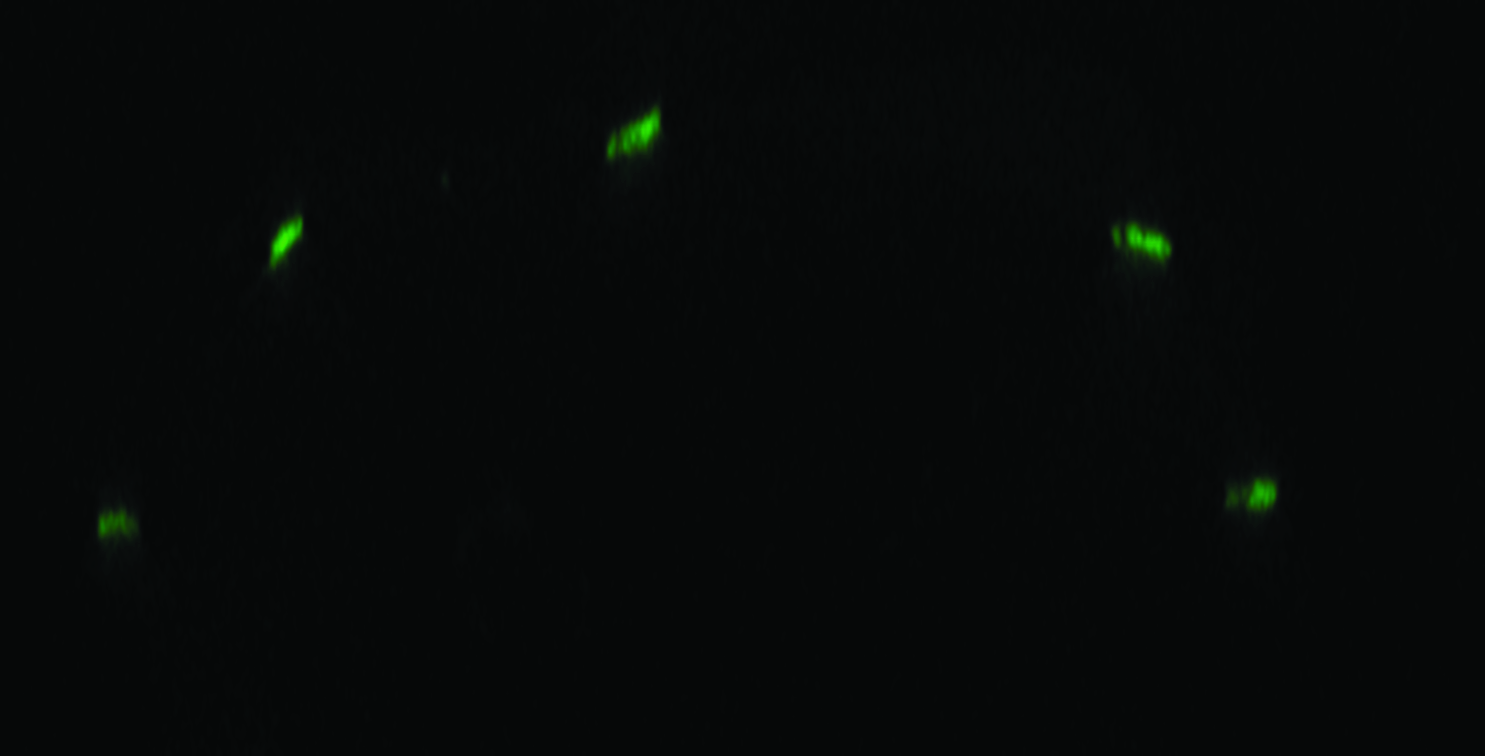

Supplement: Supplementary file 6 — Source data Fig. 2 [file 44318_2024_107_MOESM6_ESM.zip › Figure 2/Figure 2B/Insert_WT_CASP.tif]

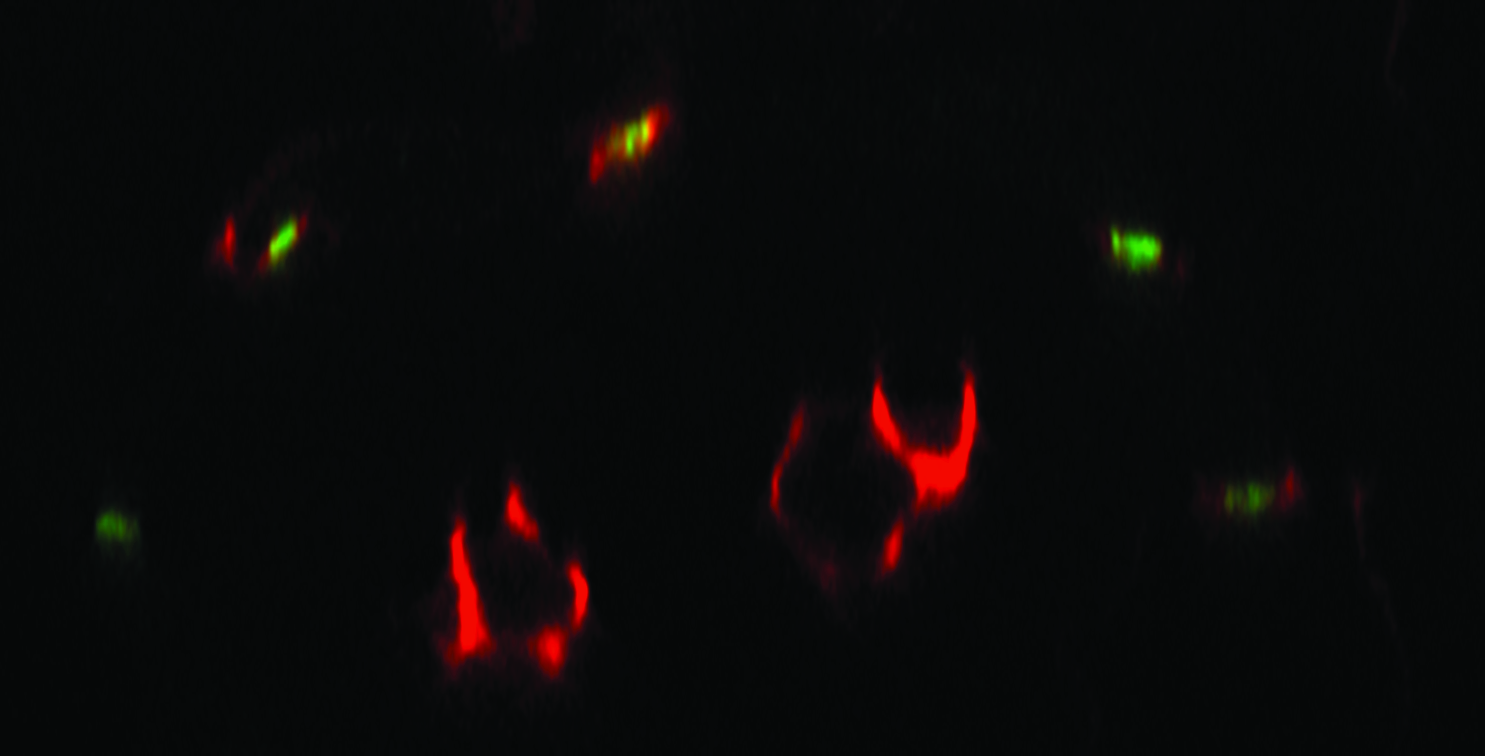

Supplement: Supplementary file 6 — Source data Fig. 2 [file 44318_2024_107_MOESM6_ESM.zip › Figure 2/Figure 2B/Insert_WT_overlay.tif]

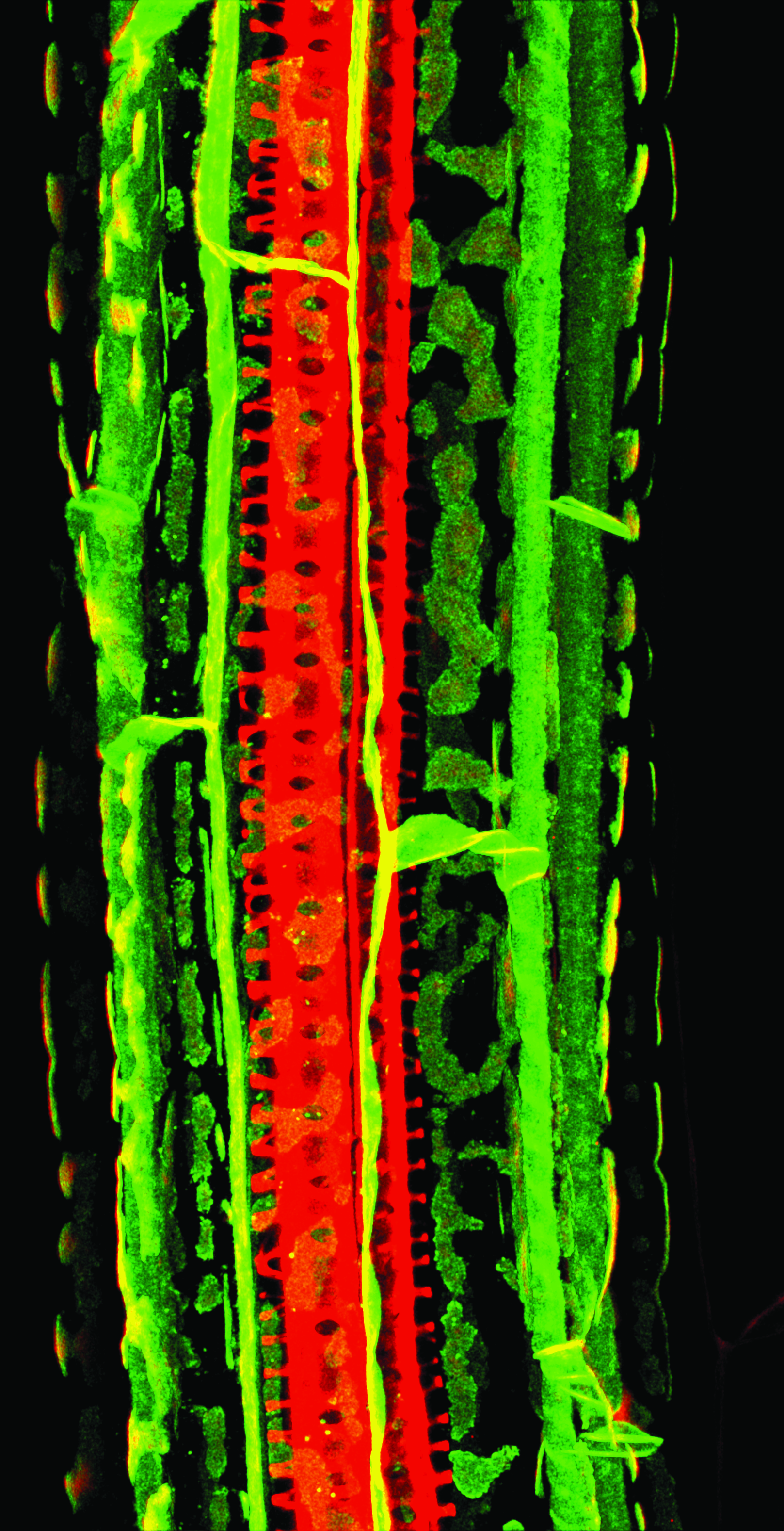

Supplement: Supplementary file 6 — Source data Fig. 2 [file 44318_2024_107_MOESM6_ESM.zip › Figure 2/Figure 2B/MYB_whole.tif]

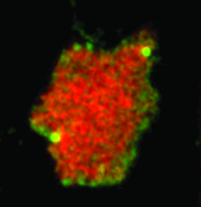

Supplement: Supplementary file 6 — Source data Fig. 2 [file 44318_2024_107_MOESM6_ESM.zip › Figure 2/Figure 2B/overlay.tif]

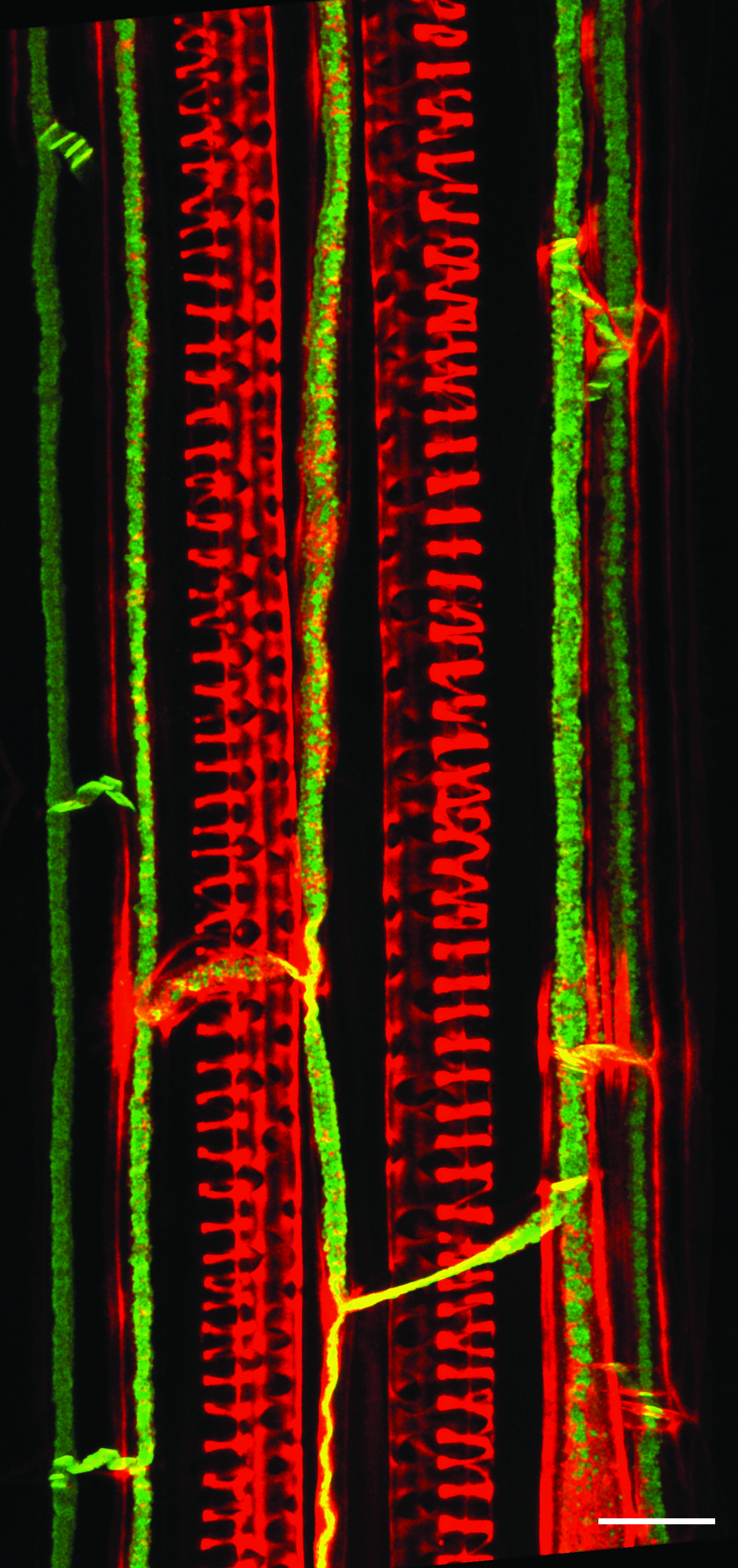

Supplement: Supplementary file 6 — Source data Fig. 2 [file 44318_2024_107_MOESM6_ESM.zip › Figure 2/Figure 2B/WT_whole.tif]

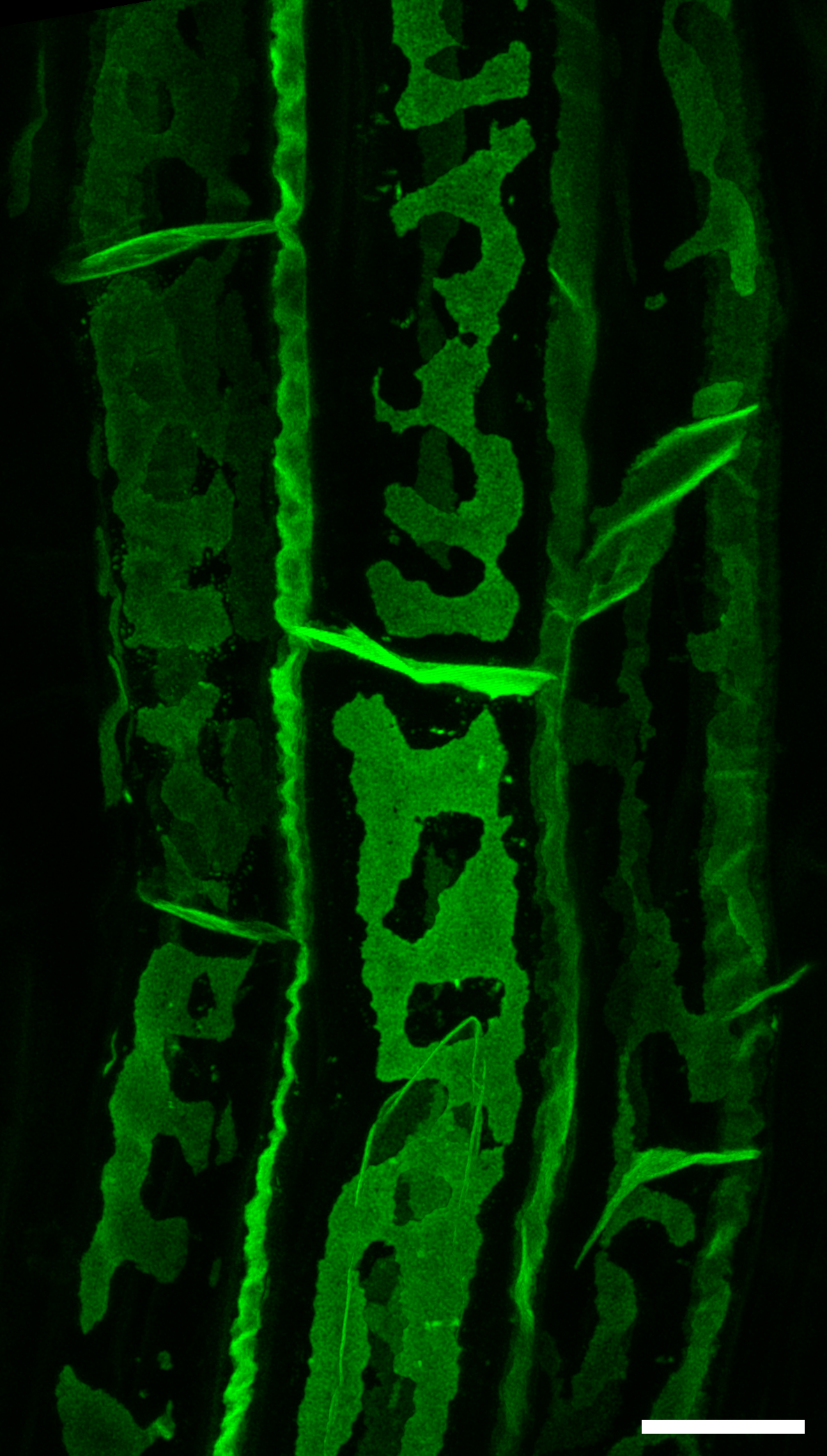

Supplement: Supplementary file 6 — Source data Fig. 2 [file 44318_2024_107_MOESM6_ESM.zip › Figure 2/Figure 2C/C1-MAX_C36 ESB1_meta-1-2.tif]

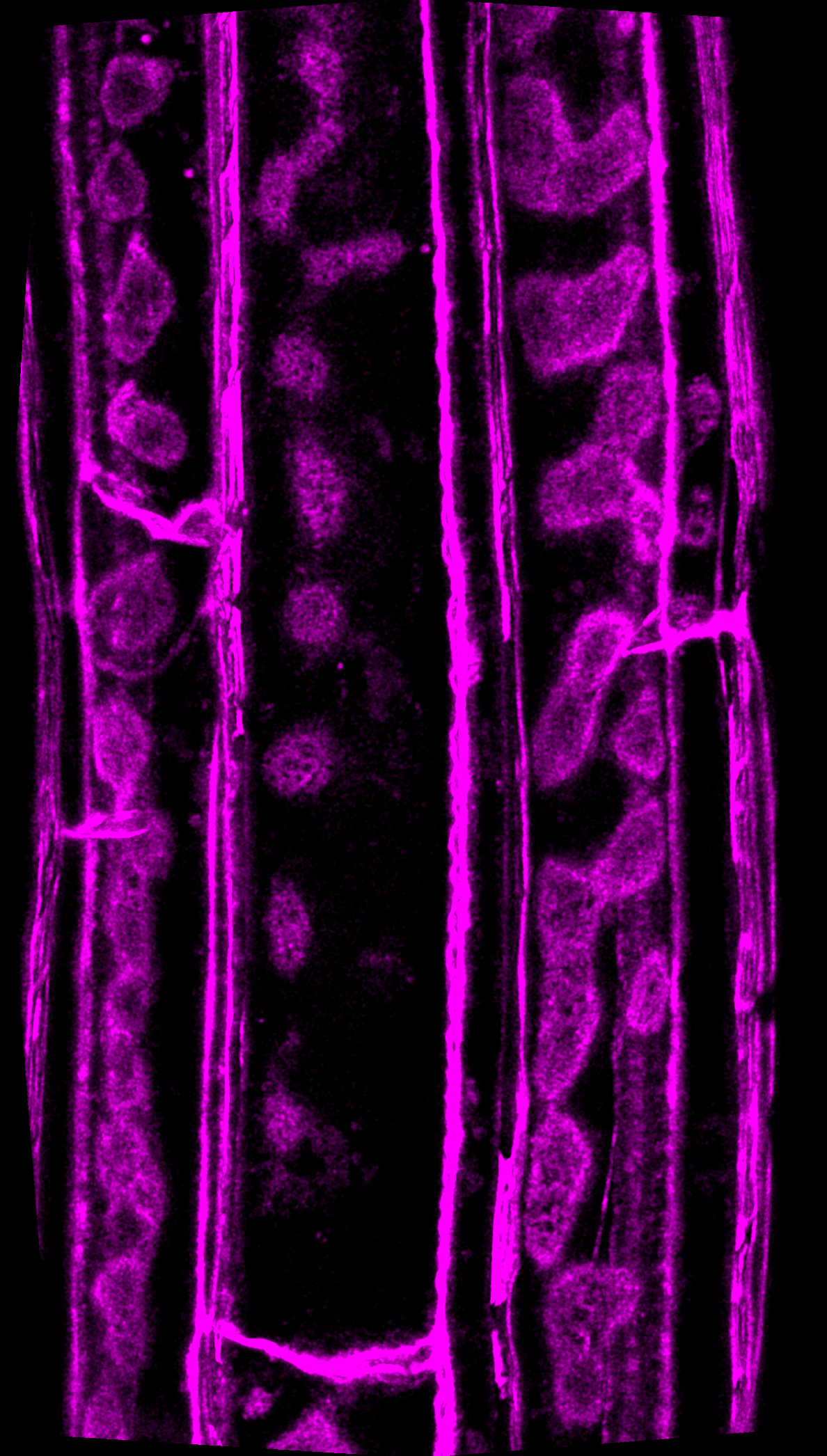

Supplement: Supplementary file 6 — Source data Fig. 2 [file 44318_2024_107_MOESM6_ESM.zip › Figure 2/Figure 2C/C1-MAX_C36 PER64_meta-1.tif]

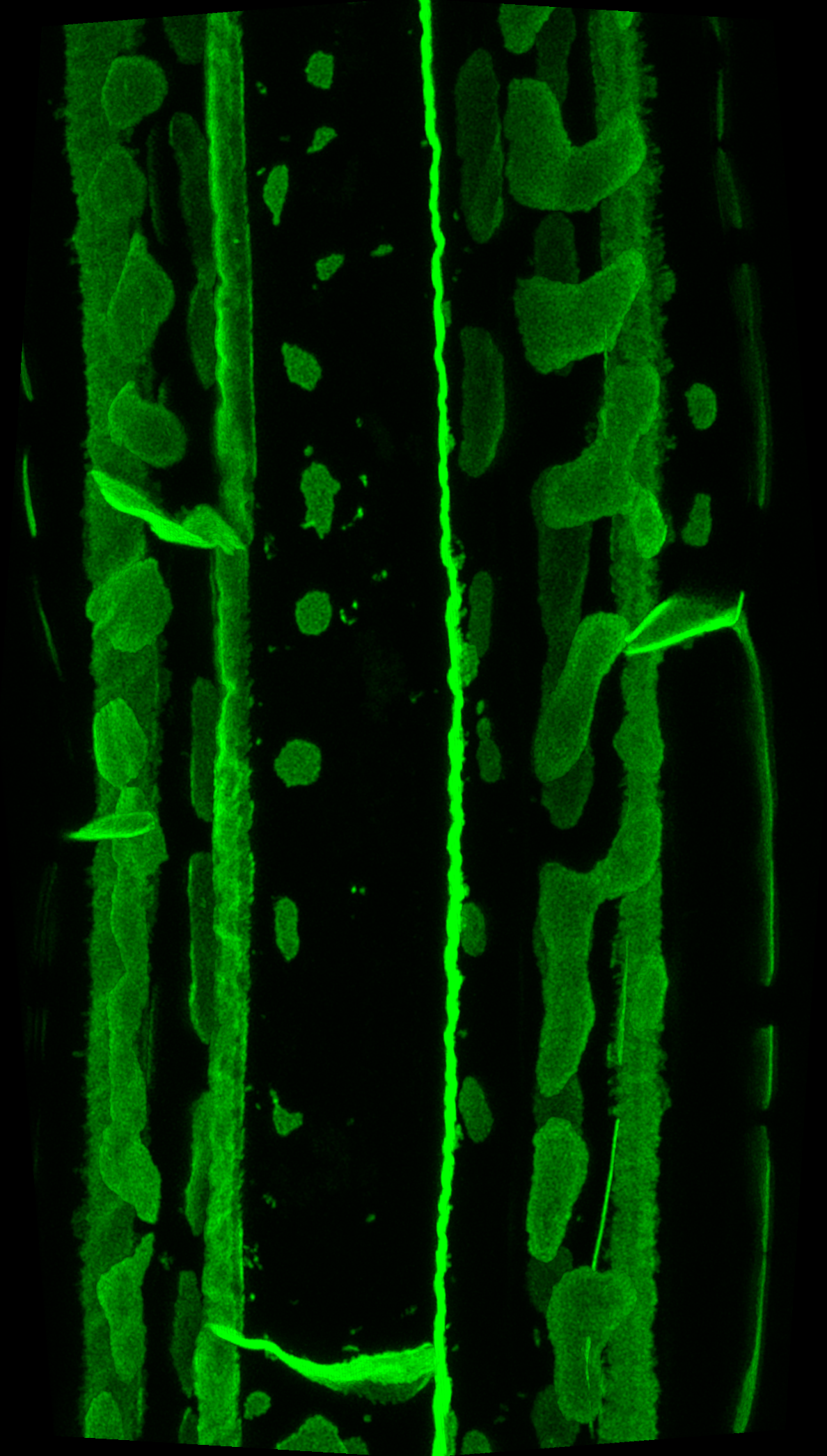

Supplement: Supplementary file 6 — Source data Fig. 2 [file 44318_2024_107_MOESM6_ESM.zip › Figure 2/Figure 2C/C2-MAX_C36 PER64_meta-1.tif]

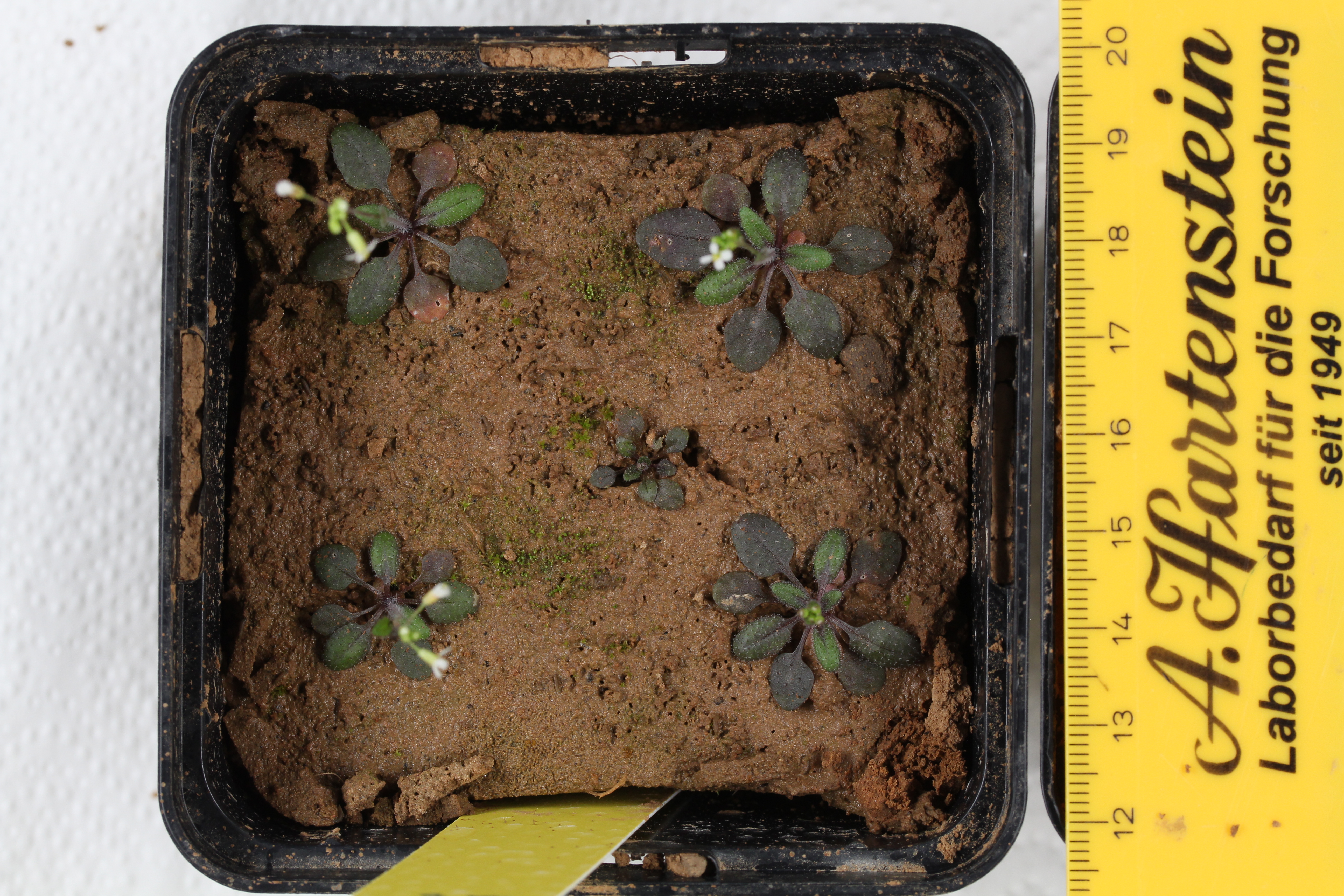

Supplement: Supplementary file 8 — Source data Fig. 4 [file 44318_2024_107_MOESM8_ESM.zip › Figure 4/Figure 4A/Col-0 CAS.JPG]

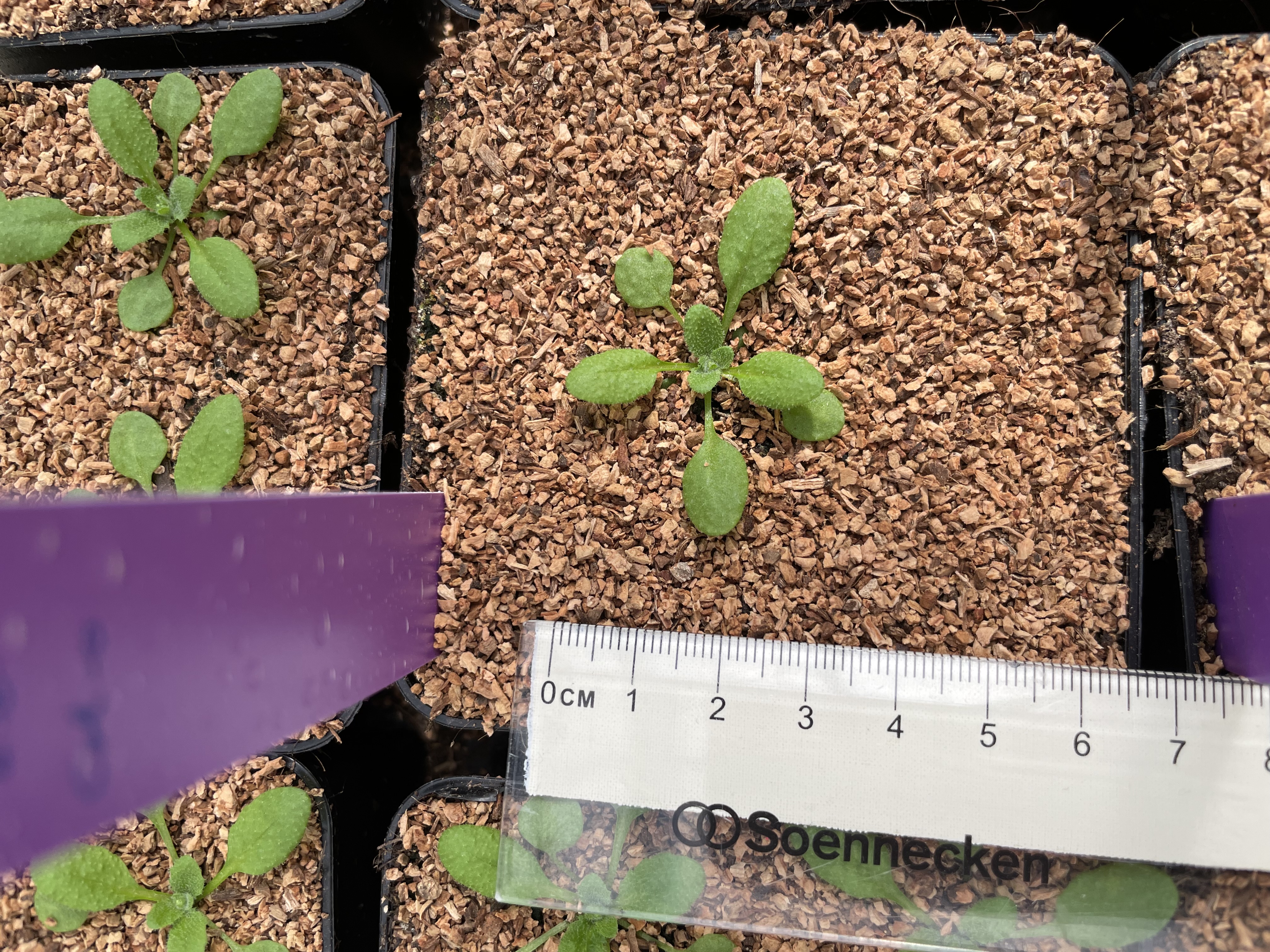

Supplement: Supplementary file 8 — Source data Fig. 4 [file 44318_2024_107_MOESM8_ESM.zip › Figure 4/Figure 4A/Col-0 Potting.jpg]

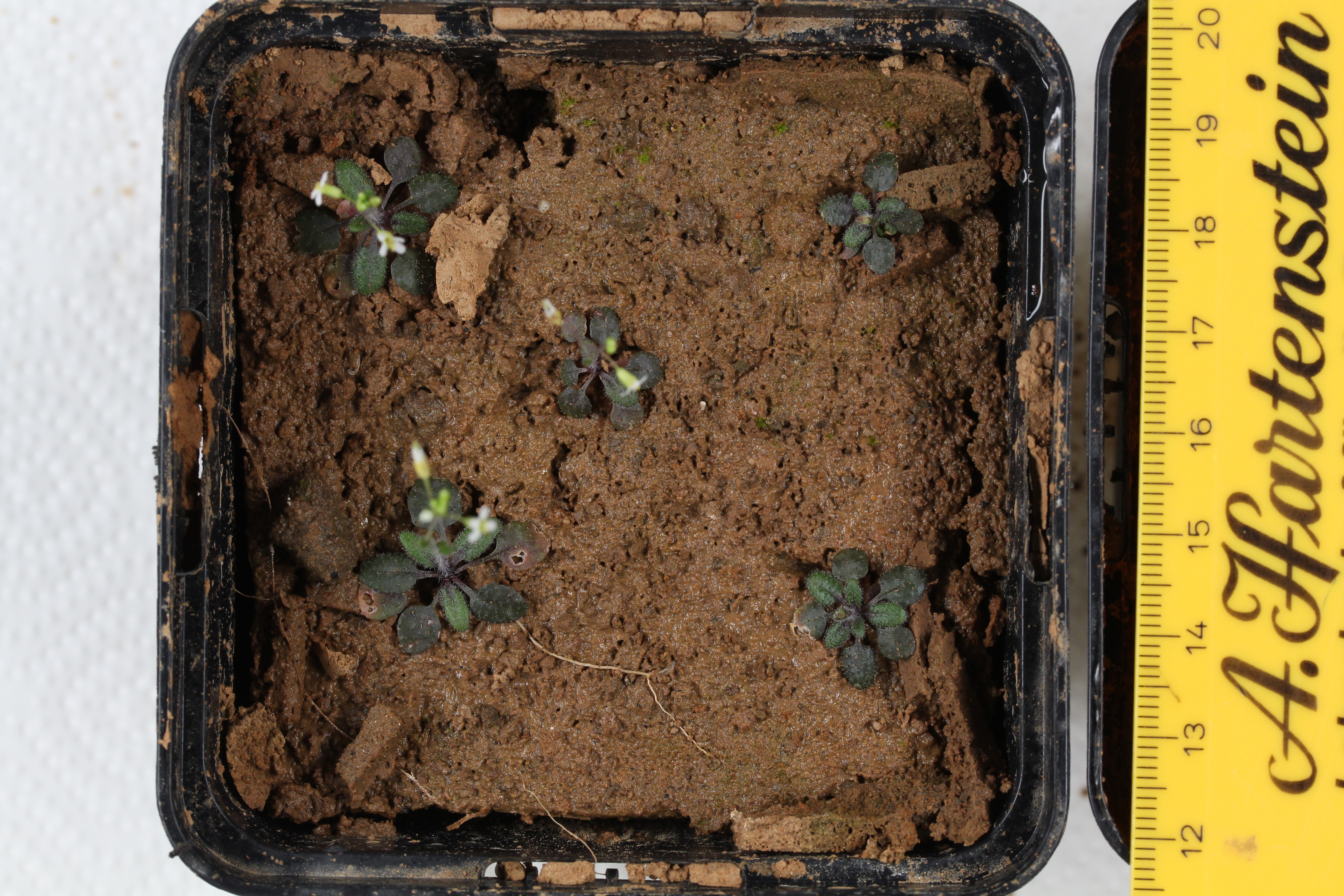

Supplement: Supplementary file 8 — Source data Fig. 4 [file 44318_2024_107_MOESM8_ESM.zip › Figure 4/Figure 4A/myb36-2 CAS.JPG]

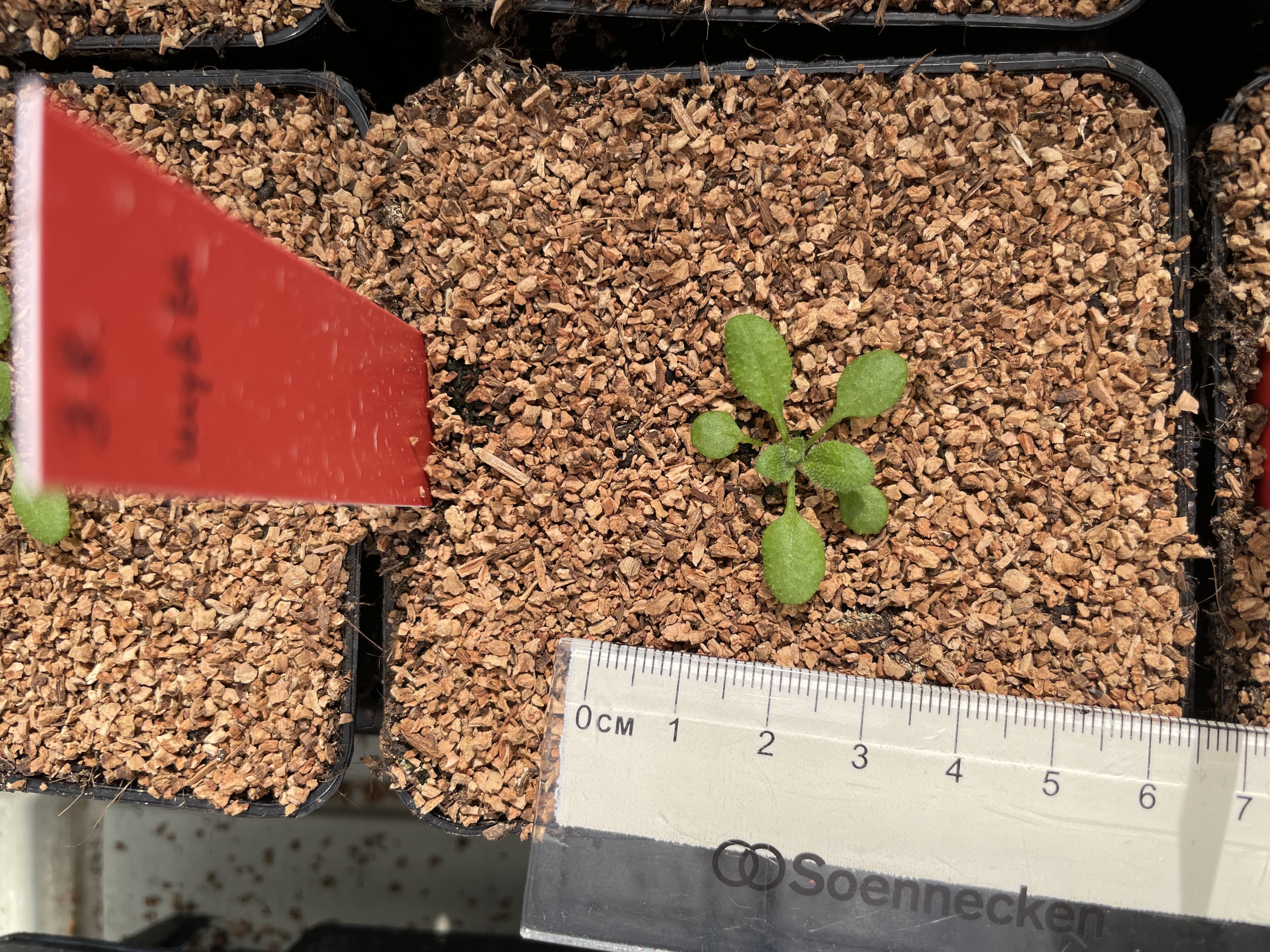

Supplement: Supplementary file 8 — Source data Fig. 4 [file 44318_2024_107_MOESM8_ESM.zip › Figure 4/Figure 4A/myb36-2 Potting.jpg]

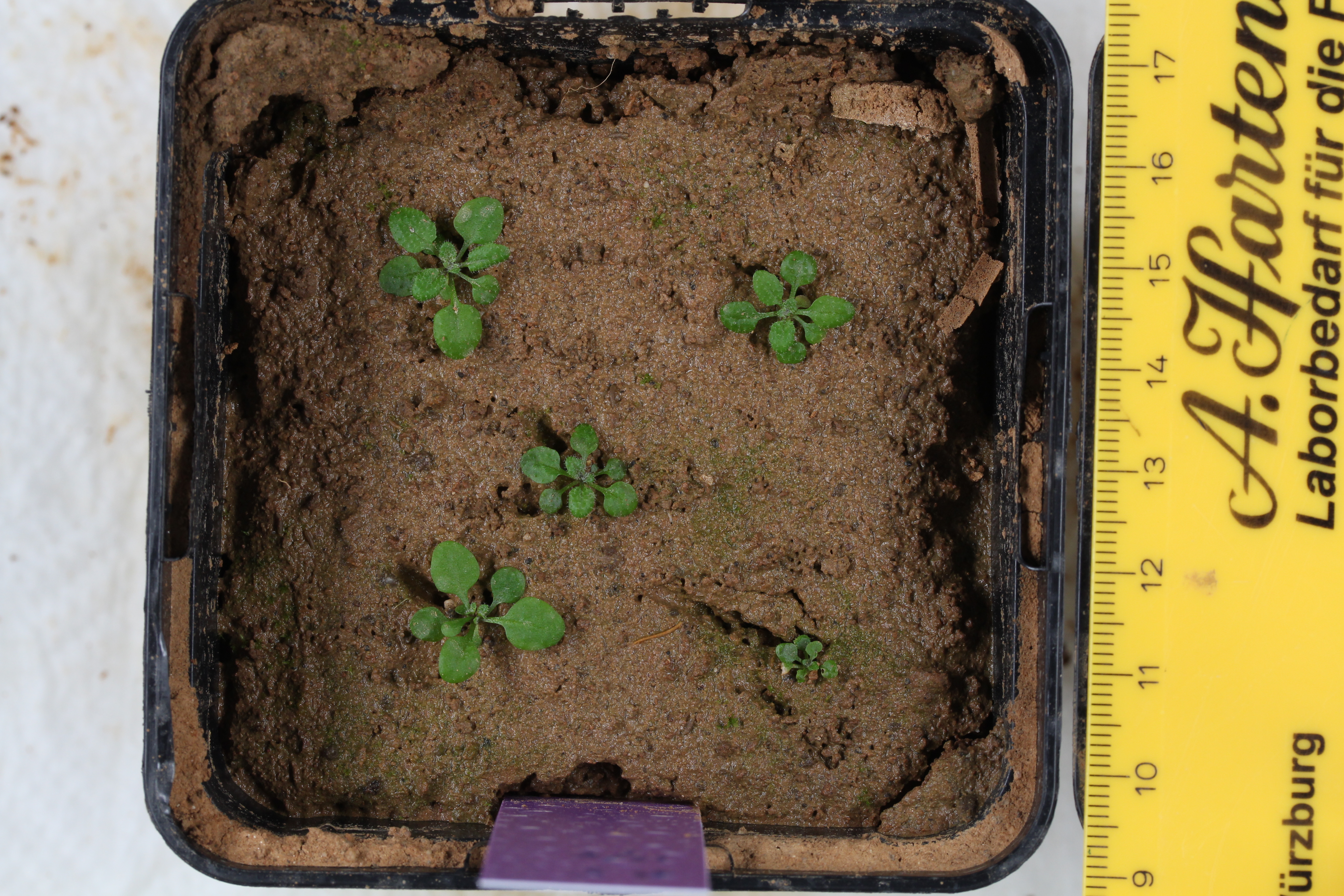

Supplement: Supplementary file 8 — Source data Fig. 4 [file 44318_2024_107_MOESM8_ESM.zip › Figure 4/Figure 4A/MYB36Loop#5 CAS.JPG]

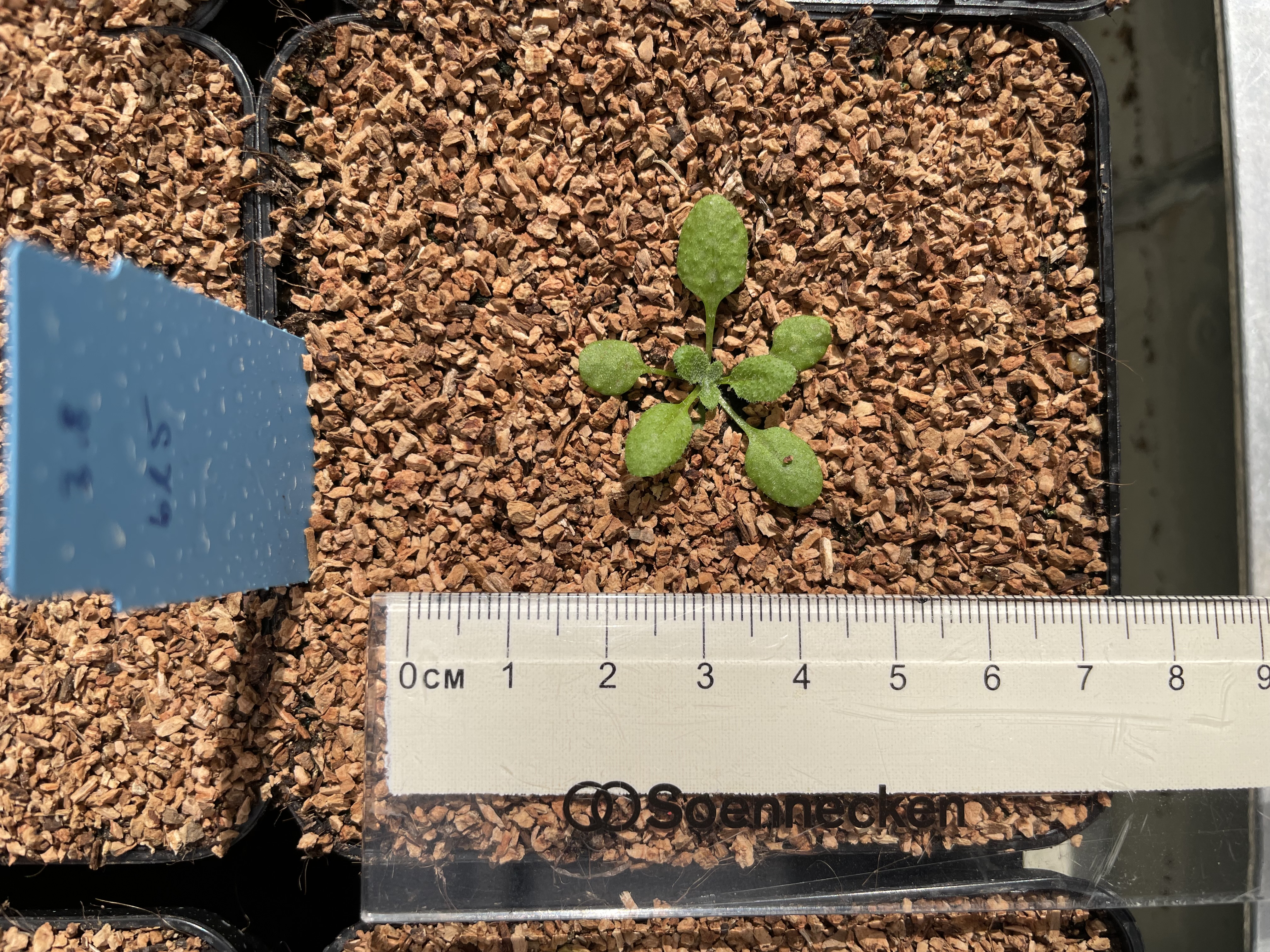

Supplement: Supplementary file 8 — Source data Fig. 4 [file 44318_2024_107_MOESM8_ESM.zip › Figure 4/Figure 4A/MYB36Loop#5 Potting.jpg]

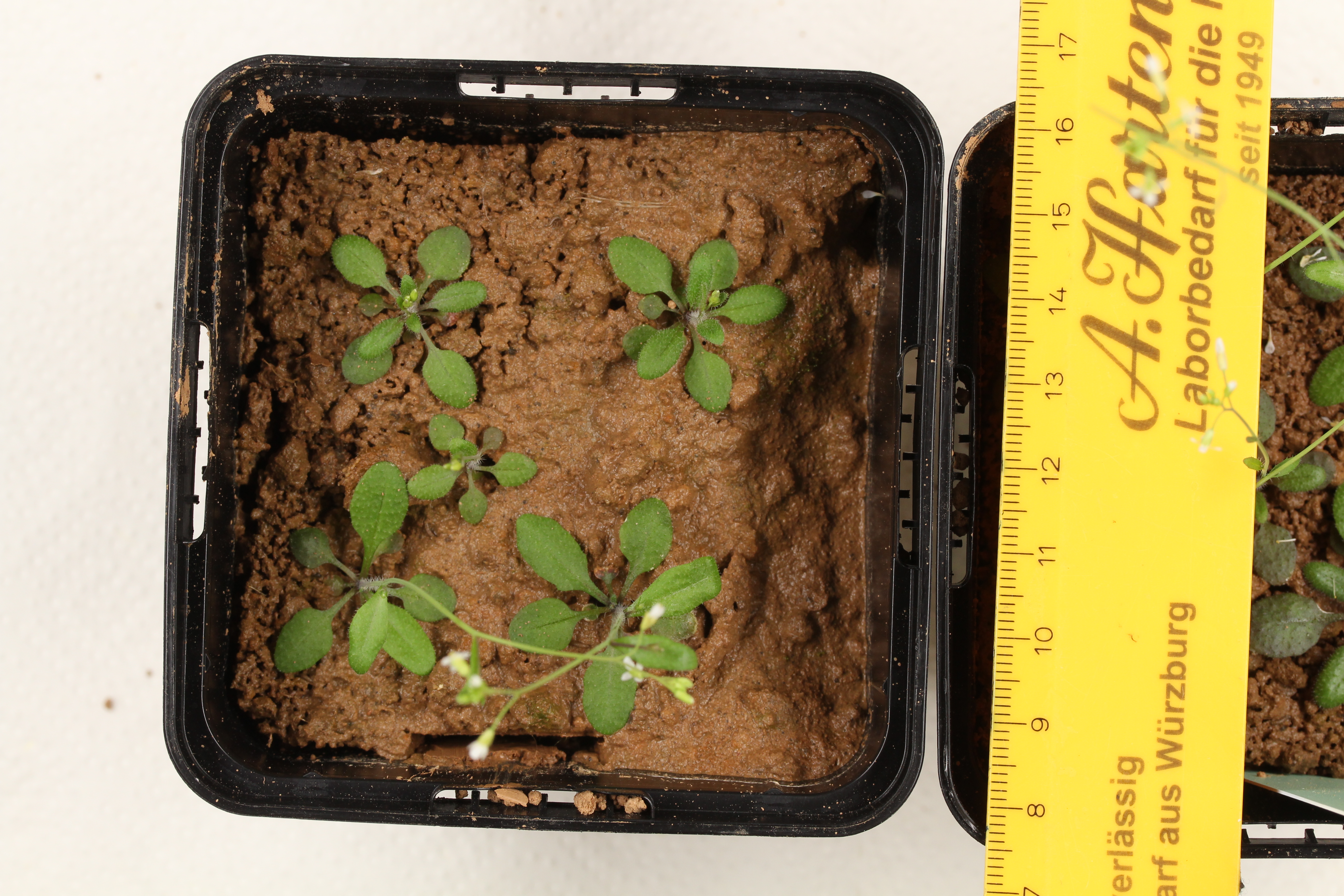

Supplement: Supplementary file 8 — Source data Fig. 4 [file 44318_2024_107_MOESM8_ESM.zip › Figure 4/Figure 4A/sgn3-3 CAS.JPG]

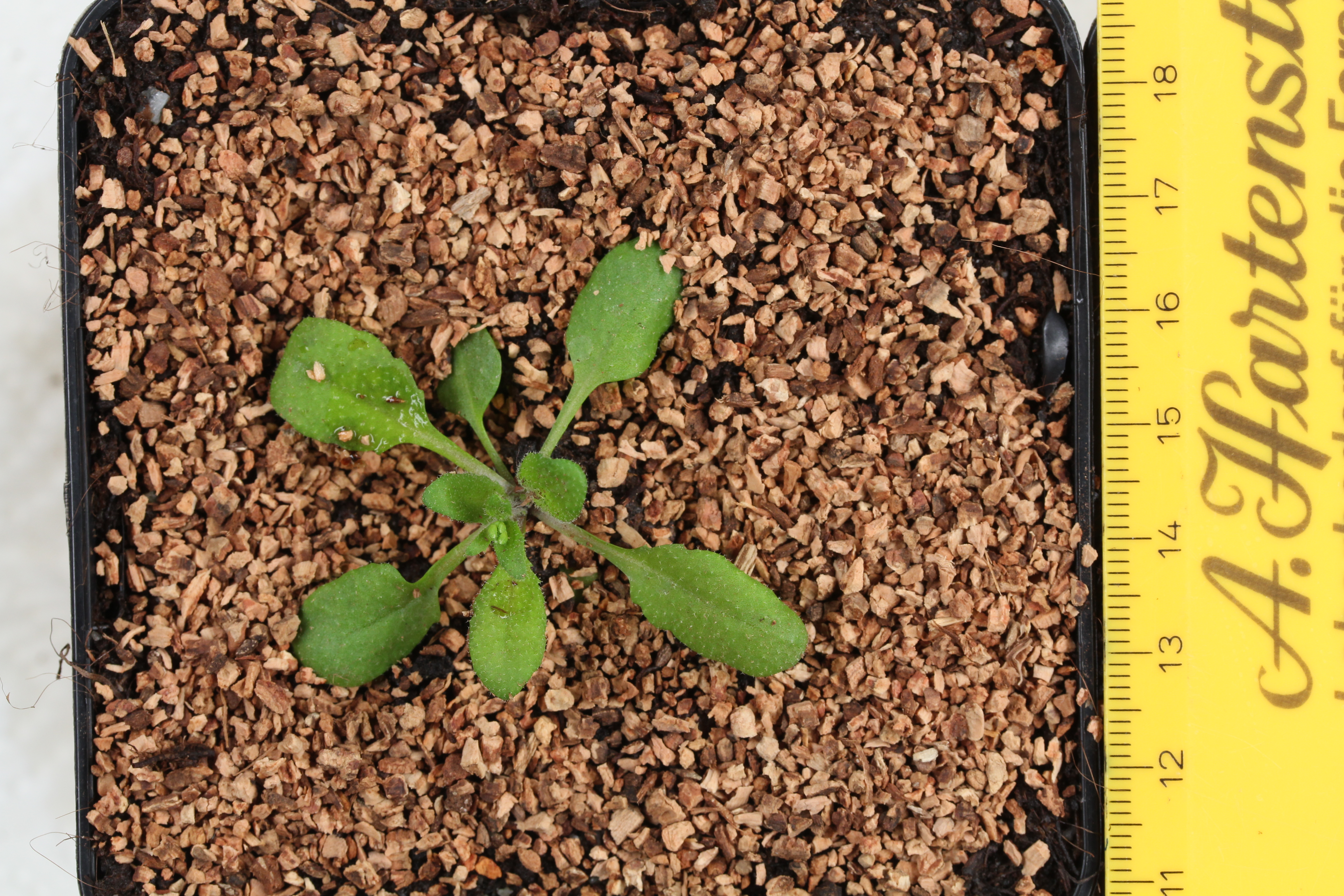

Supplement: Supplementary file 8 — Source data Fig. 4 [file 44318_2024_107_MOESM8_ESM.zip › Figure 4/Figure 4A/sgn3-3 Potting.JPG]

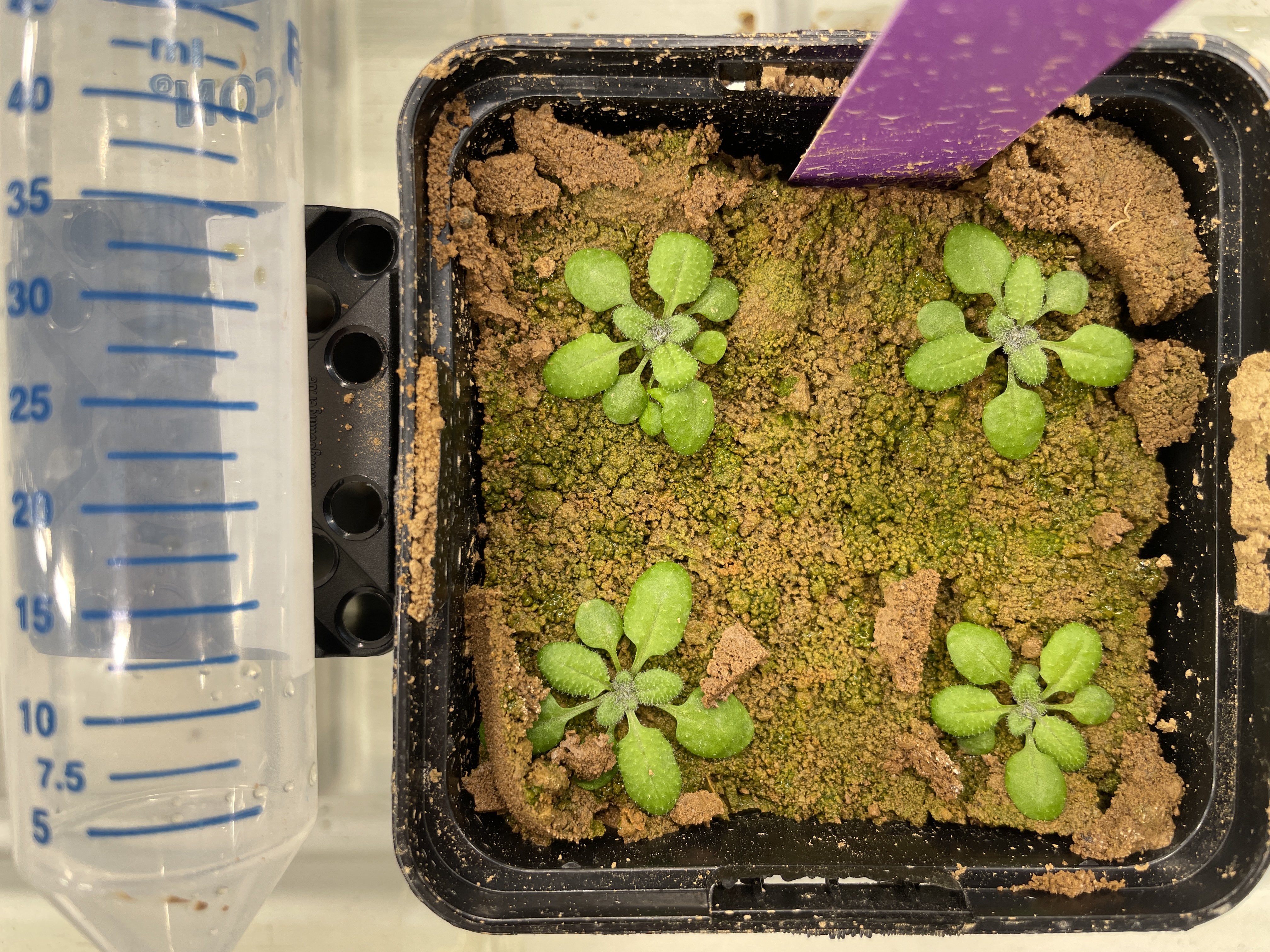

Supplement: Supplementary file 9 — Source data Fig. 5 [file 44318_2024_107_MOESM9_ESM.zip › Figure 5/Figure 5A/Calcinit/BL5.JPG]

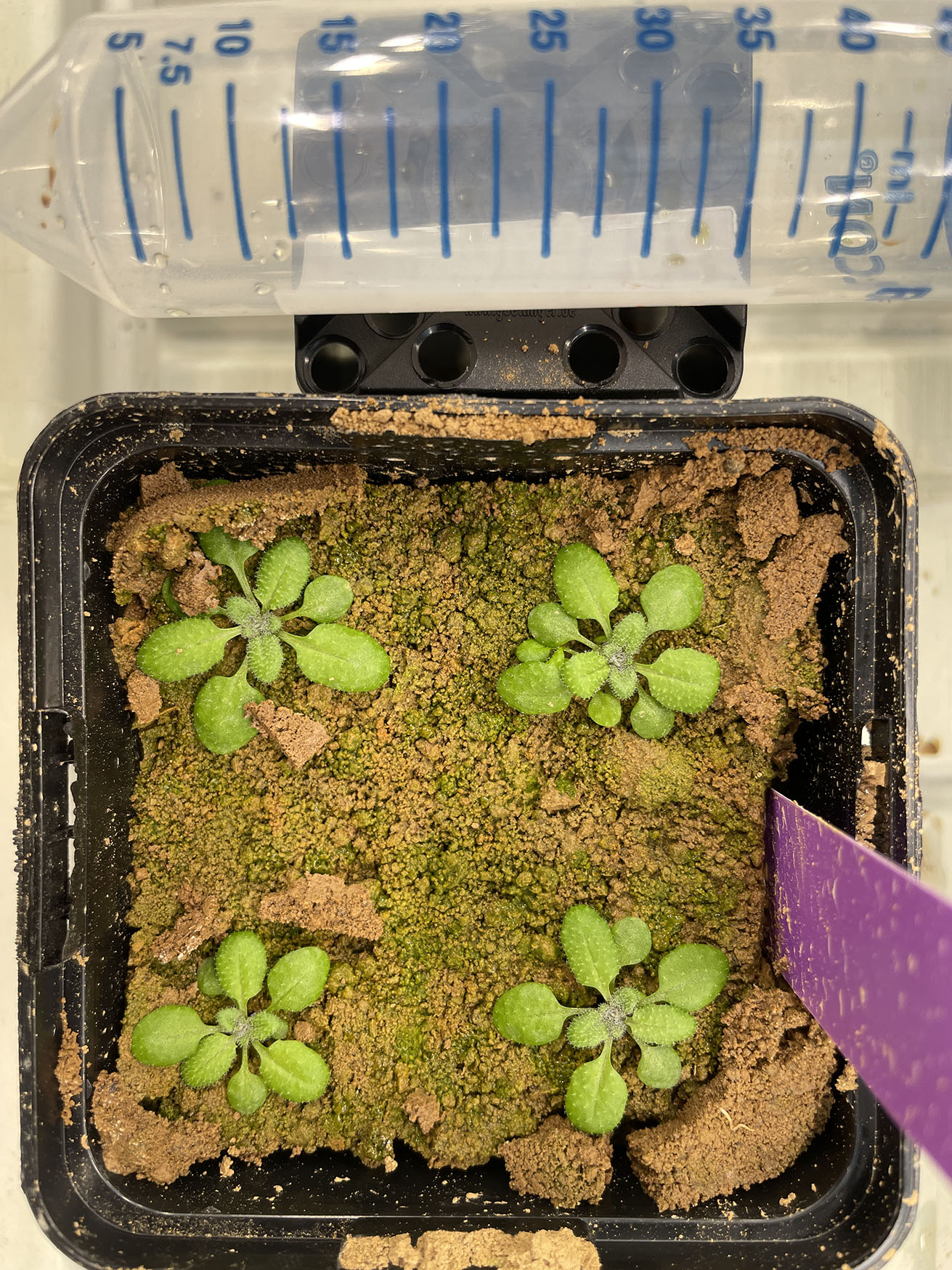

Supplement: Supplementary file 9 — Source data Fig. 5 [file 44318_2024_107_MOESM9_ESM.zip › Figure 5/Figure 5A/Calcinit/BL5_small.jpg]

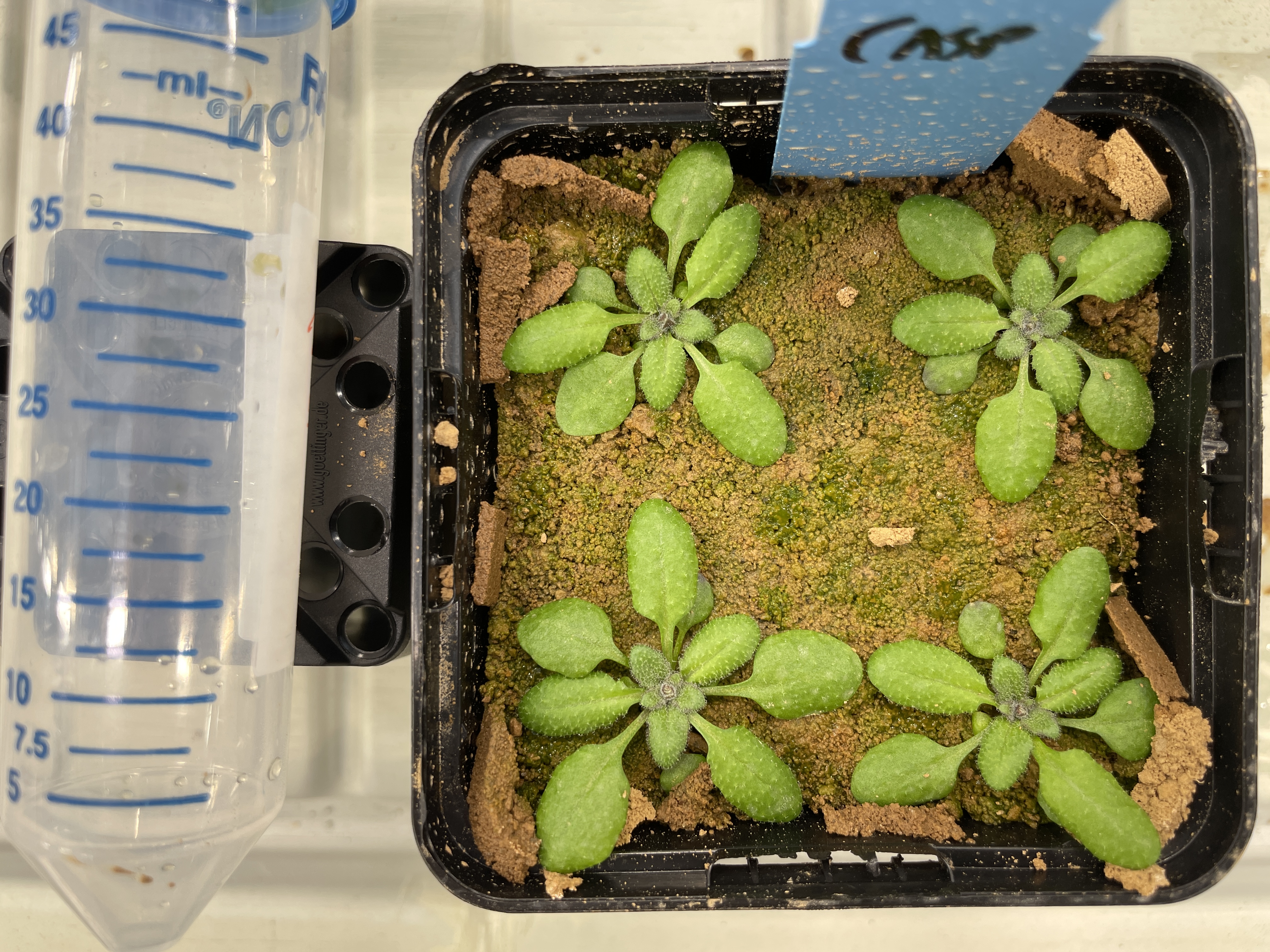

Supplement: Supplementary file 9 — Source data Fig. 5 [file 44318_2024_107_MOESM9_ESM.zip › Figure 5/Figure 5A/Calcinit/CASP.JPG]

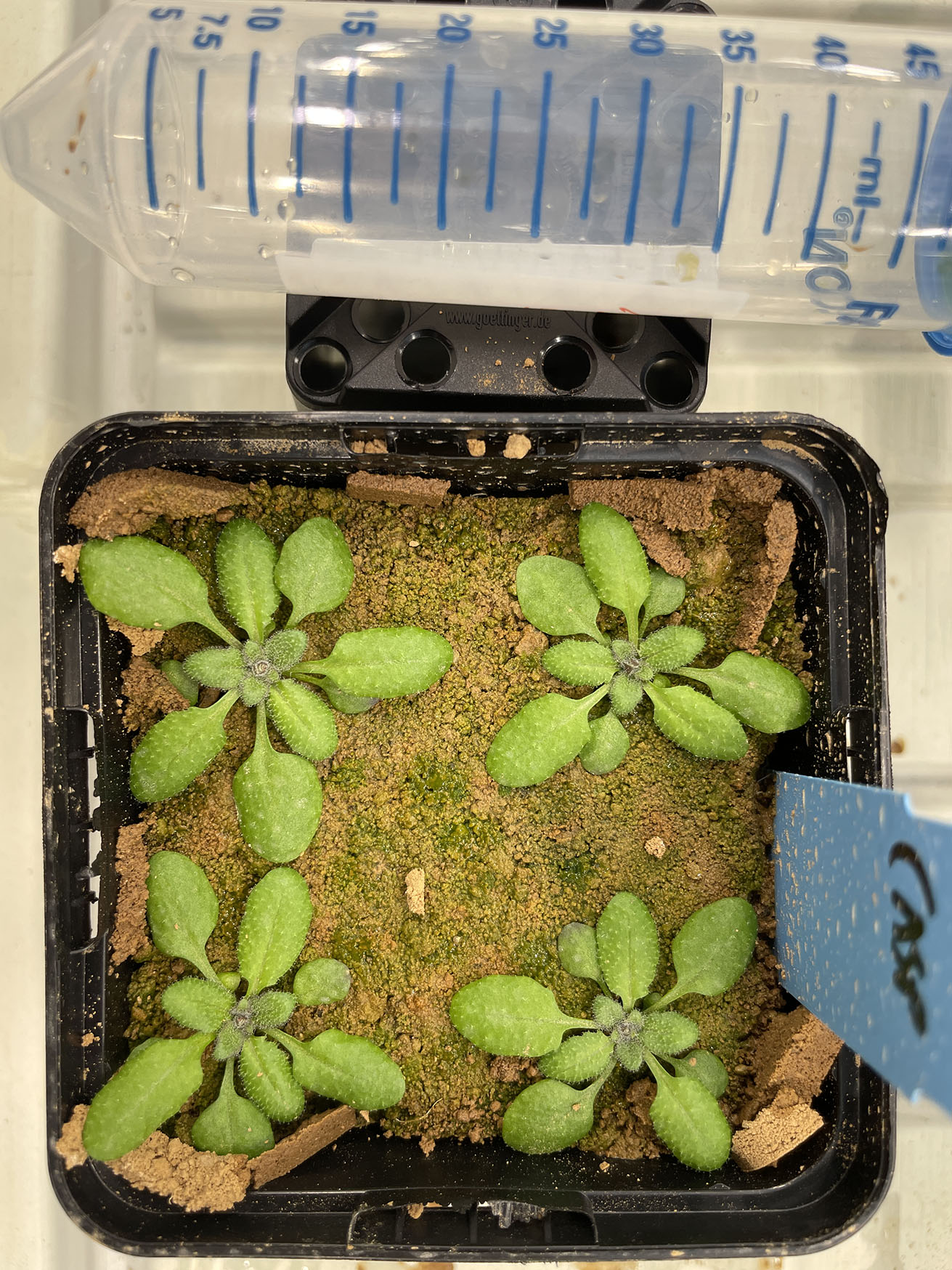

Supplement: Supplementary file 9 — Source data Fig. 5 [file 44318_2024_107_MOESM9_ESM.zip › Figure 5/Figure 5A/Calcinit/CASP_small.jpg]

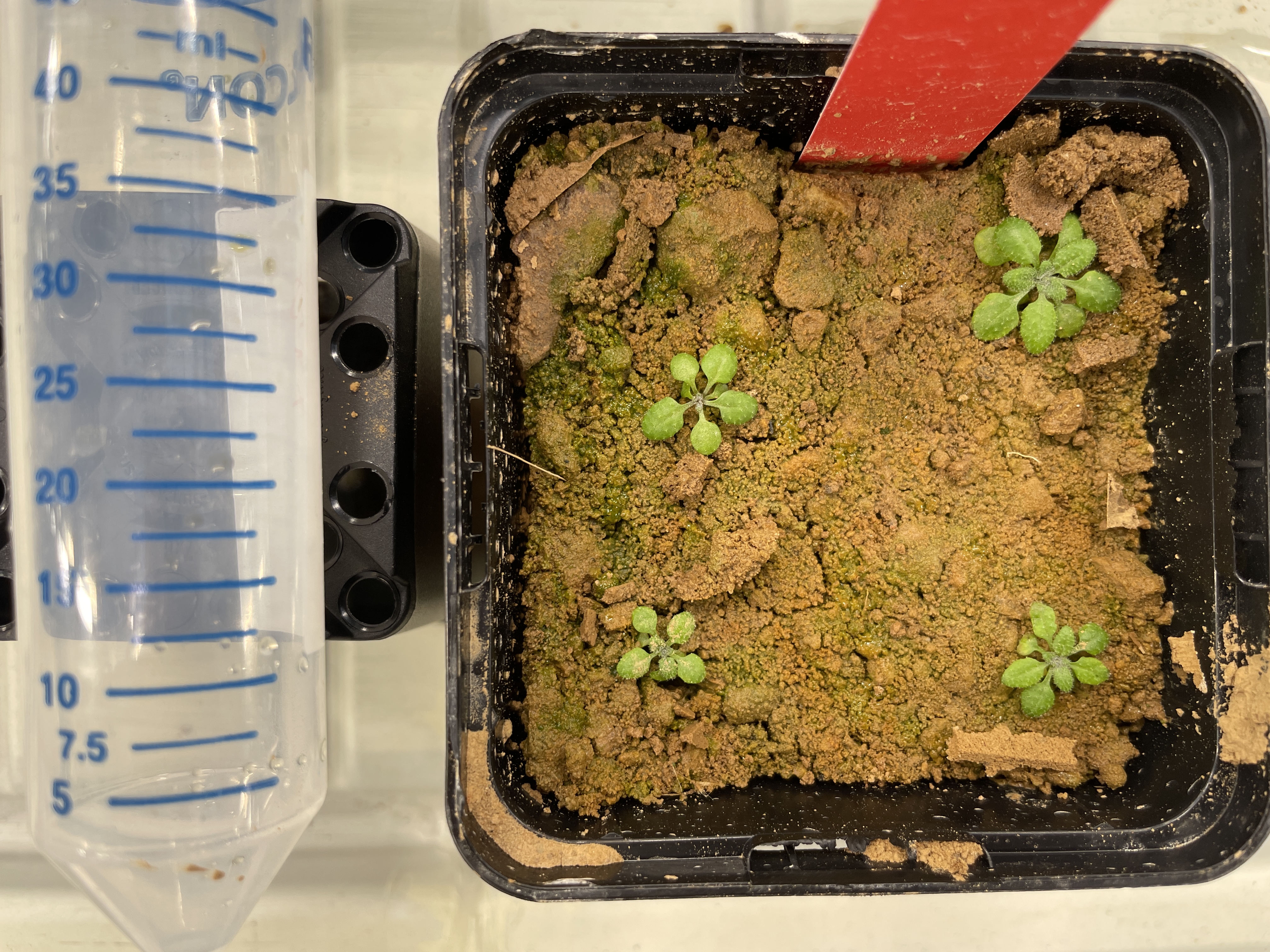

Supplement: Supplementary file 9 — Source data Fig. 5 [file 44318_2024_107_MOESM9_ESM.zip › Figure 5/Figure 5A/Calcinit/CIF.JPG]

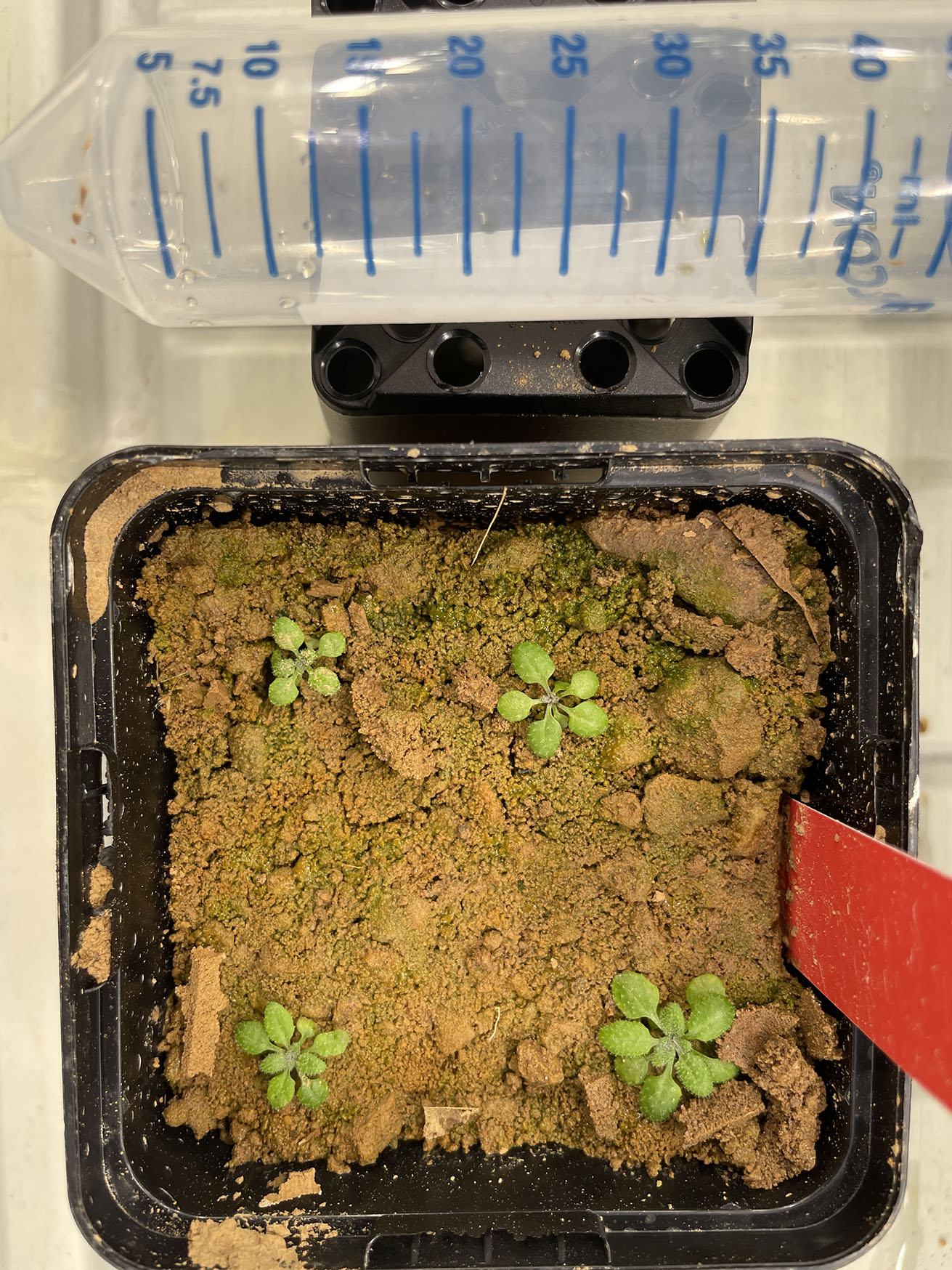

Supplement: Supplementary file 9 — Source data Fig. 5 [file 44318_2024_107_MOESM9_ESM.zip › Figure 5/Figure 5A/Calcinit/CIF_small.jpg]

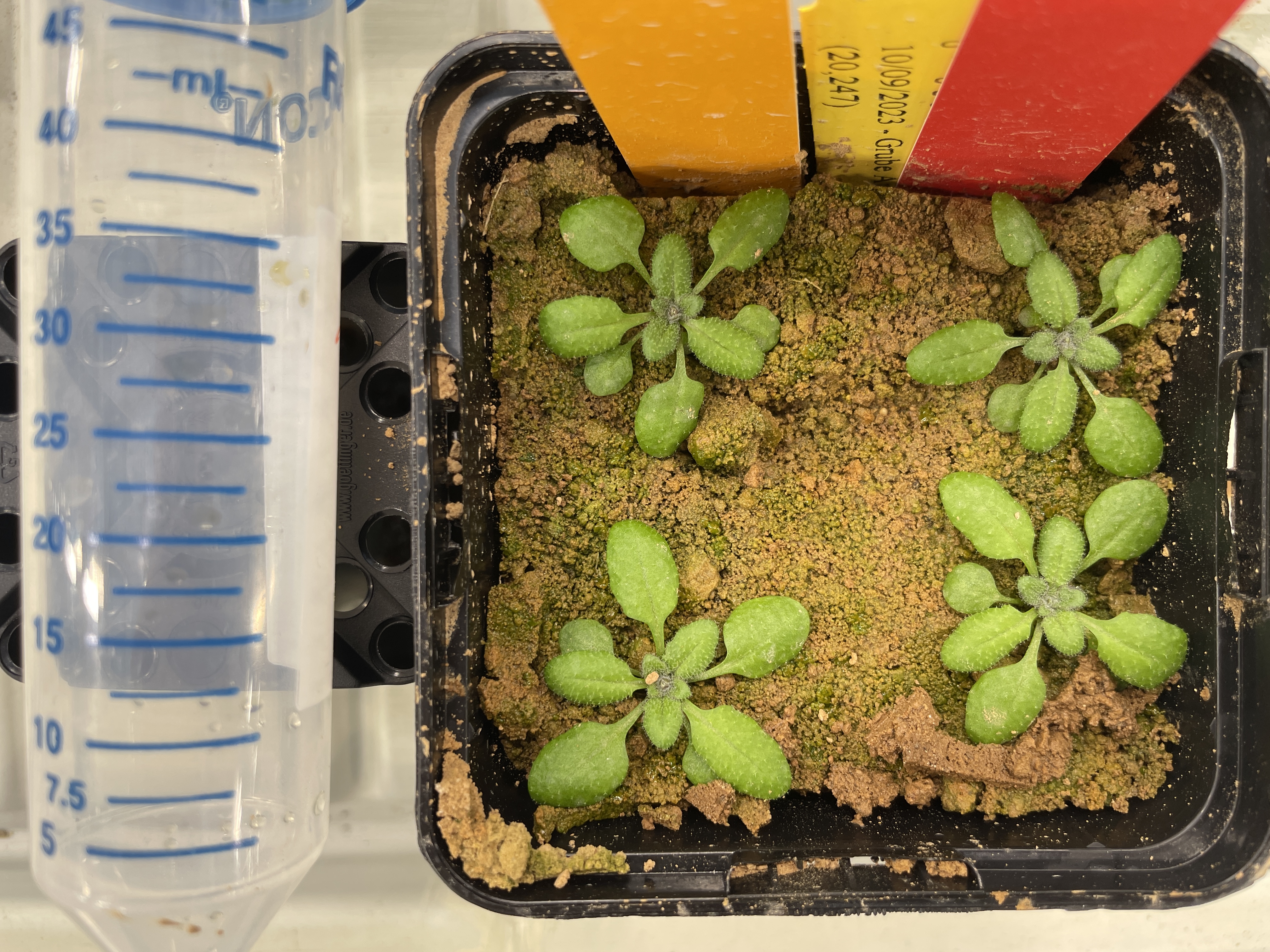

Supplement: Supplementary file 9 — Source data Fig. 5 [file 44318_2024_107_MOESM9_ESM.zip › Figure 5/Figure 5A/Calcinit/Col.JPG]

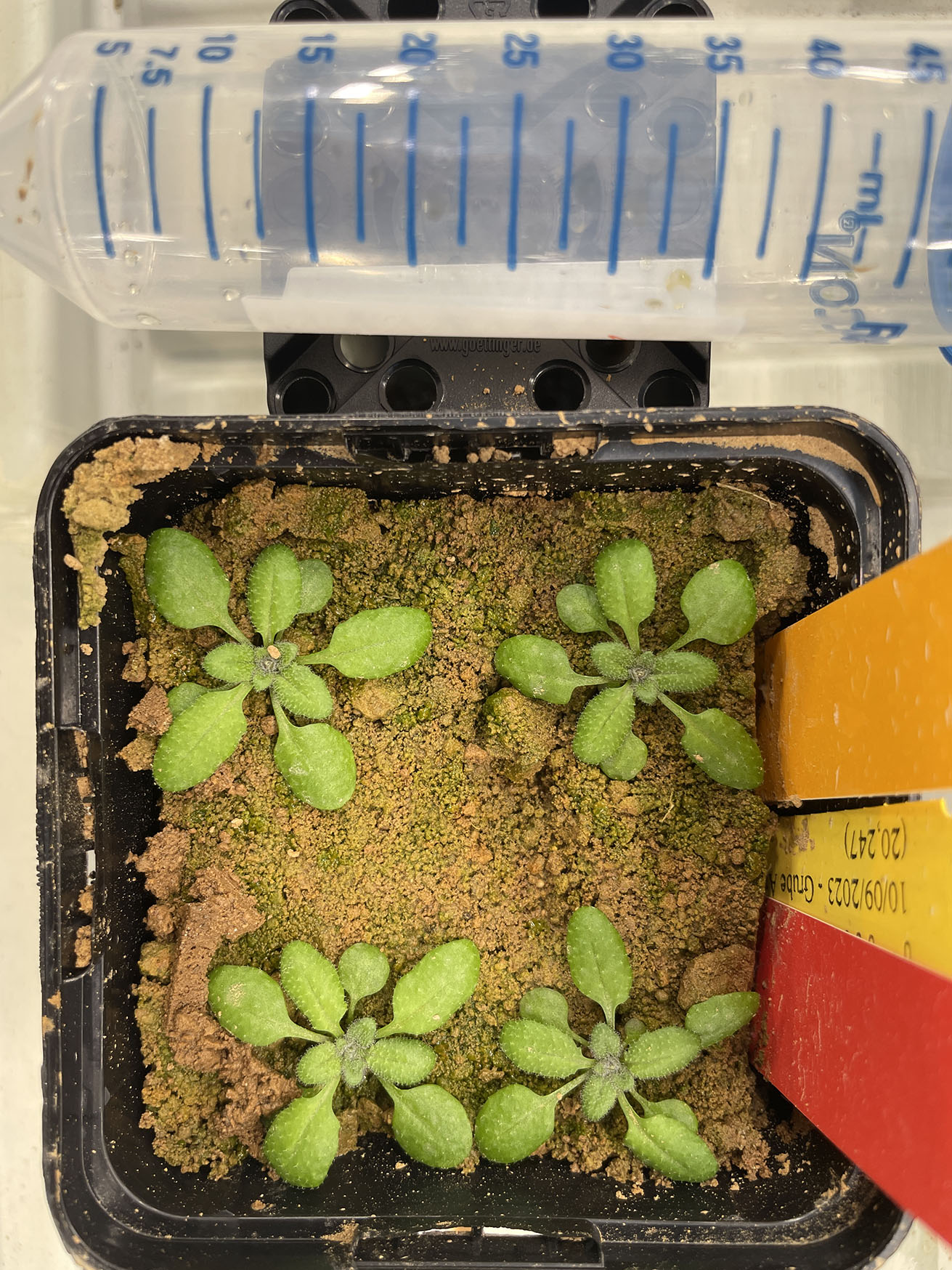

Supplement: Supplementary file 9 — Source data Fig. 5 [file 44318_2024_107_MOESM9_ESM.zip › Figure 5/Figure 5A/Calcinit/Col_small.jpg]

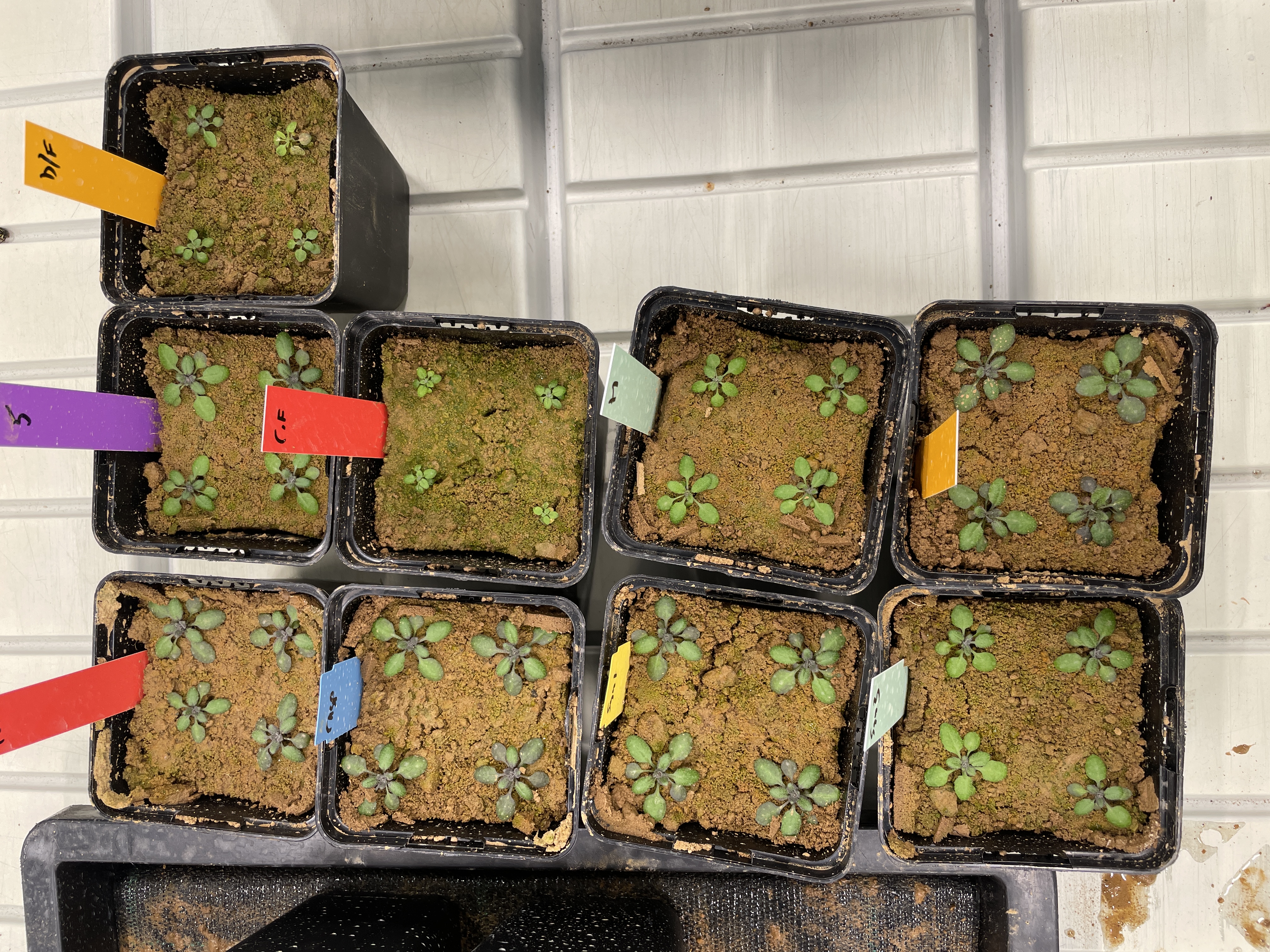

Supplement: Supplementary file 9 — Source data Fig. 5 [file 44318_2024_107_MOESM9_ESM.zip › Figure 5/Figure 5A/Calcinit/IMG_4763.JPG]

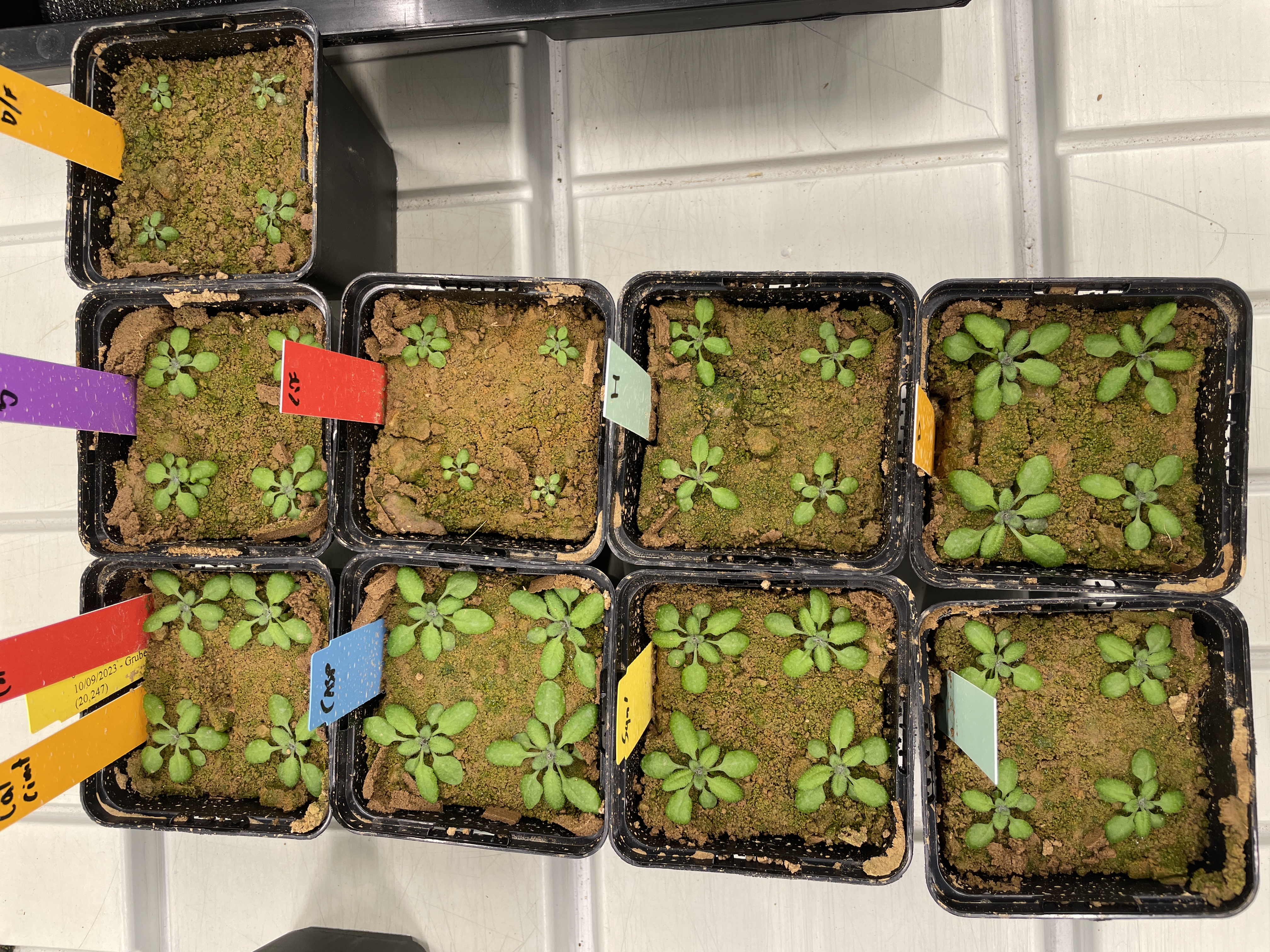

Supplement: Supplementary file 9 — Source data Fig. 5 [file 44318_2024_107_MOESM9_ESM.zip › Figure 5/Figure 5A/Calcinit/IMG_4764.JPG]

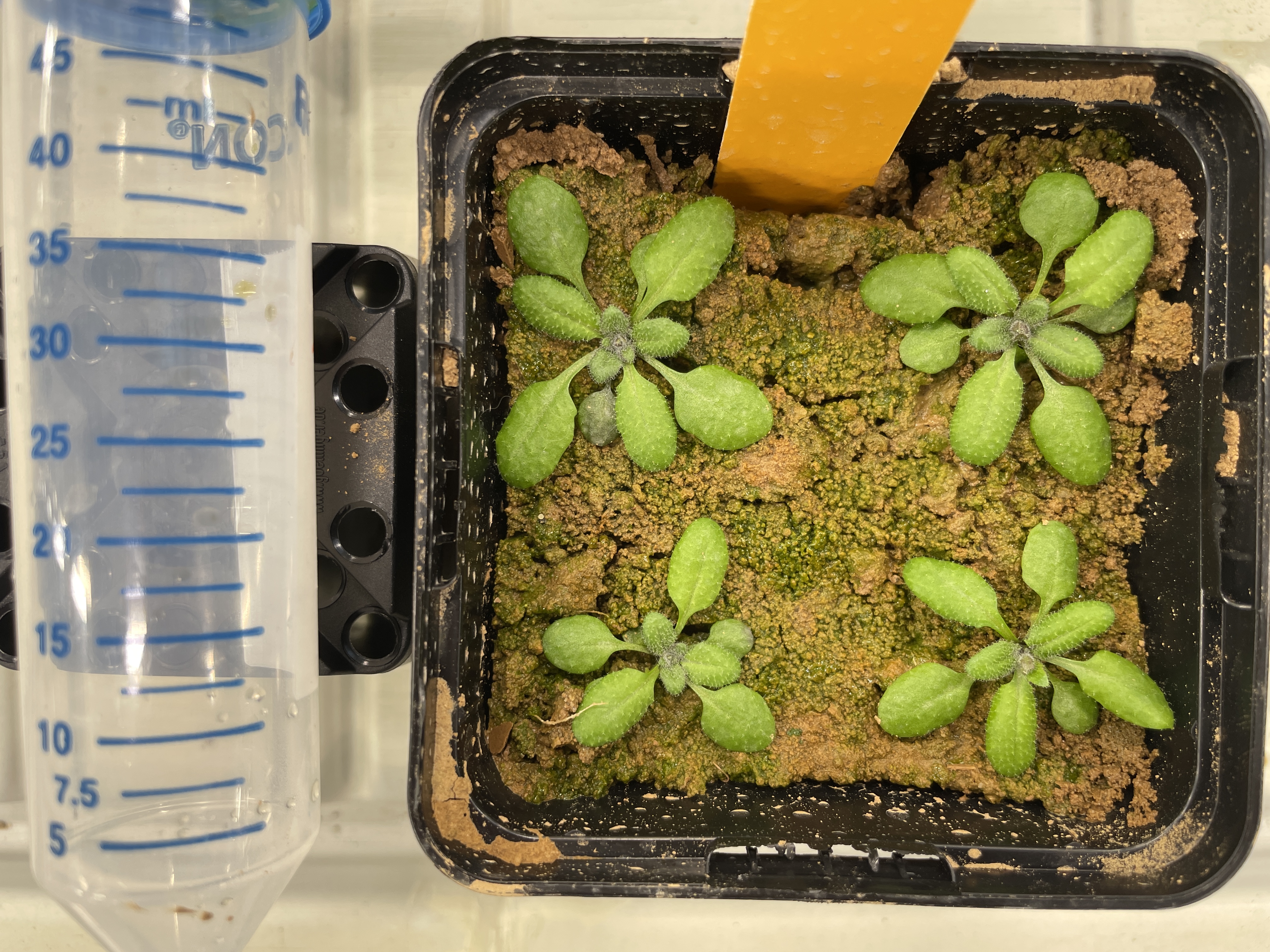

Supplement: Supplementary file 9 — Source data Fig. 5 [file 44318_2024_107_MOESM9_ESM.zip › Figure 5/Figure 5A/Calcinit/rbohd.JPG]

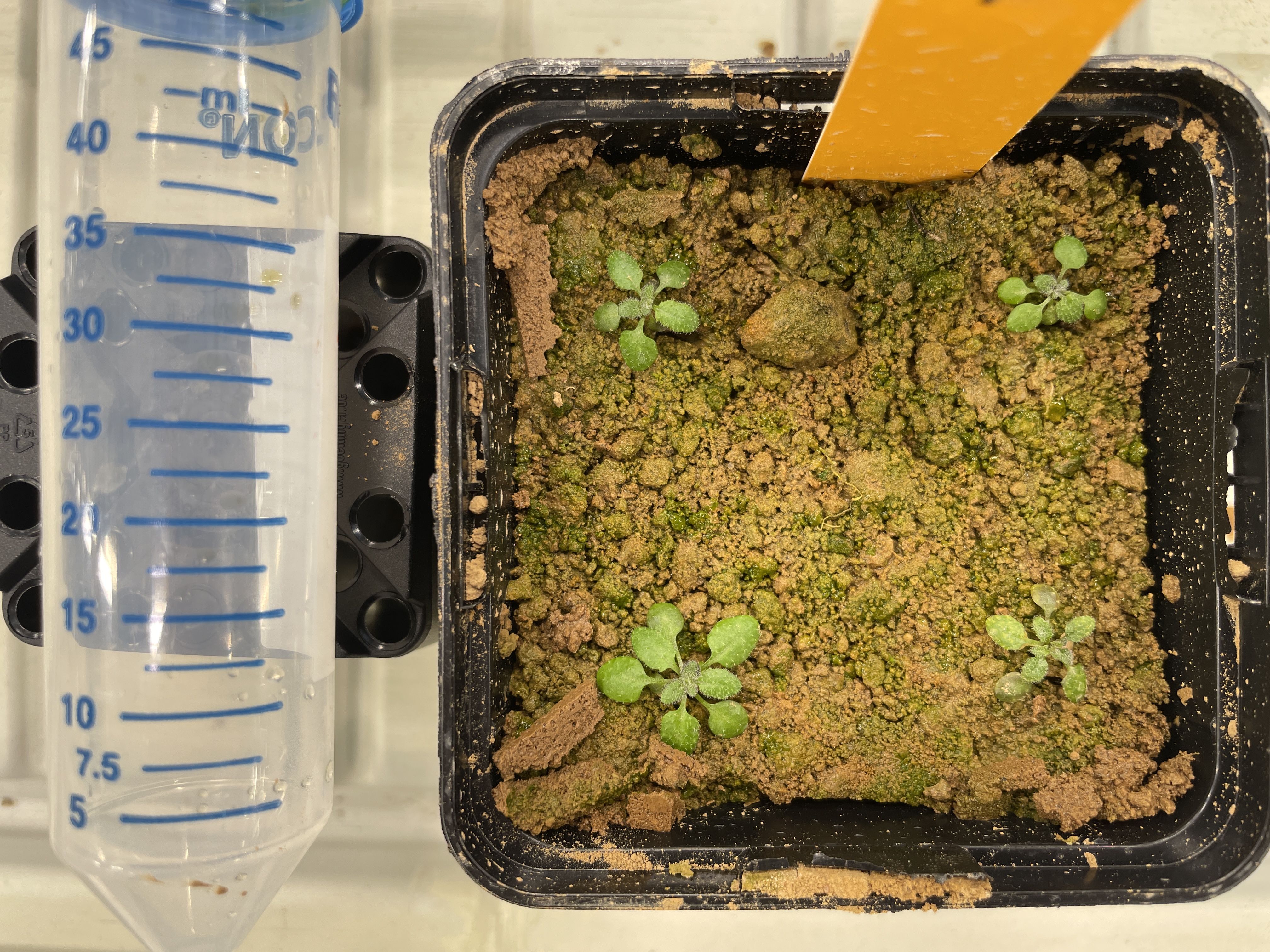

Supplement: Supplementary file 9 — Source data Fig. 5 [file 44318_2024_107_MOESM9_ESM.zip › Figure 5/Figure 5A/Calcinit/rbohdf.JPG]

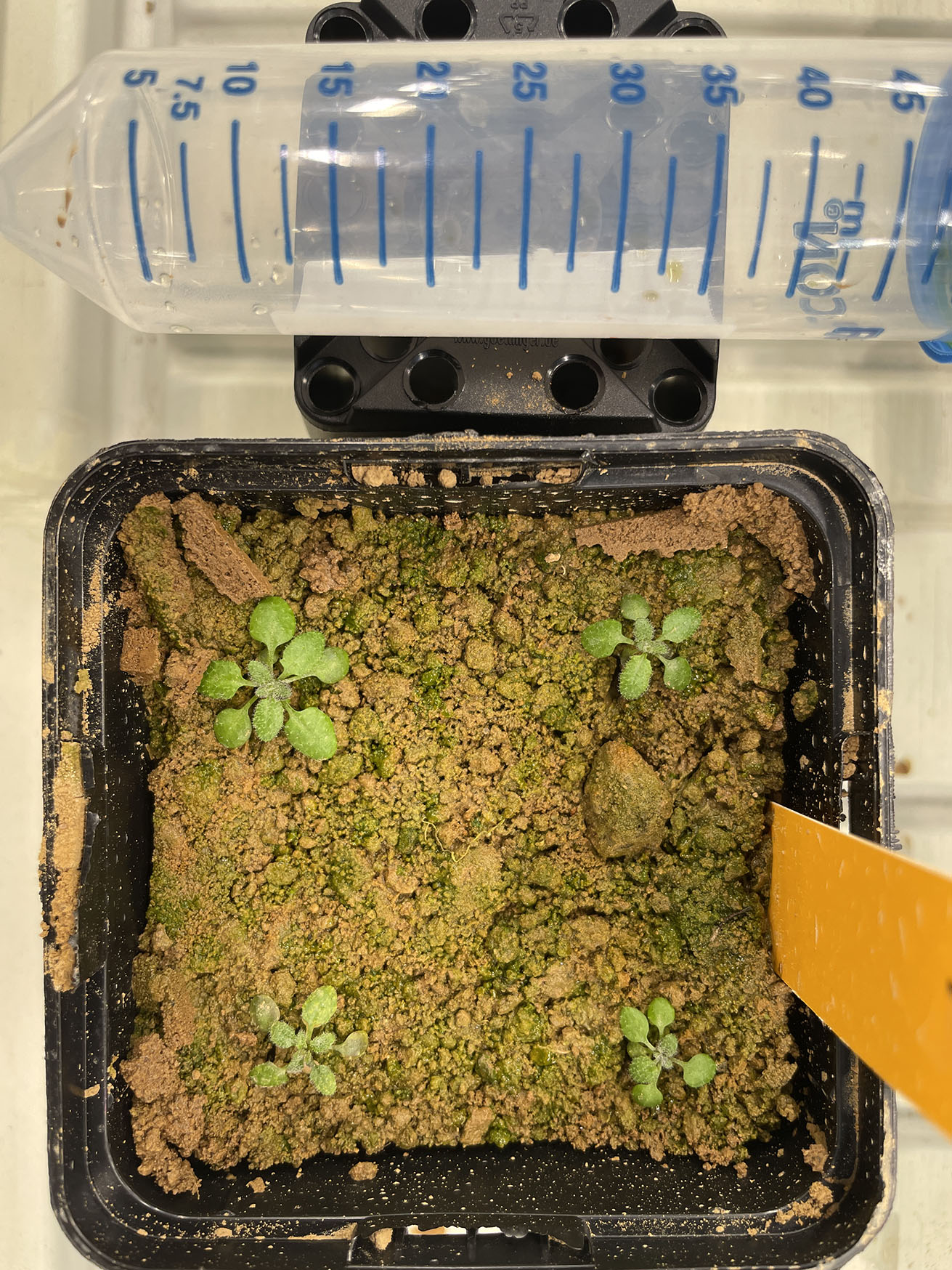

Supplement: Supplementary file 9 — Source data Fig. 5 [file 44318_2024_107_MOESM9_ESM.zip › Figure 5/Figure 5A/Calcinit/rbohdf_small.jpg]

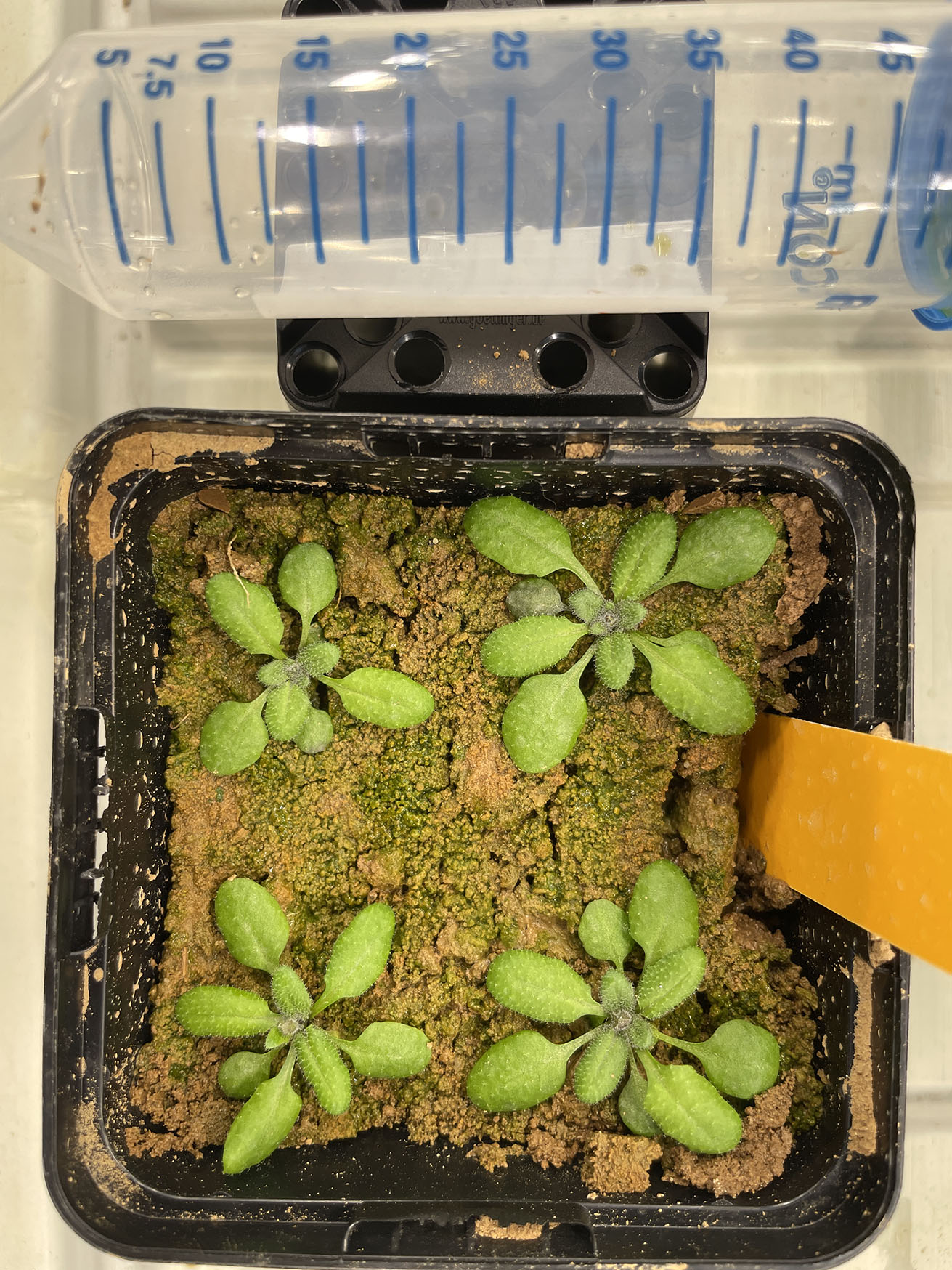

Supplement: Supplementary file 9 — Source data Fig. 5 [file 44318_2024_107_MOESM9_ESM.zip › Figure 5/Figure 5A/Calcinit/rbohd_small.jpg]

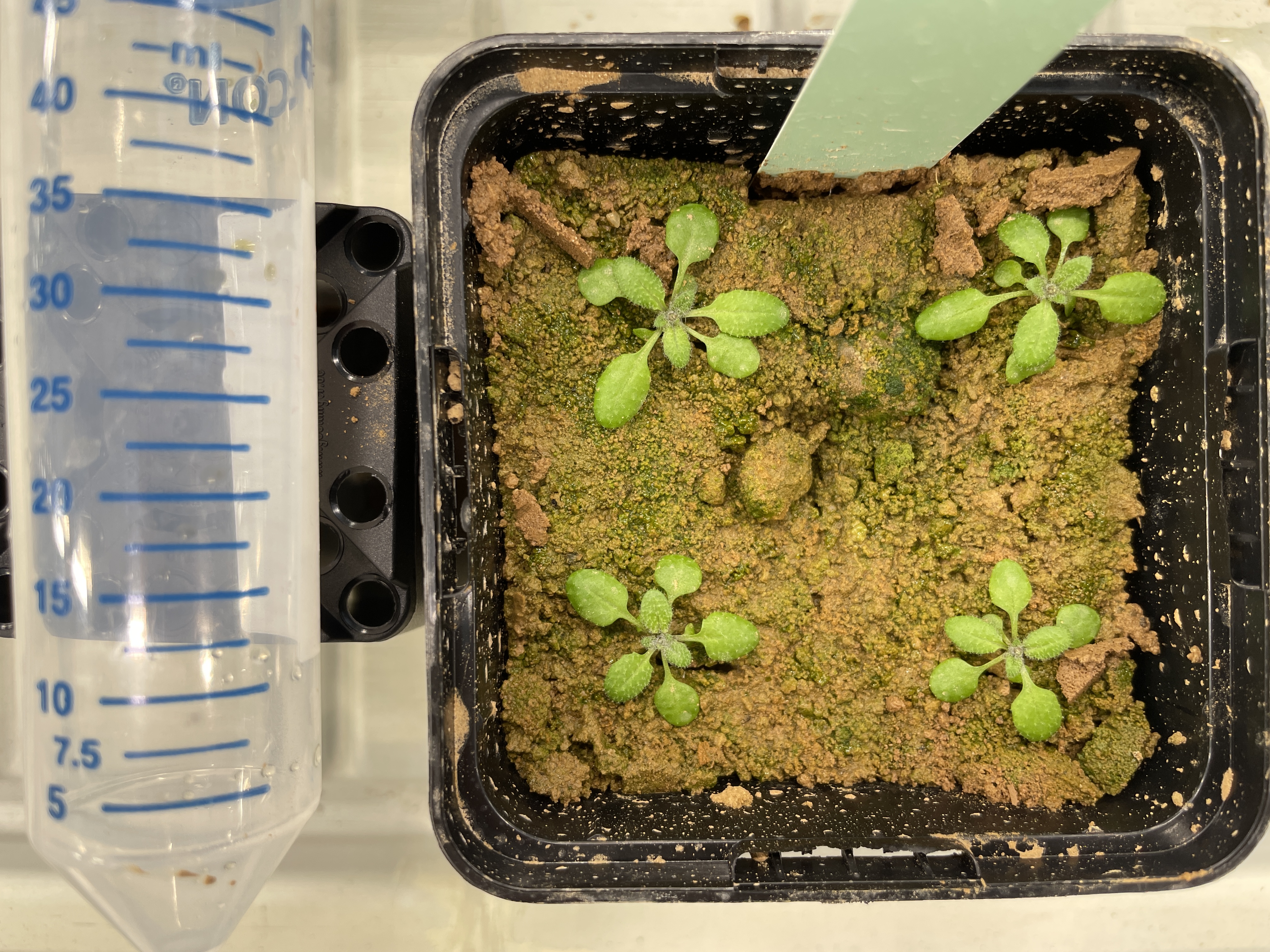

Supplement: Supplementary file 9 — Source data Fig. 5 [file 44318_2024_107_MOESM9_ESM.zip › Figure 5/Figure 5A/Calcinit/rhohF.JPG]

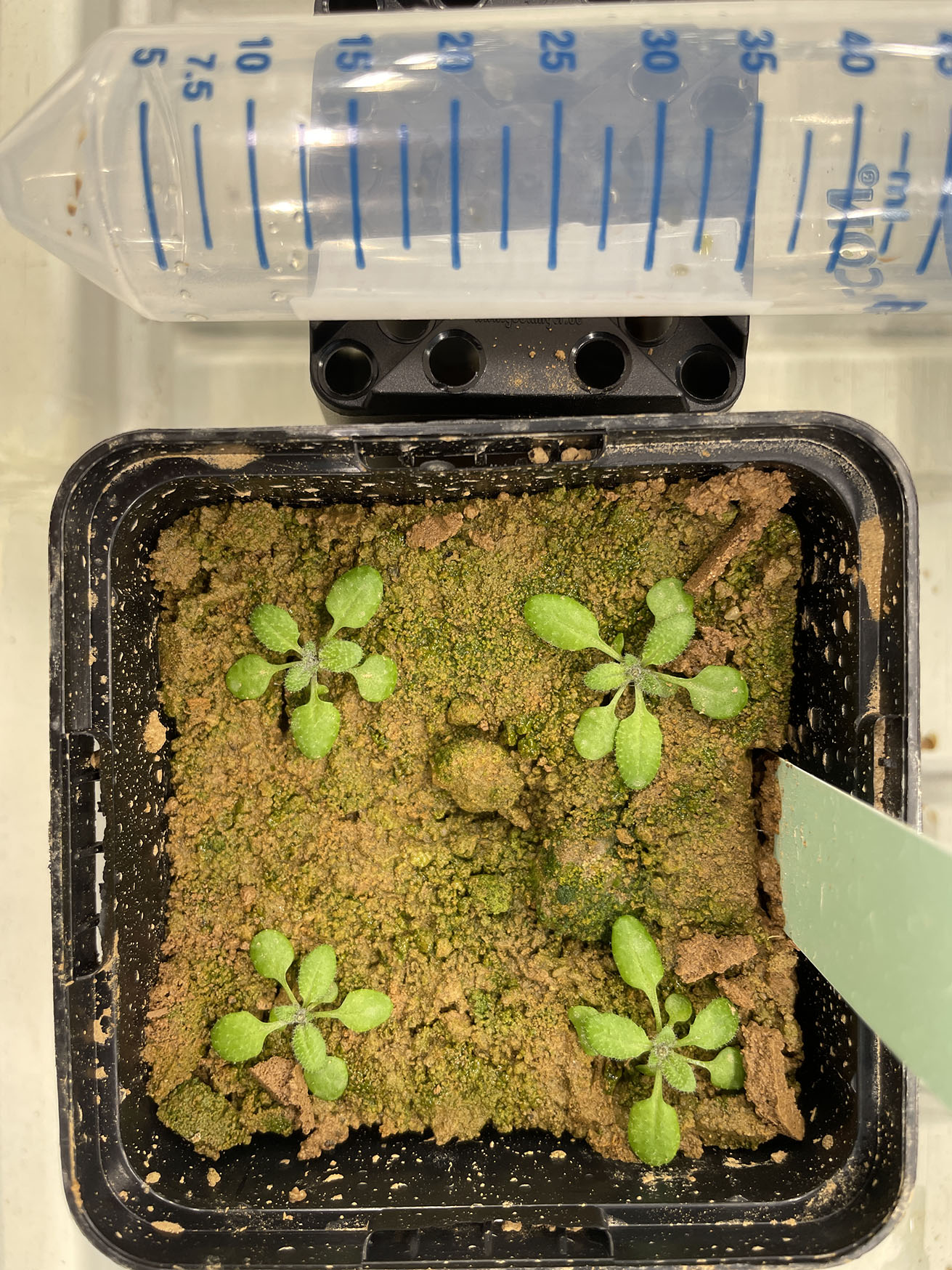

Supplement: Supplementary file 9 — Source data Fig. 5 [file 44318_2024_107_MOESM9_ESM.zip › Figure 5/Figure 5A/Calcinit/rhohF_small.jpg]

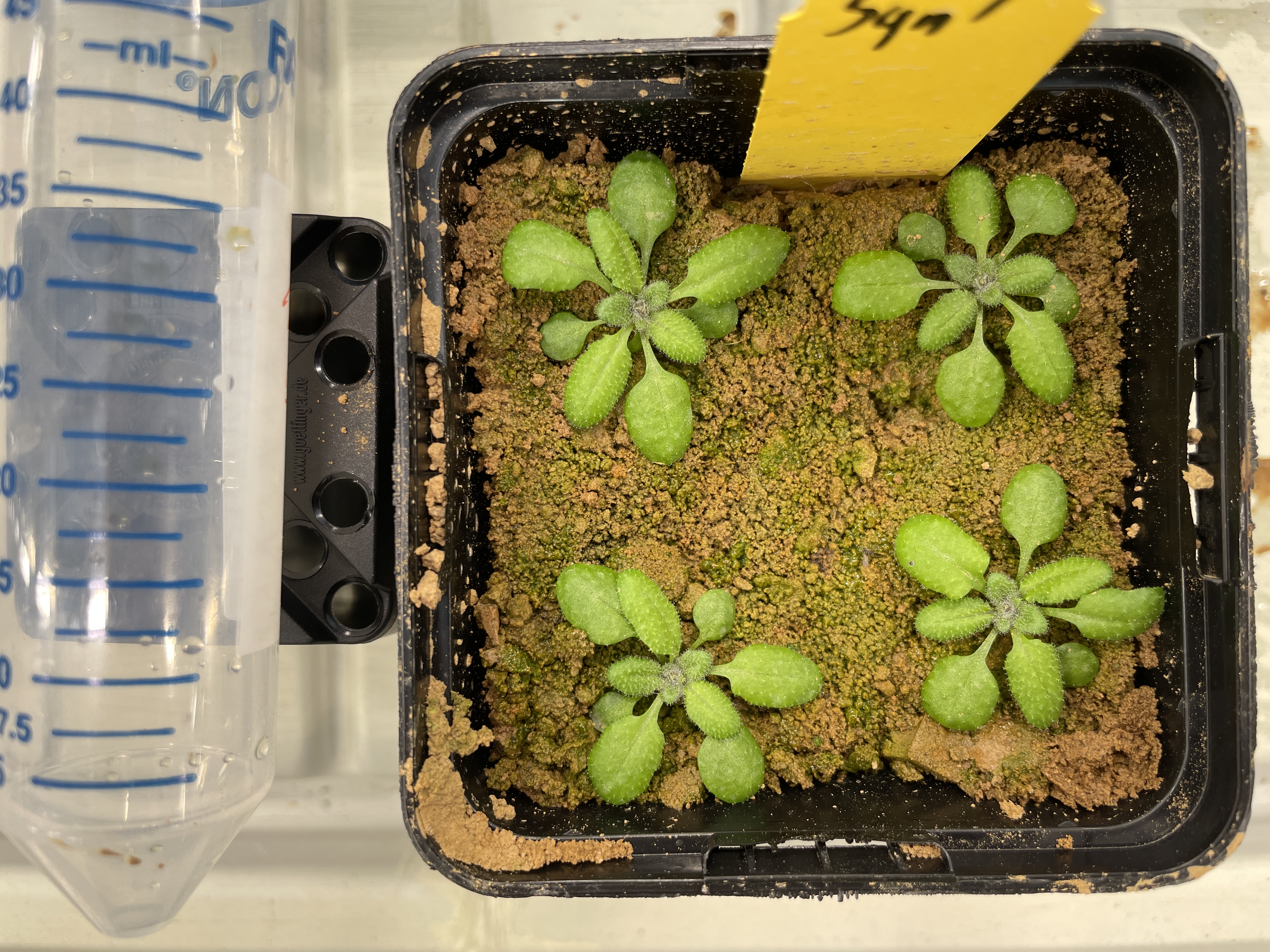

Supplement: Supplementary file 9 — Source data Fig. 5 [file 44318_2024_107_MOESM9_ESM.zip › Figure 5/Figure 5A/Calcinit/SGN1.JPG]

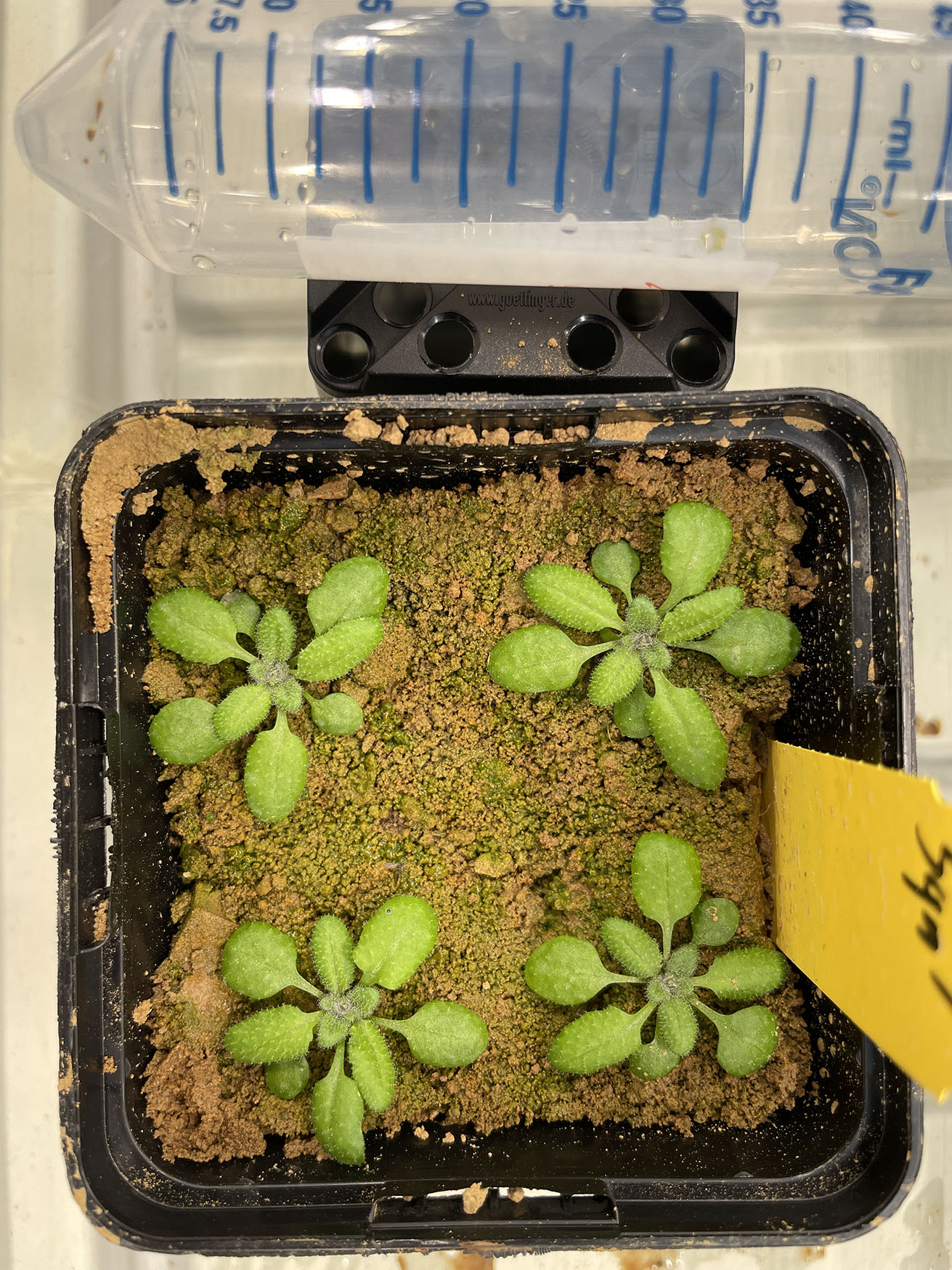

Supplement: Supplementary file 9 — Source data Fig. 5 [file 44318_2024_107_MOESM9_ESM.zip › Figure 5/Figure 5A/Calcinit/SGN1_small.jpg]

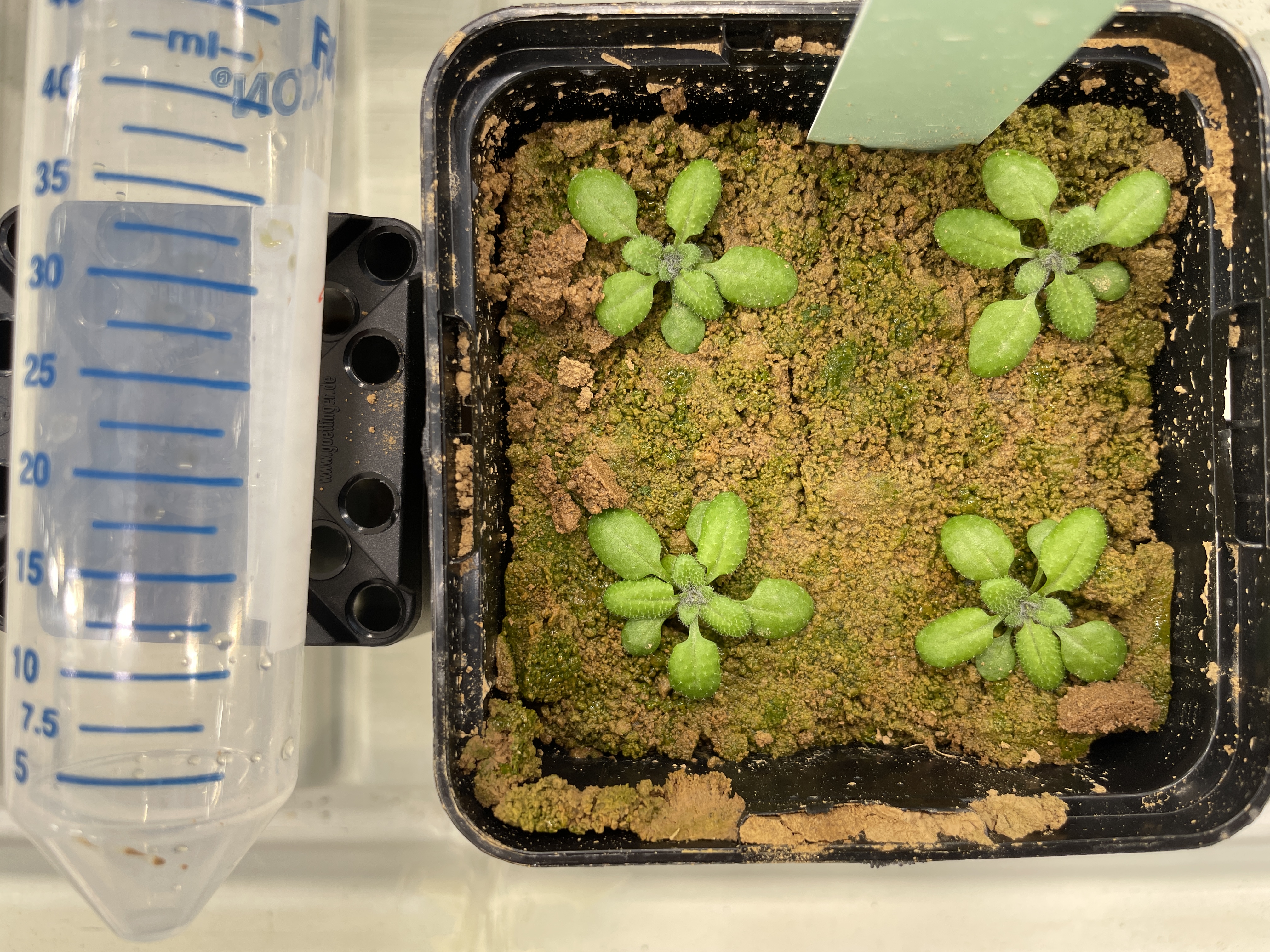

Supplement: Supplementary file 9 — Source data Fig. 5 [file 44318_2024_107_MOESM9_ESM.zip › Figure 5/Figure 5A/Calcinit/SGN3.JPG]

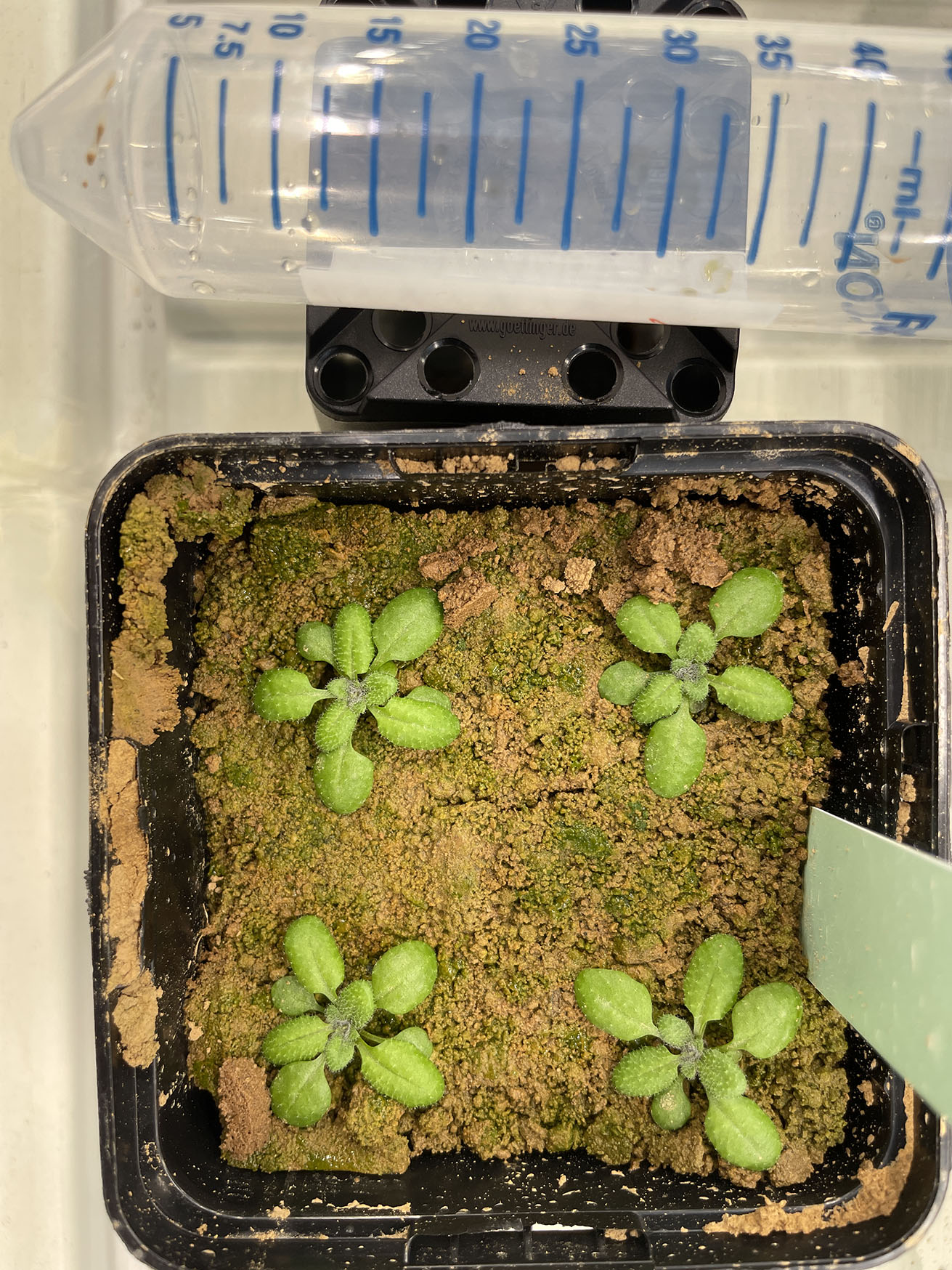

Supplement: Supplementary file 9 — Source data Fig. 5 [file 44318_2024_107_MOESM9_ESM.zip › Figure 5/Figure 5A/Calcinit/SGN3_small.jpg]

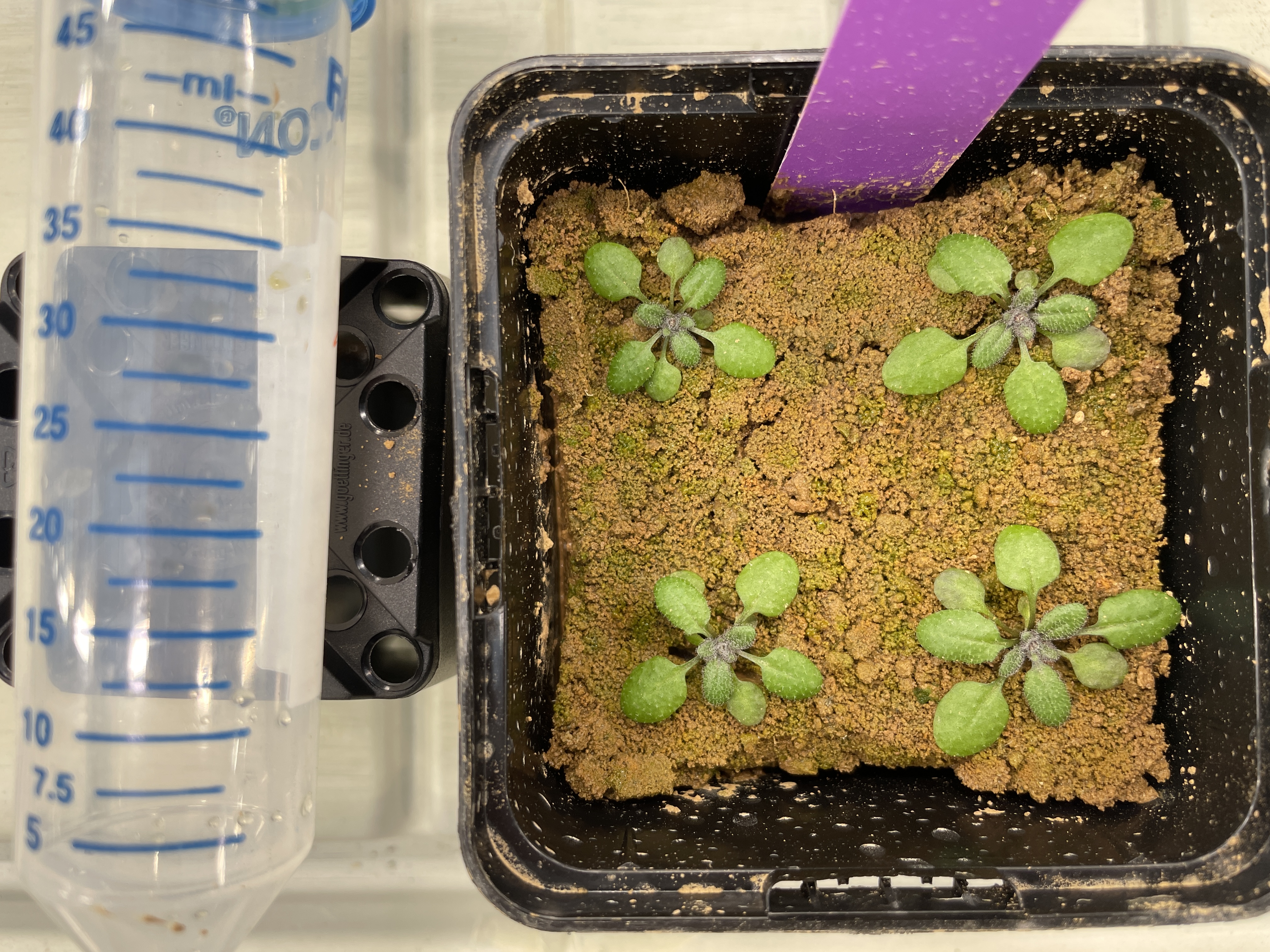

Supplement: Supplementary file 9 — Source data Fig. 5 [file 44318_2024_107_MOESM9_ESM.zip › Figure 5/Figure 5A/H2O/BL5.JPG]

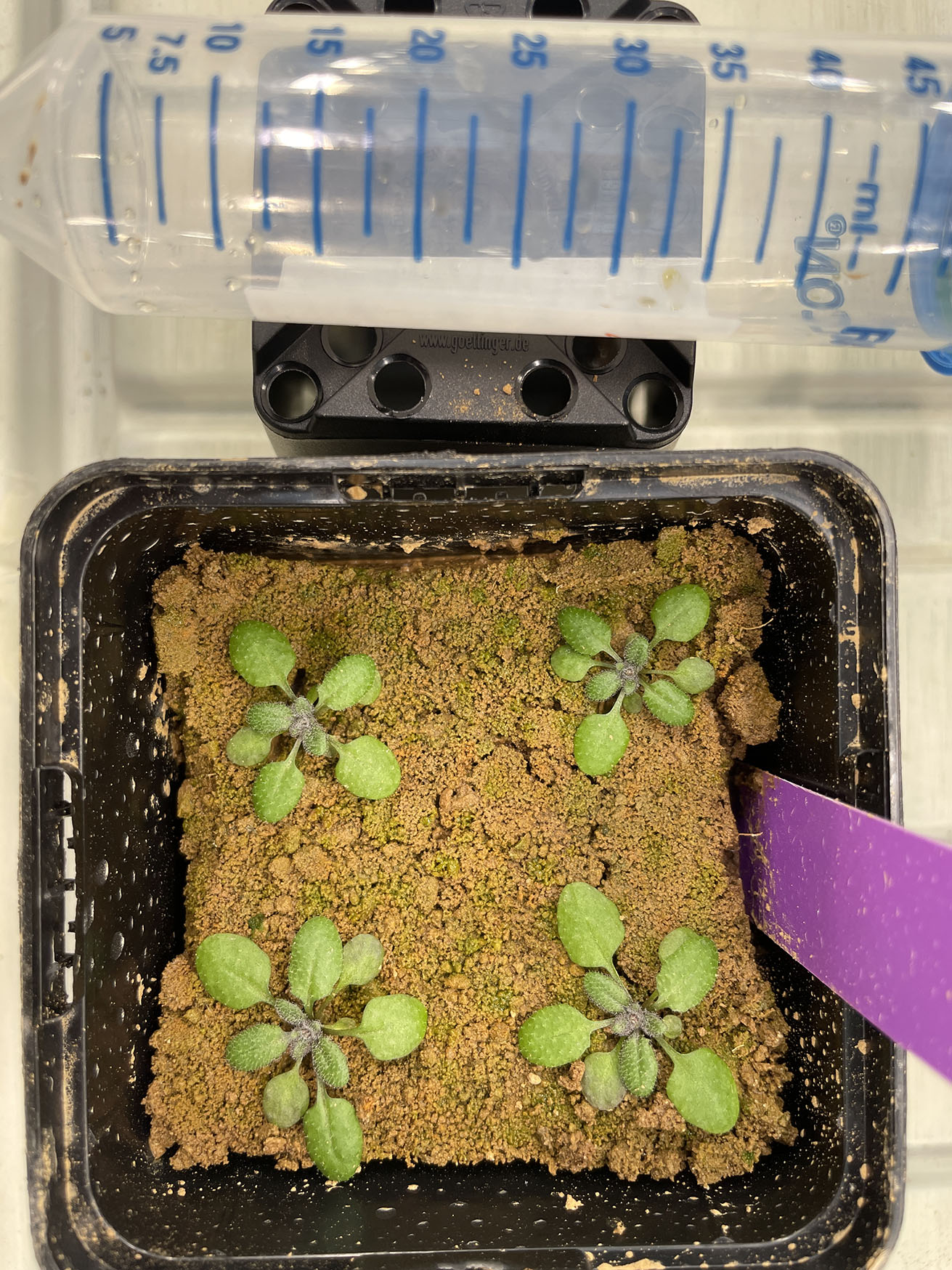

Supplement: Supplementary file 9 — Source data Fig. 5 [file 44318_2024_107_MOESM9_ESM.zip › Figure 5/Figure 5A/H2O/BL5_small.jpg]

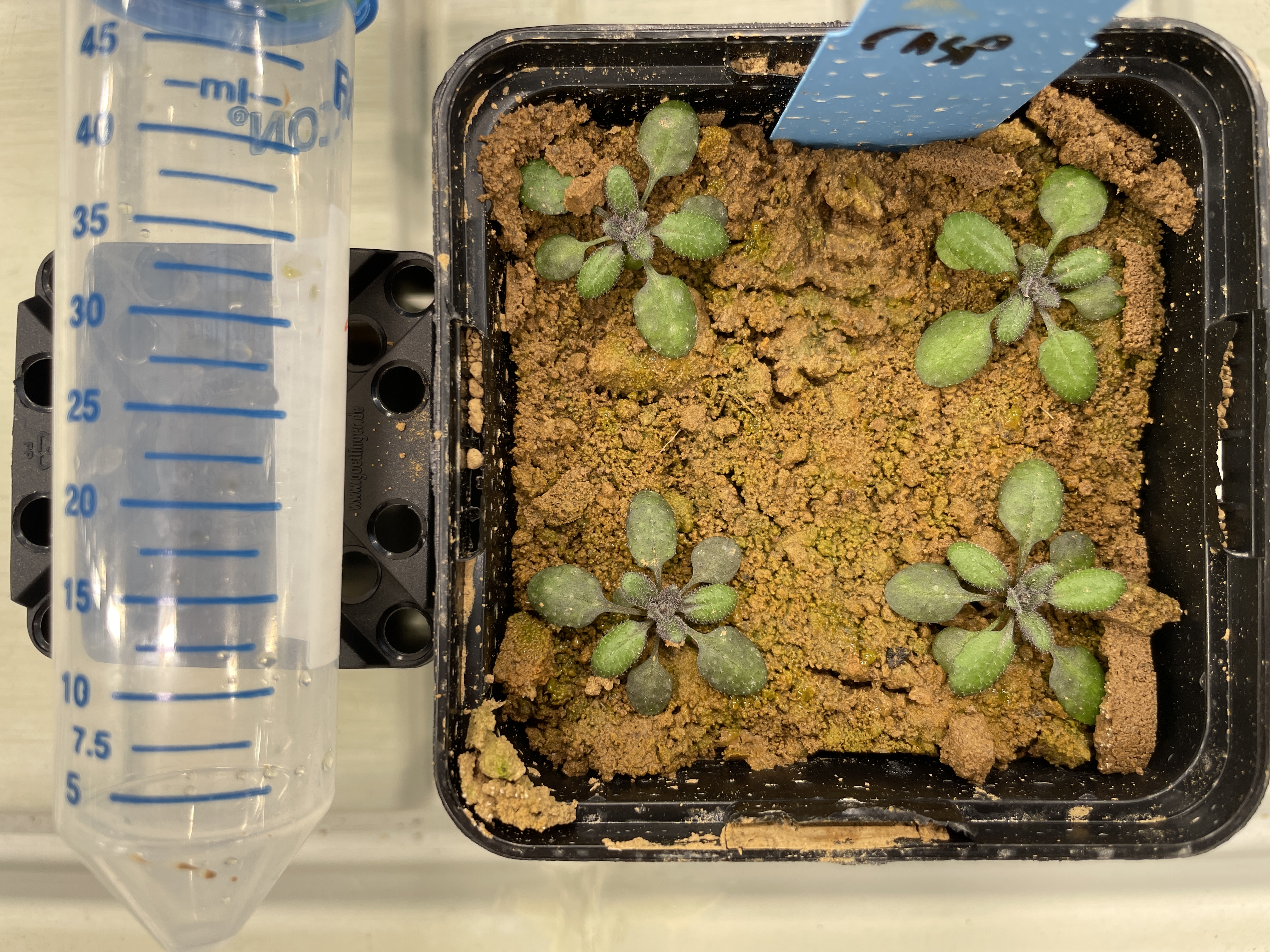

Supplement: Supplementary file 9 — Source data Fig. 5 [file 44318_2024_107_MOESM9_ESM.zip › Figure 5/Figure 5A/H2O/CASP.JPG]

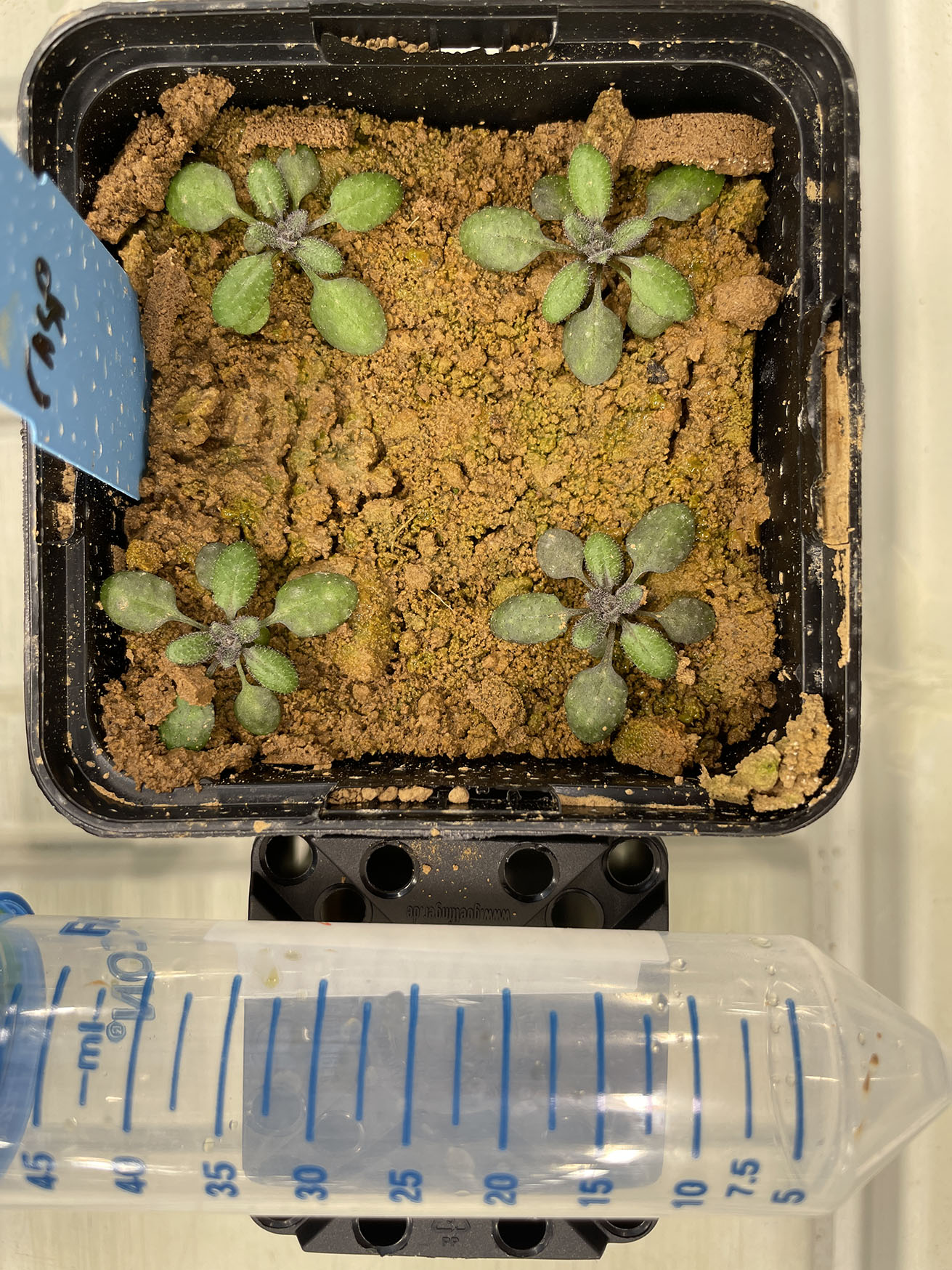

Supplement: Supplementary file 9 — Source data Fig. 5 [file 44318_2024_107_MOESM9_ESM.zip › Figure 5/Figure 5A/H2O/CASP_small.jpg]

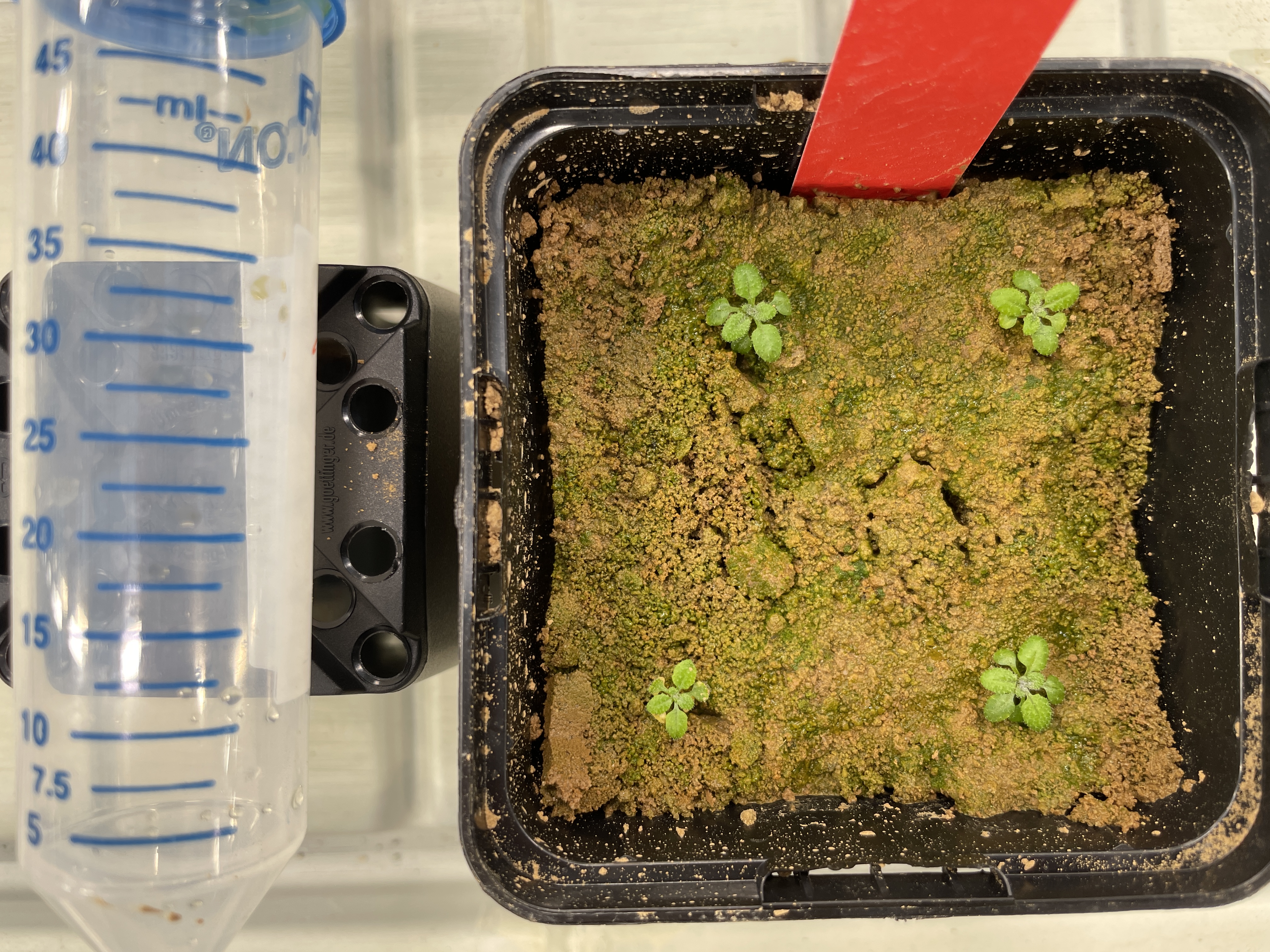

Supplement: Supplementary file 9 — Source data Fig. 5 [file 44318_2024_107_MOESM9_ESM.zip › Figure 5/Figure 5A/H2O/CIF.JPG]

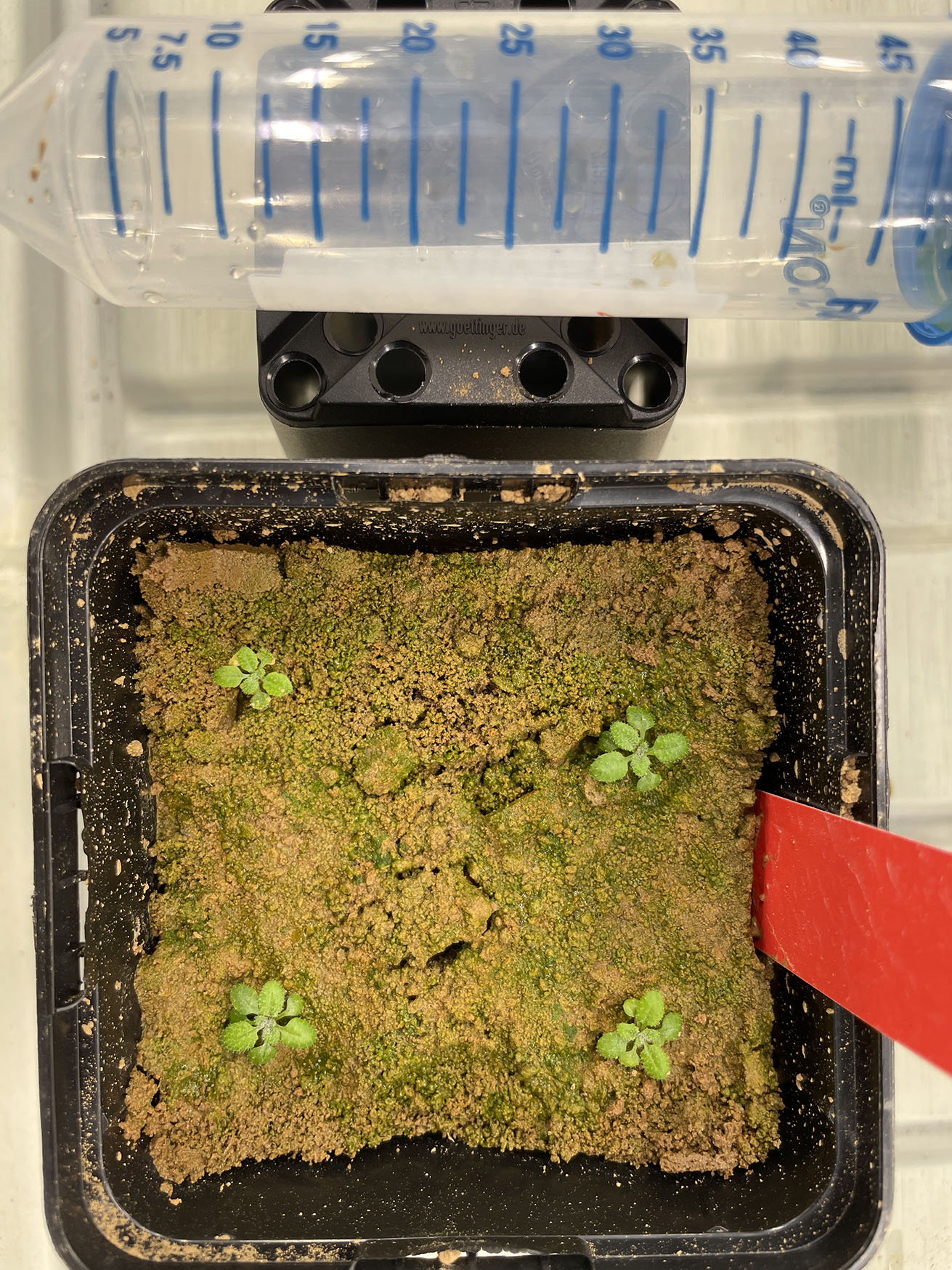

Supplement: Supplementary file 9 — Source data Fig. 5 [file 44318_2024_107_MOESM9_ESM.zip › Figure 5/Figure 5A/H2O/CIF_small.jpg]

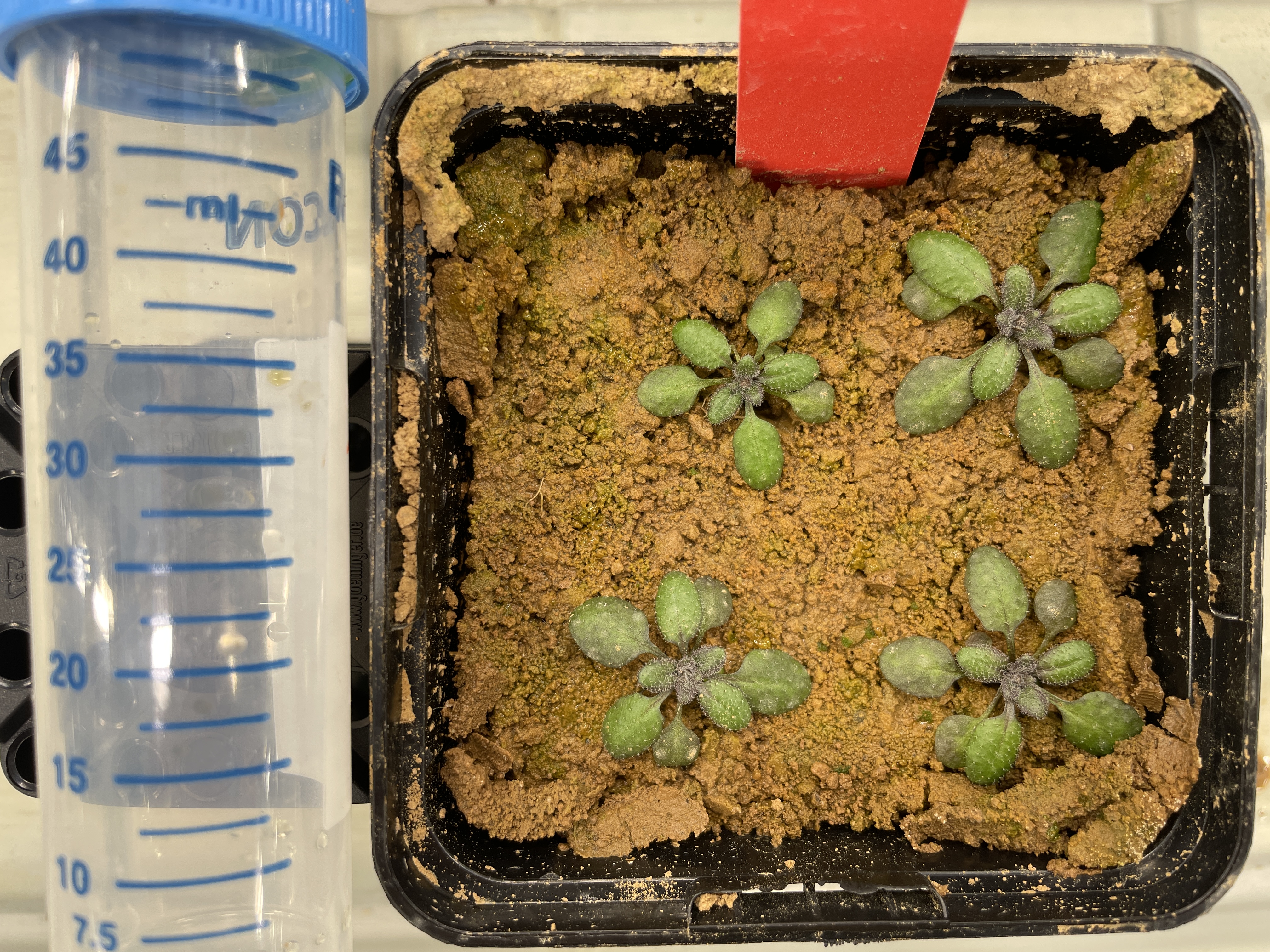

Supplement: Supplementary file 9 — Source data Fig. 5 [file 44318_2024_107_MOESM9_ESM.zip › Figure 5/Figure 5A/H2O/COL.JPG]

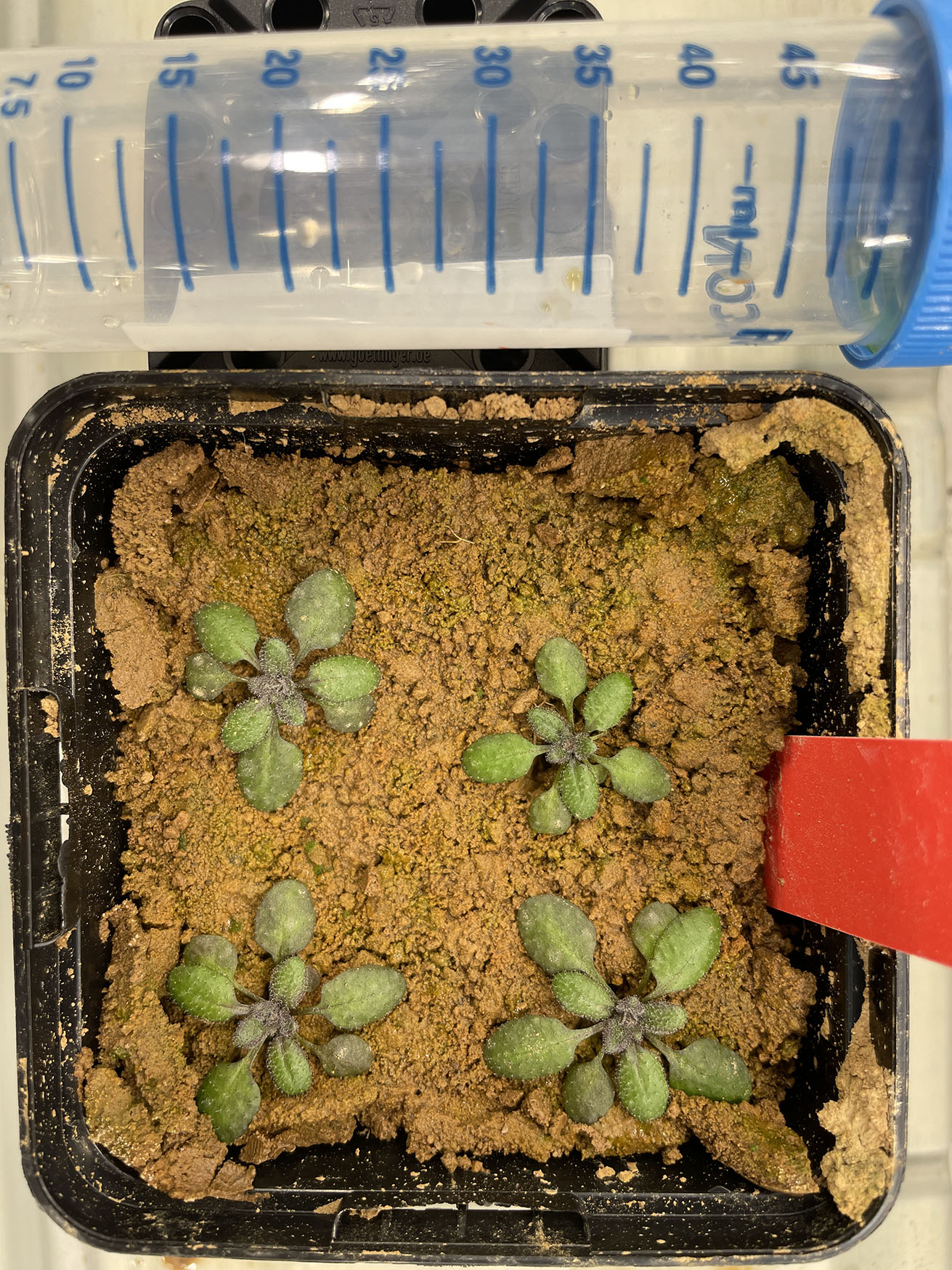

Supplement: Supplementary file 9 — Source data Fig. 5 [file 44318_2024_107_MOESM9_ESM.zip › Figure 5/Figure 5A/H2O/COL_small.jpg]

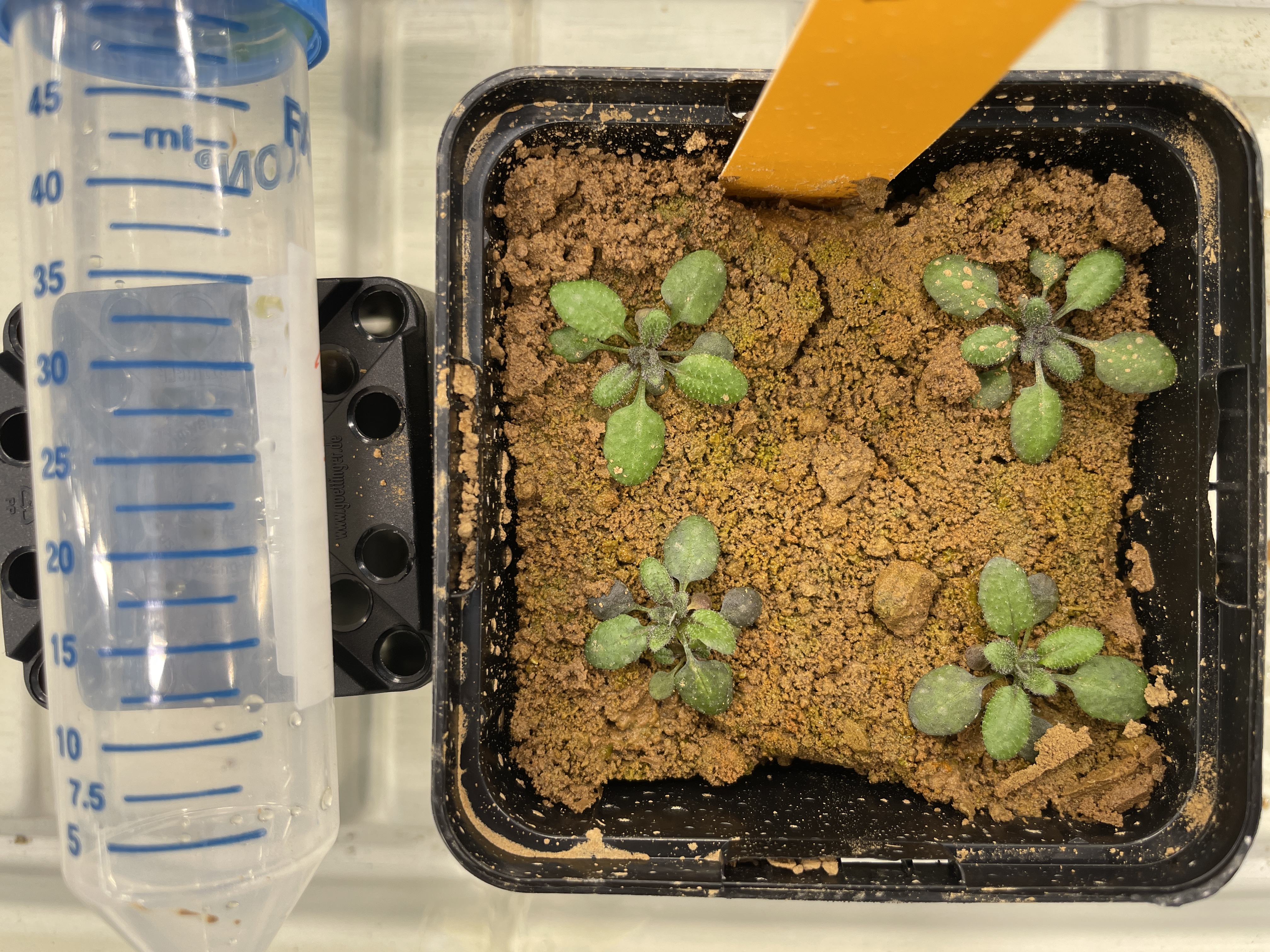

Supplement: Supplementary file 9 — Source data Fig. 5 [file 44318_2024_107_MOESM9_ESM.zip › Figure 5/Figure 5A/H2O/RBOHD.JPG]

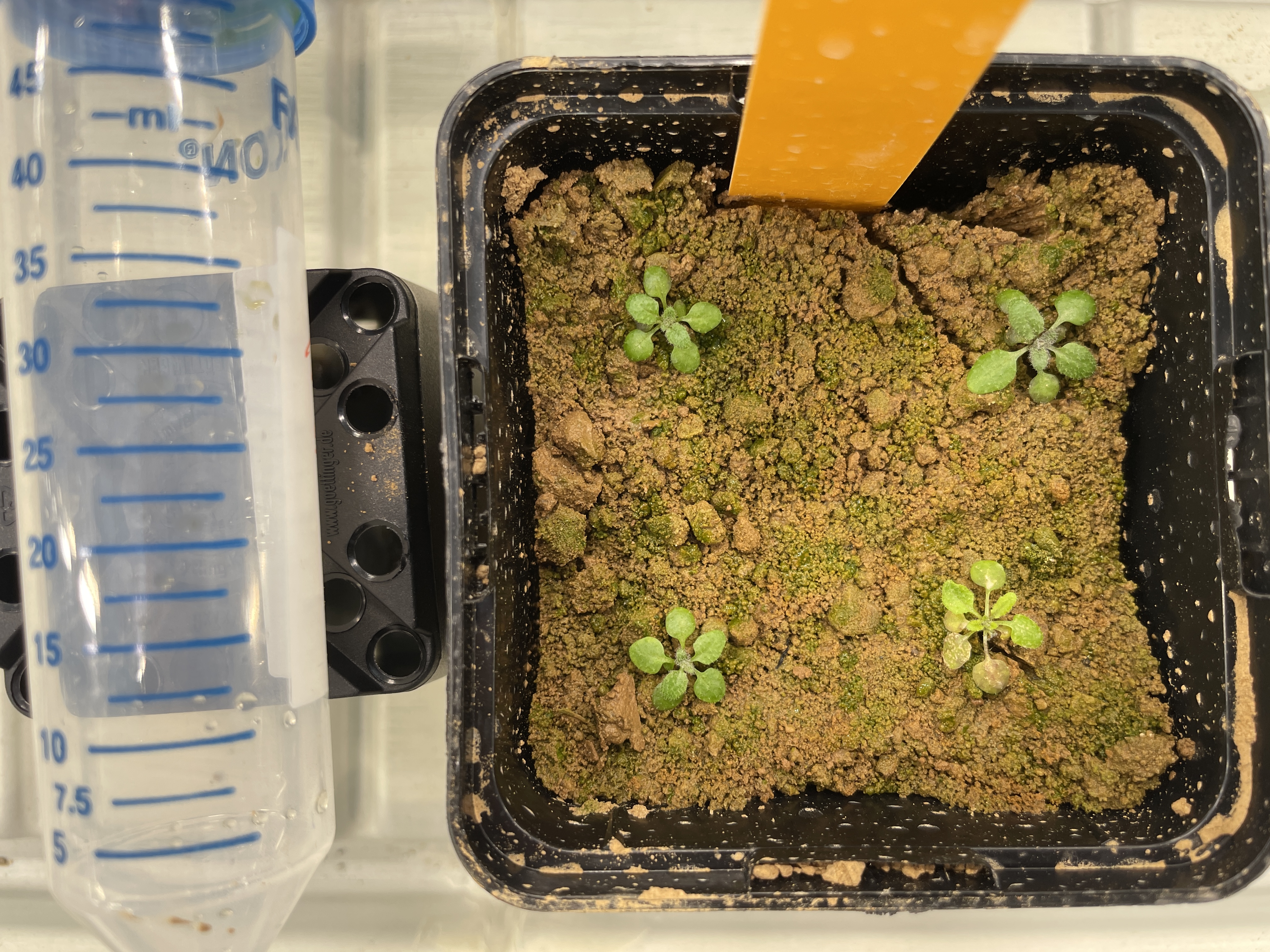

Supplement: Supplementary file 9 — Source data Fig. 5 [file 44318_2024_107_MOESM9_ESM.zip › Figure 5/Figure 5A/H2O/RBOHDF.JPG]

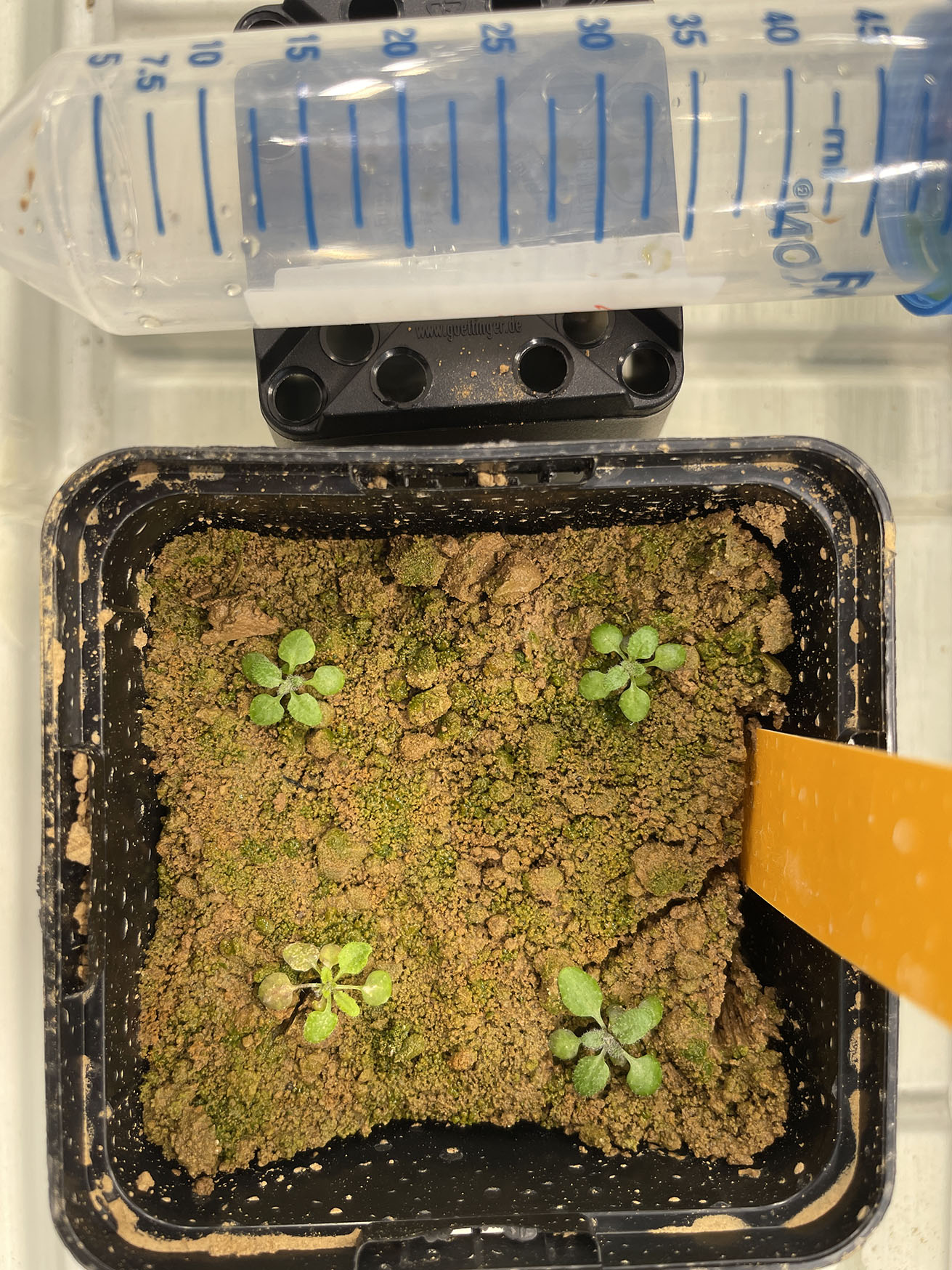

Supplement: Supplementary file 9 — Source data Fig. 5 [file 44318_2024_107_MOESM9_ESM.zip › Figure 5/Figure 5A/H2O/RBOHDF_small.jpg]

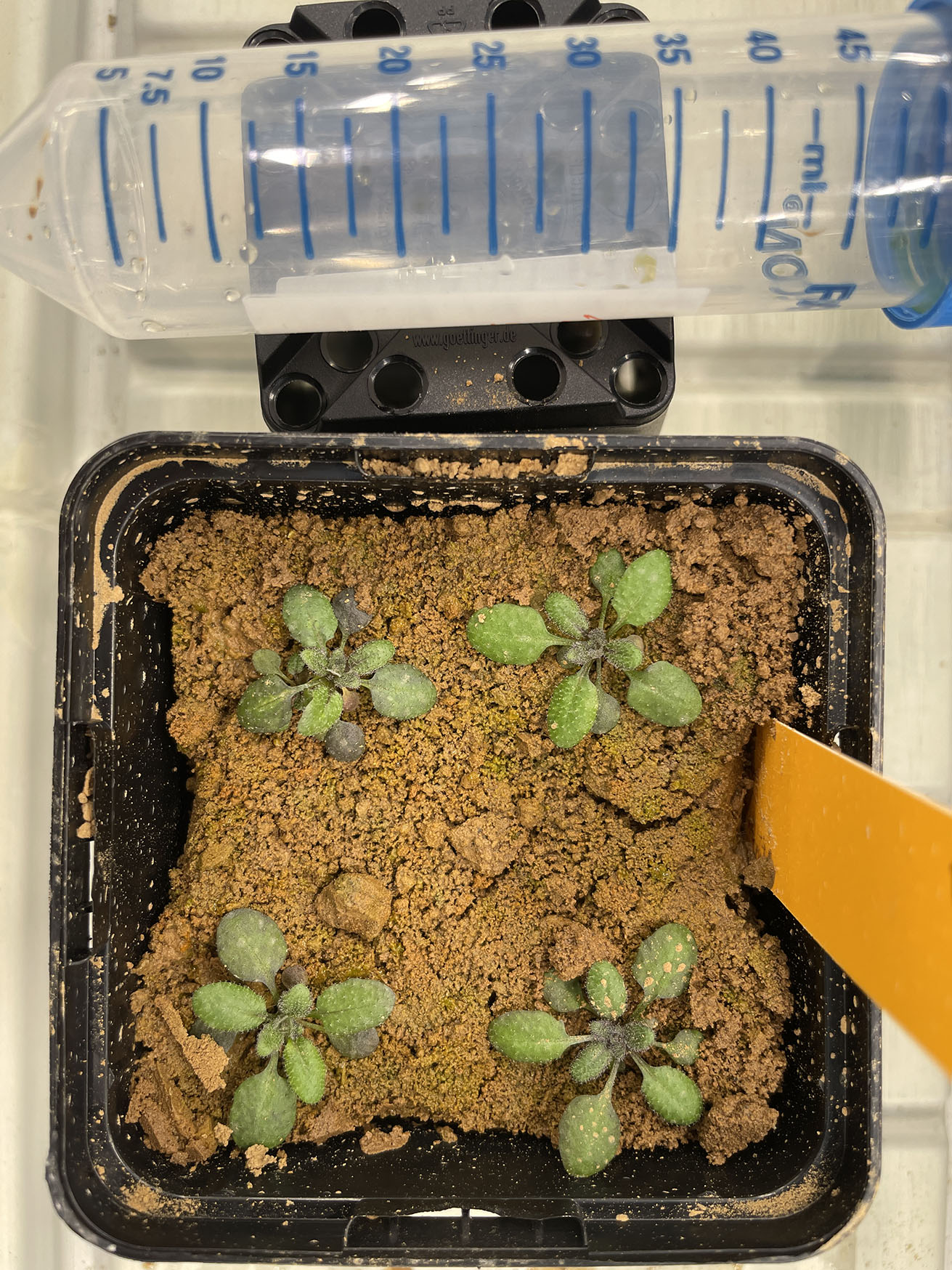

Supplement: Supplementary file 9 — Source data Fig. 5 [file 44318_2024_107_MOESM9_ESM.zip › Figure 5/Figure 5A/H2O/RBOHD_small.jpg]

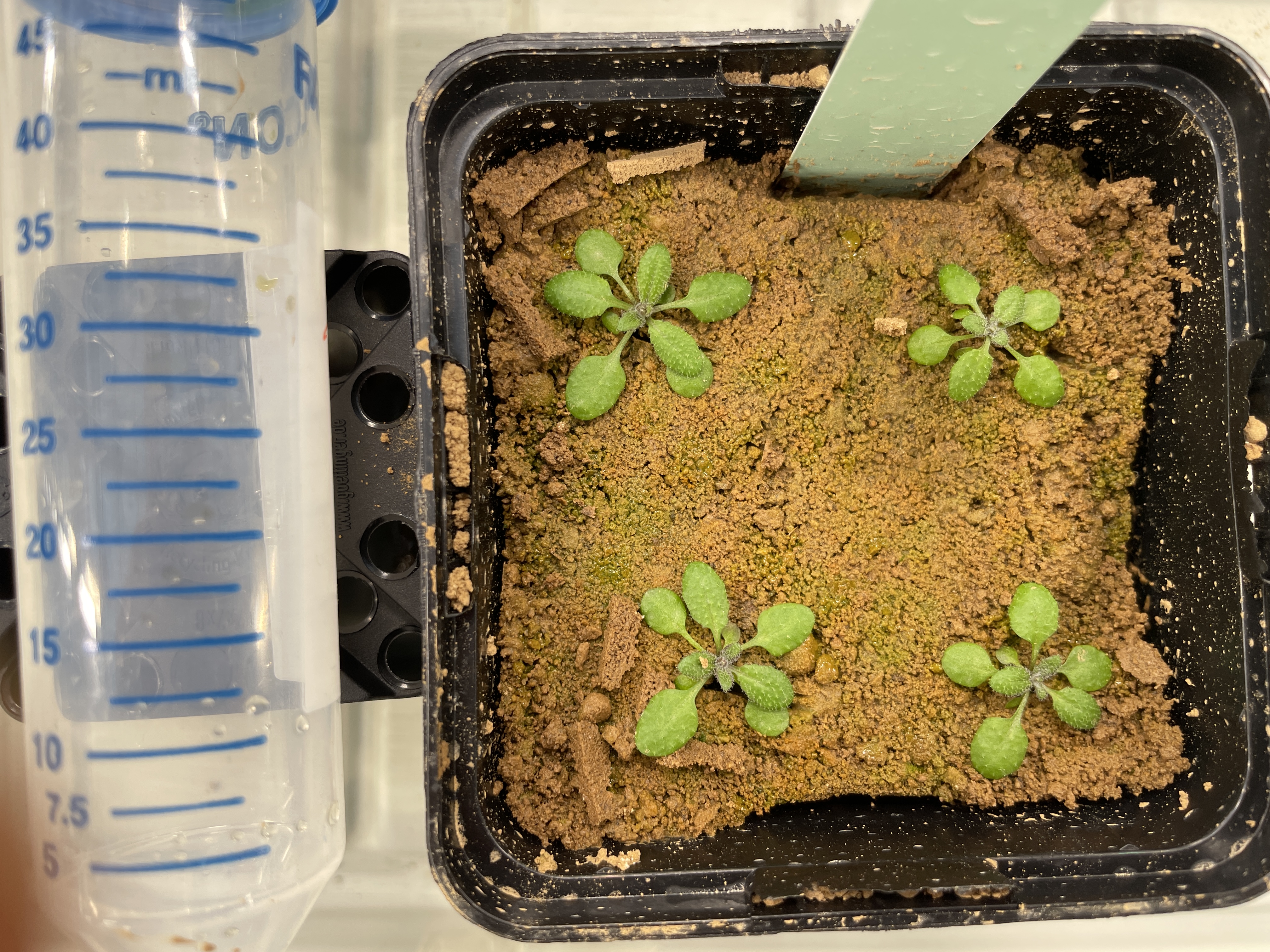

Supplement: Supplementary file 9 — Source data Fig. 5 [file 44318_2024_107_MOESM9_ESM.zip › Figure 5/Figure 5A/H2O/RBOHF.JPG]

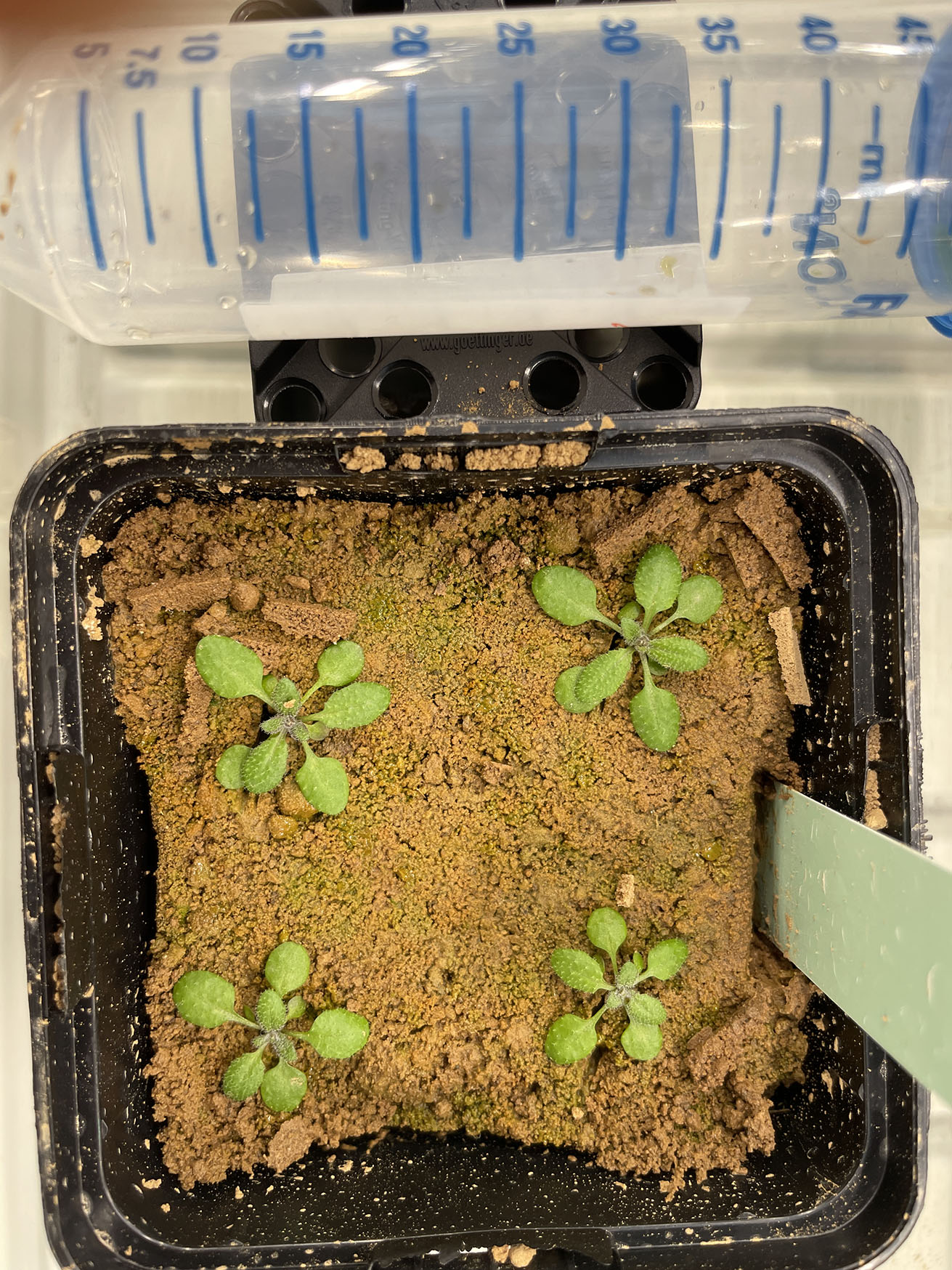

Supplement: Supplementary file 9 — Source data Fig. 5 [file 44318_2024_107_MOESM9_ESM.zip › Figure 5/Figure 5A/H2O/RBOHF_small.jpg]

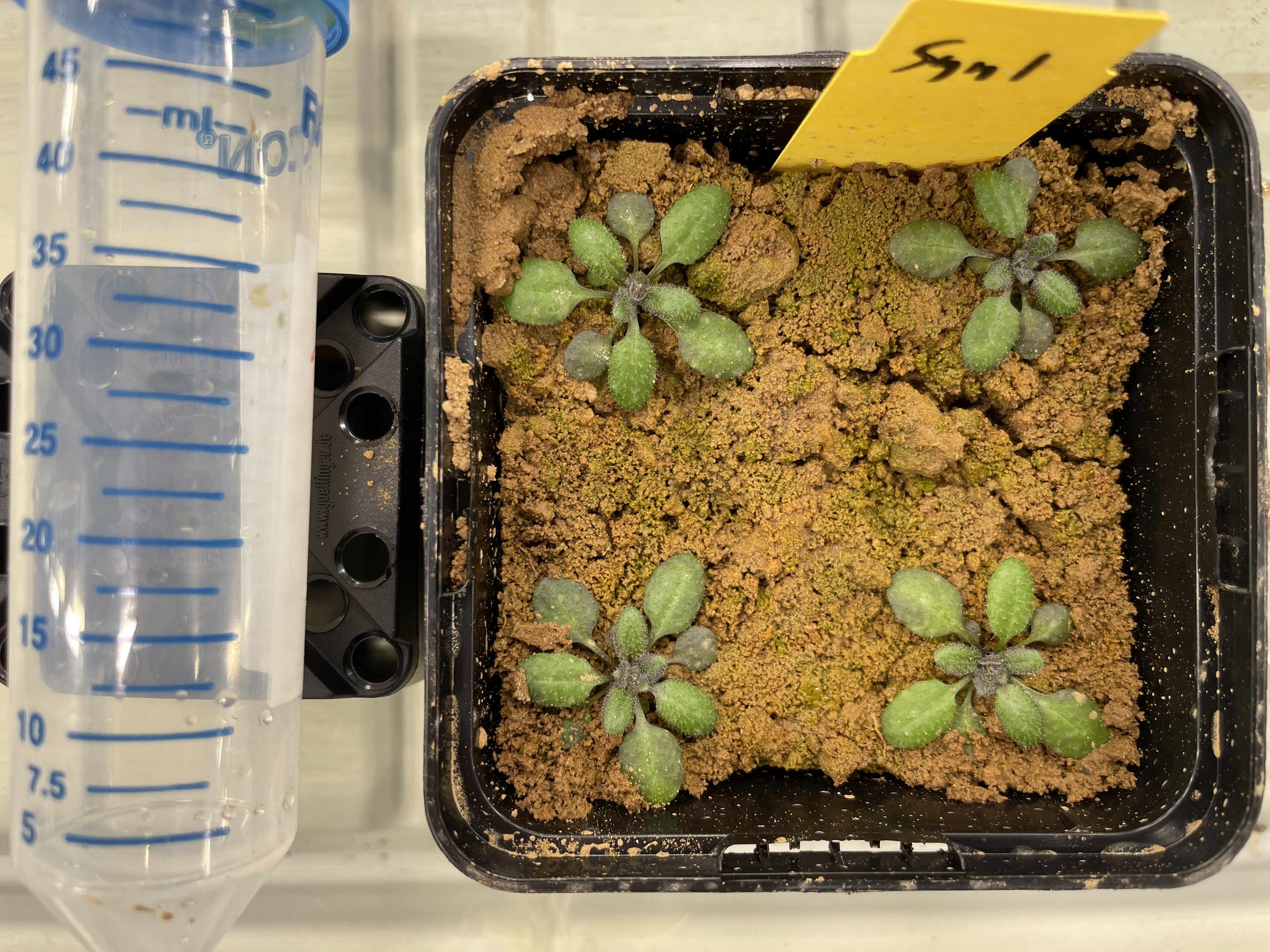

Supplement: Supplementary file 9 — Source data Fig. 5 [file 44318_2024_107_MOESM9_ESM.zip › Figure 5/Figure 5A/H2O/SGN1.JPG]

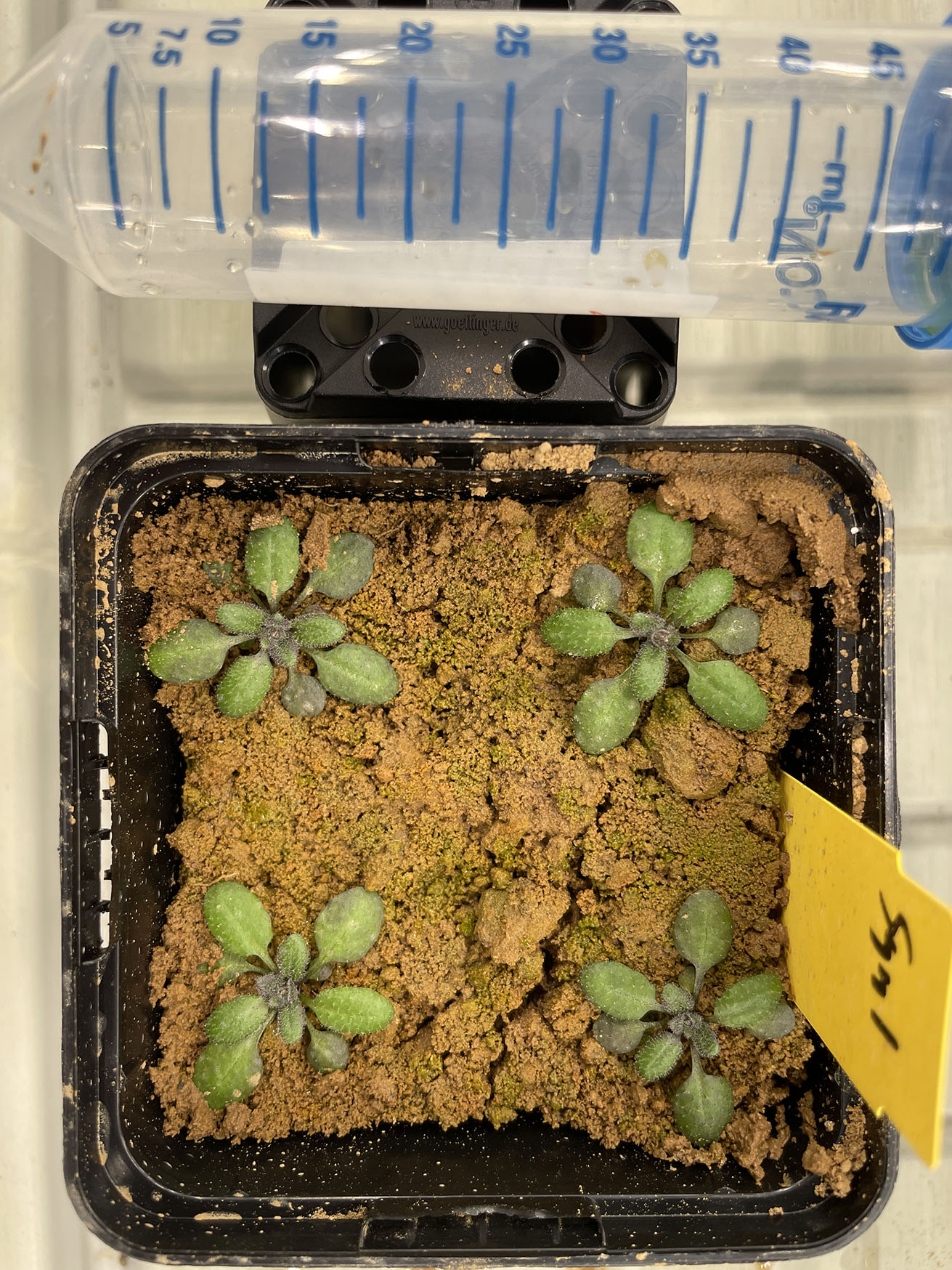

Supplement: Supplementary file 9 — Source data Fig. 5 [file 44318_2024_107_MOESM9_ESM.zip › Figure 5/Figure 5A/H2O/SGN1_small.jpg]

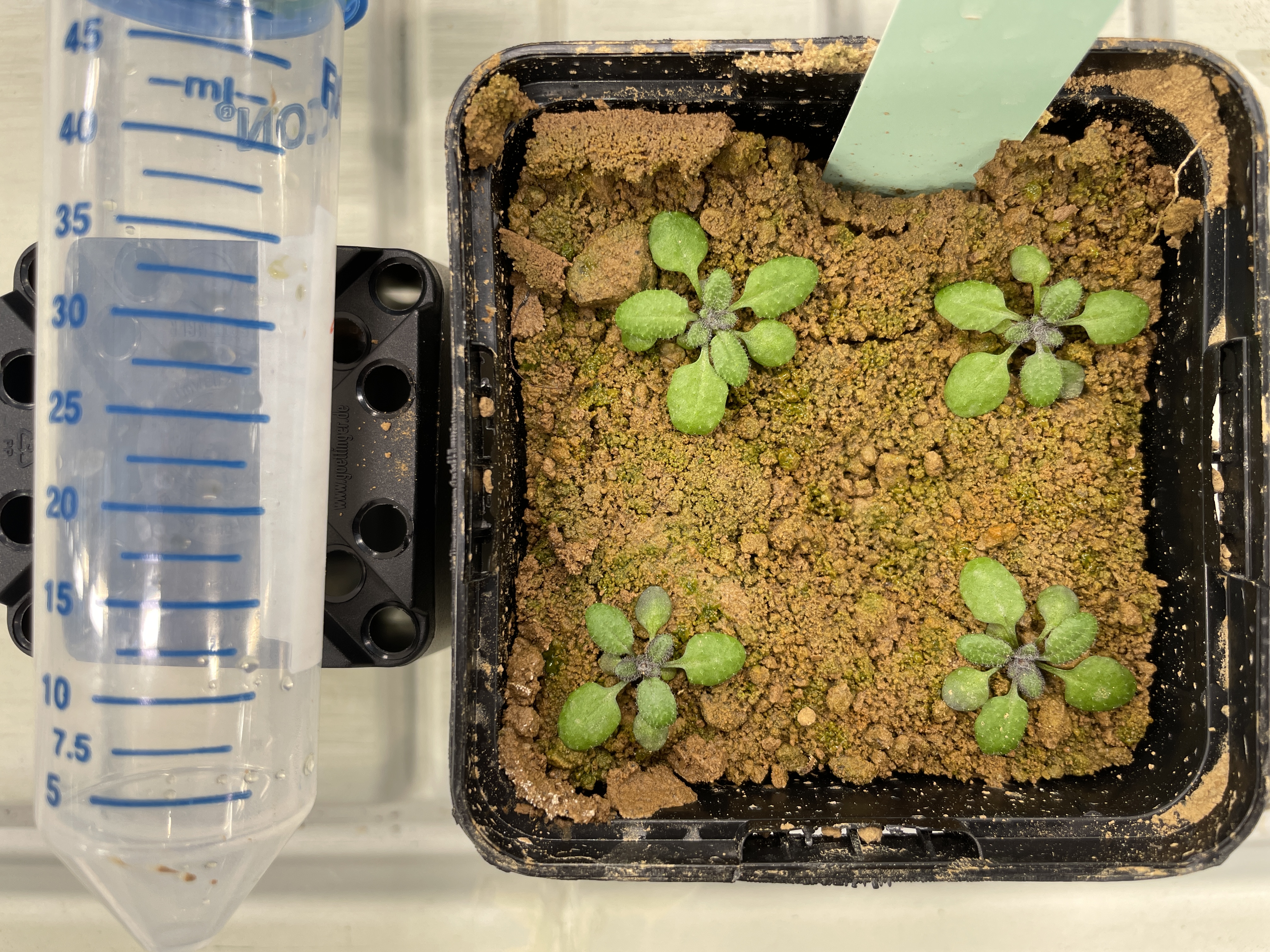

Supplement: Supplementary file 9 — Source data Fig. 5 [file 44318_2024_107_MOESM9_ESM.zip › Figure 5/Figure 5A/H2O/SGN3.JPG]

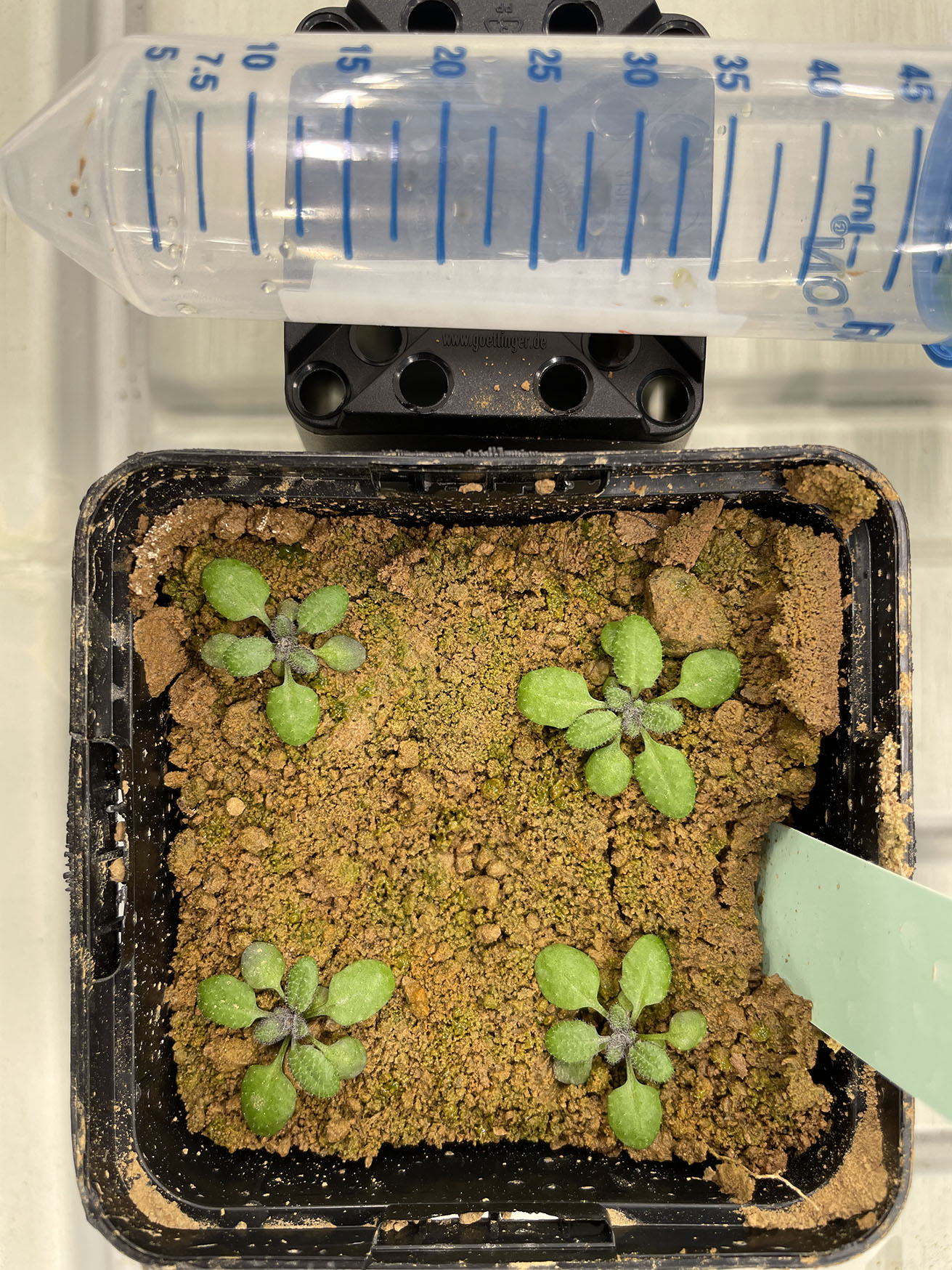

Supplement: Supplementary file 9 — Source data Fig. 5 [file 44318_2024_107_MOESM9_ESM.zip › Figure 5/Figure 5A/H2O/SGN3_small.jpg]
