# Supplementary material for: The Arabidopsis SGN3/GSO1 receptor kinase integrates soil nitrogen status into shoot development
Source: EMBO J. 2024 May 2;43(12):9. doi: 10.1038/s44318-024-00107-3 (PMC11183077; doi:10.1038/s44318-024-00107-3)
Supplement: Supplementary file 11 — Expanded View Figures [file 44318_2024_107_MOESM11_ESM.pdf]

Expanded View Figures

Figure EV1. Detailed analysis of Casparian strip formation and suberization in mutants affected in Casparian strip formation.

(**A**) Upset plot showing the number of differentially expressed genes (DEGs) in $MYB36_{Loop}$ and *myb36-2* roots grown on standard ½ MS agar medium, comparing with the DEGs in Col-0 roots treated with CIF2 from (Fujita et al, 2020). Orange bars represent genes upregulated in $MYB36_{Loop}$, downregulated in *myb36-2*; blue bars represent genes downregulated in $MYB36_{Loop}$, upregulated in *myb36-2*. Red rectangle highlights Schengen pathway activated genes, with GO terms enriched. (**B**) Gene Ontology (GO) term enrichment of DEGs. Note that GO terms enriched in MYB36 $_{Loop}$ downregulated genes are largely overlapping with the GO terms enriched in Schengen pathway activated genes in (**A**). Gene ratio represents the number of DEGs of GO term divided by the total genes in the GO term. + represents upregulated genes, - represents downregulated genes. (**C**) Venn diagram showing the overlap of downregulated genes in $MYB36_{Loop}$roots, downregulated genes in *sgn3-3* roots from (Reyt et al, 2021) and downregulated genes in Col-0 roots treated with CIF2 (Fujita et al, 2020). (**D**) Bar plots showing the distance between root tip and start of string-of-pearls CSD region (left graph) and the distance between root tip and start of xylem (right graph). Plants were grown on standard ½ MS agar medium for 8 days. Data are mean ± SD. Statistical significance of differences with the parental line (WT) was determined using a two-tailed Student's t test. ns; not significant. (**E**) Transmission electron microscopy (TEM) micrograph of 7-day-old anti-GFP immunogold-labeled $MYB36_{Loop}$ #5 roots. Scale bar represents 500 nm. Co; Cortex, CS; Casparian strip, En: Endodermis. (**F**) Maximum projection of a confocal image stack of 7-day-old $MYB36_{Loop}$ plants expressing p*PER64*::PER64-mCherry and p*CASP1*::CASP1-GFP. Scale bars represent 10 µm. Line in overlay depicts the transect used for relative intensity measurements. (**G**) Endodermal cells of plants expressing $MYB36_{Loop}$ stained with Basic fuchsin and Fluorol yellow. Scale bars represent 5 µm. (**H**) Measurement of suberization pattern under mock or 100 nM CIF2 conditions. Error bar represents standard deviation. (**I**) Top-view maximum projection of 7-days old $MYB36_{Loop}$#5 plants treated with 100 nM CIF2 for 24 h before fixing in Clearsee and imaged using a confocal setup. Scale bars represent 2 µm. Different letters depict statistical difference in a one-way ANOVA analysis with Tukey's test ($P < 0.05$). WT represents the parental line (pCASP1:CASP1-GFP) of $MYB36_{Loop}$ plants. Numbers of biological replicates are indicated on graph.

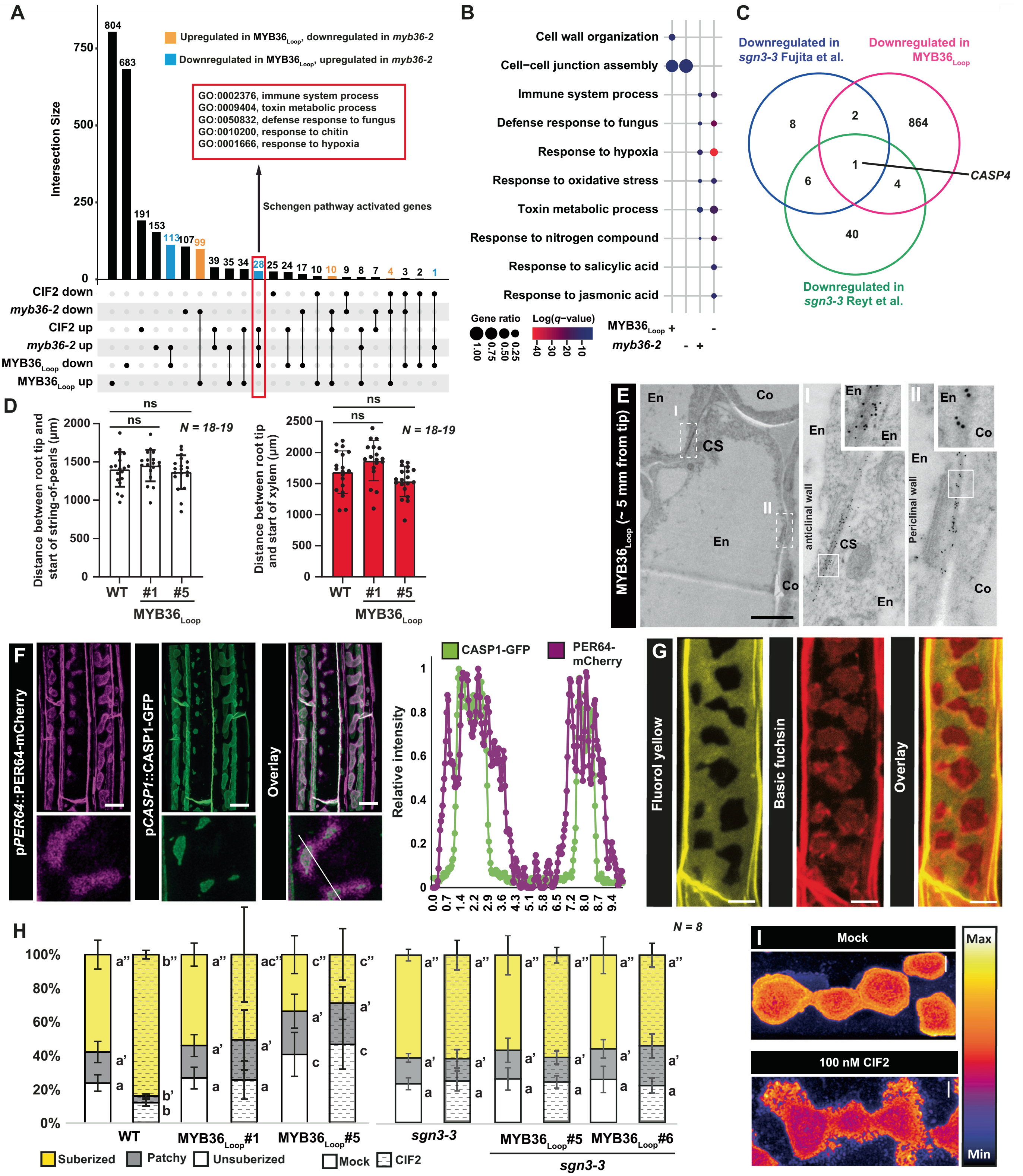
A
Upregulated in MYB36Loop, downregulated in myb36-2
Downregulated in MYB36Loop, upregulated in myb36-2
GO:0002376, immune system process
GO:0009404, toxin metabolic process
GO:0050832, defense response to fungus
GO:0010200, response to chitin
GO:0001666, response to hypoxia
Schengen pathway activated genes
Intersection Size
CIF2 down
myb36-2 down
CIF2 up
myb36-2 up
MYB36Loop down
MYB36Loop up
B
Cell wall organization
Cell−cell junction assembly
Immune system process
Defense response to fungus
Response to hypoxia
Response to oxidative stress
Toxin metabolic process
Response to nitrogen compound
Response to salicylic acid
Response to jasmonic acid
Gene ratio
Log(q−value)
C
Downregulated in sgn3-3 Fujita et al.
Downregulated in MYB36Loop
Downregulated in sgn3-3 Reyt et al.
CASP4
D
Distance between root tip and start of string-of-pearls (µm)
Distance between root tip and start of xylem (µm)
N = 18-19
ns
WT #1 #5
MYB36Loop
E
MYB36Loop (~ 5 mm from tip)
En Co CS
anticlinal wall
Periclinal wall
F
pPER64::PER64-mCherry
pCASP1::CASP1-GFP
Overlay
CASP1-GFP
PER64-mCherry
Relative intensity
G
Fluorol yellow
Basic fuchsin
Overlay
H
N = 8
WT
MYB36Loop#1
MYB36Loop#5
sgn3-3
MYB36Loop#5 MYB36Loop#6
sgn3-3
Suberized Patchy Unsuberized
Mock CIF2
I
Mock
100 nM CIF2
Max
Min

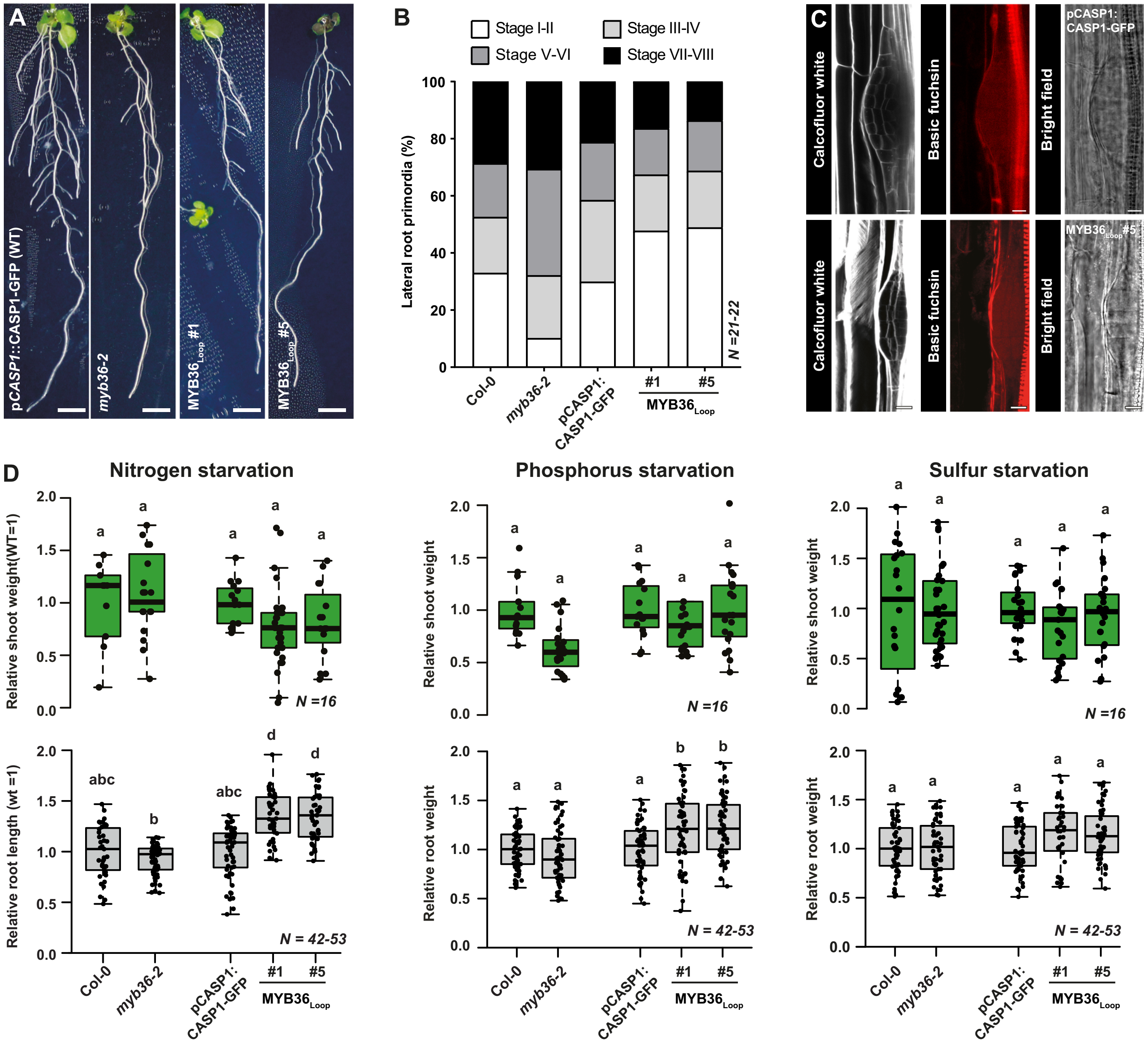

Figure EV2. Detailed physiological and anatomical analysis of mutants affected in Casparian strip formation.

(**A**) 14-day-old seedlings grown on standard ½ MS agar medium. Scale bars represent 5 mm. (**B**) The distribution of different stages of Lateral root primordia (LRP) of 8-day-old plants grown on standard ½ MS agar medium. Combined data from two independent experiments. (**C**) LRP of 8-day-old Col-0 (upper graph) and $MYB36_{Loop}$#5 (lower graph) roots grown on standard ½ MS agar medium, stained with cell wall dye Calcofluor White and the lignin-specific dye Basic Fuchsin. Scale bars represent 10 µm. (**D**) Measurement of shoot (upper graph) and root (lower graph) fresh weight of 2-week-old plants grown under nitrogen, phosphorous (left) or sulfur (right) starvation conditions. The weight was normalized to the changes in the corresponding parental background (Col-0 for *myb36-2* and pCASP1::CASP1-GFP for $MYB36_{Loop}$ lines). For boxplots, the center line in the box indicates the median, dots represent data, the box limits represent the upper and lower quartiles, and the whiskers represent the maximum and minimum values. Different letters depict statistical difference in a one-way ANOVA analysis with Tukey's test ($P < 0.05$). Numbers of biological replicates are indicated on graph.

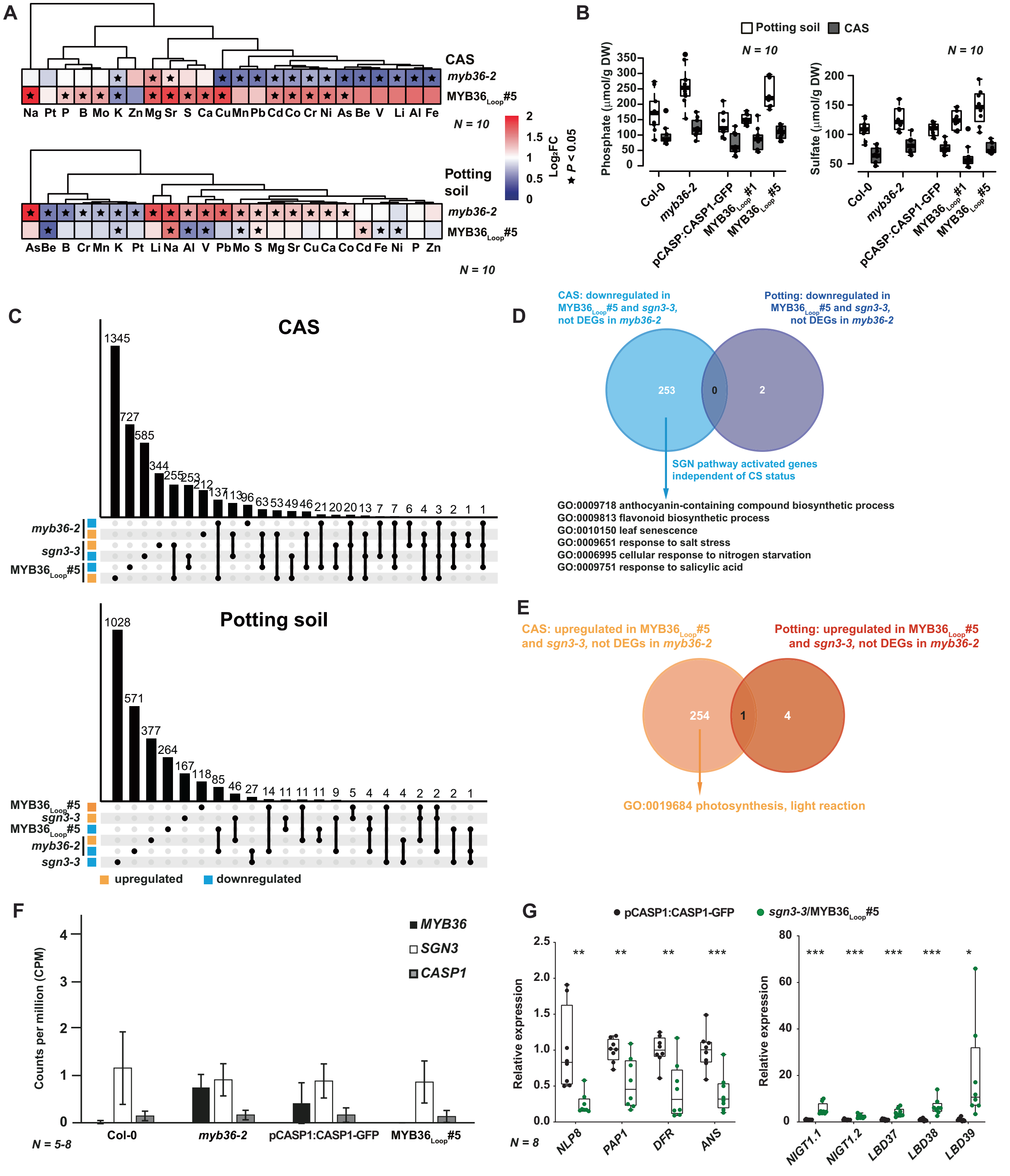
A
CAS
myb36-2
MYB36Loop#5
Na Pt P B Mo K Zn Mg Sr S Ca Cu Mn Pb Cd Co Cr Ni As Be V Li Al Fe
N = 10
2
1.5
1
0.5
0
Log2FC
★ P < 0.05
Potting soil
myb36-2
MYB36Loop#5
As Be B Cr Mn K Pt Li Na Al V Pb Mo S Mg Sr Cu Ca Co Cd Fe Ni P Zn
N = 10
B
Potting soil
CAS
N = 10
N = 10
Phosphate (μmol/g DW)
Sulfate (μmol/g DW)
Col-0
myb36-2
pCASP:CASP1-GFP
MYB36Loop#1
MYB36Loop#5
C
CAS
1345
727
585
344
255
253
212
137
113
96
63
53
49
46
21
20
20
13
7
7
6
4
3
2
1
1
myb36-2
sgn3-3
MYB36Loop#5
Potting soil
1028
571
377
264
167
118
85
46
27
14
11
11
11
9
5
4
4
4
2
2
2
1
MYB36Loop#5
sgn3-3
MYB36Loop#5
myb36-2
sgn3-3
upregulated
downregulated
D
CAS: downregulated in MYB36Loop#5 and sgn3-3, not DEGs in myb36-2
Potting: downregulated in MYB36Loop#5 and sgn3-3, not DEGs in myb36-2
253
0
2
SGN pathway activated genes independent of CS status
GO:0009718 anthocyanin-containing compound biosynthetic process
GO:0009813 flavonoid biosynthetic process
GO:0010150 leaf senescence
GO:0009651 response to salt stress
GO:0006995 cellular response to nitrogen starvation
GO:0009751 response to salicylic acid
E
CAS: upregulated in MYB36Loop#5 and sgn3-3, not DEGs in myb36-2
Potting: upregulated in MYB36Loop#5 and sgn3-3, not DEGs in myb36-2
254
1
4
GO:0019684 photosynthesis, light reaction
F
MYB36
SGN3
CASP1
Counts per million (CPM)
Col-0
myb36-2
pCASP1:CASP1-GFP
MYB36Loop#5
N = 5-8
G
pCASP1:CASP1-GFP
sgn3-3/MYB36Loop#5
Relative expression
NLP8
PAP1
DFR
ANS
NIGT1.1
NIGT1.2
LBD37
LBD38
LBD39
N = 8

 © The Author(s)

Figure EV3. Detailed transcript- and ionome analysis of soil-grown plants affected in Casparian strip formation.

(**A**) Heatmap of 24 element contents in 4-week-old MYB36$_{Loop}$#5 and *myb36*-2 rosettes grown under CAS (upper) or standard potting soil (lower) conditions after normalization to the corresponding parental lines. Star symbols represent significant differences with respective wild-type lines determined using a two-tailed Student's t test ($P < 0.05$). (**B**) Measurement of phosphate (left) and sulfate (right) content in 4-week-old rosettes grown under CAS or standard potting soil conditions. (**C**) Upset plots showing the number of DEGs in MYB36$_{Loop}$ #5, *sgn3-3* and *myb36-2* rosettes grown under CAS (upper) or standard potting soil (lower) conditions. Orange: upregulated; blue: downregulated. (**D**) Venn diagram depicting the overlap of DEGs in rosettes grown under CAS and standard potting conditions and the GO terms specifically enriched among the 254 upregulated DEGs in MYB36$_{Loop}$#5 and *sgn3-3* rosettes, but not DEGs in *myb36-2* rosettes under CAS condition. (**E**) Venn diagram depicting the overlap of upregulated DEGs in *myb36*-2, but not DEGs in *sgn3-3* rosettes between CAS and standard potting condition, and the GO terms enriched. The overlap represents 19 genes activated by the Schengen (SGN) pathway due to defective Casparian strip (CS) formation in both soil conditions. (**F**) Bar plot indicating expression of *MYB36*, *SGN3* and *CASP1* in rosettes of CAS-grown plants. Bars indicate mean values, error bars represent standard deviation. (**G**) RT-qPCR analysis of gene expression level in *sgn3-3/*MYB36$_{Loop}$#5 rosettes compared with the parental line pCASP1::CASP1-GFP grown on CAS with water. The center line in the box indicates the median, dots represent data, the box limits represent the upper and lower quartiles, and the whiskers represent the maximum and minimum values. Combined data from two independent experiments, analyzed by Student's *t* test (*$P < 0.05$, **$P < 0.01$, ***$P < 0.001$). Numbers of biological replicates are indicated on graph.

© The Author(s)

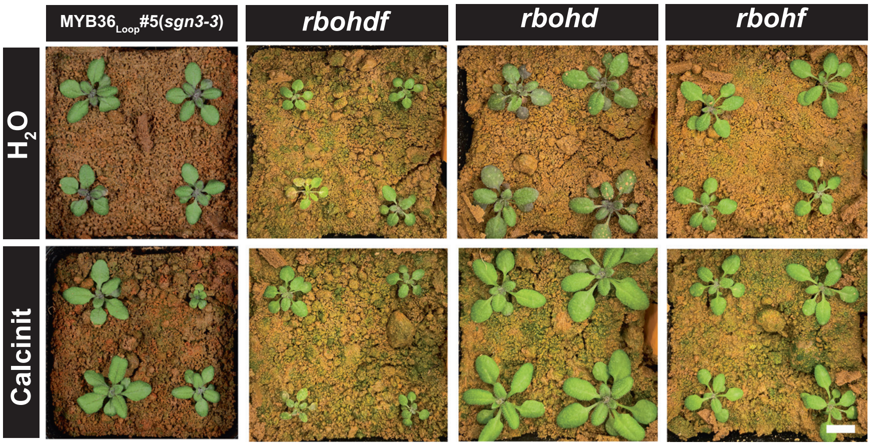

Figure EV4. Shoot phenotypes of plants grown under agricultural conditions.

Four-week-old rosettes from different genotypes grown for one week on ½ MS conditions and transferred to Cologne agricultural soil (CAS) for 3 weeks and watered with water (H_2O) or a Calcinite™ solution containing nitrate ($Ca(NO_3)_2$). Scale bars represent 1 cm. Note that the images of *sgn3-3*/MYB36$_{Loop\#5}$ were derived from an independent experiment from the rest.

 © The Author(s)
